# Revealing regional markers of sauce aroma baijiu in the Chishui River basin: Integrating multi-detection technologies with molecular sensory science and chemometrics

**DOI:** 10.1016/j.fochx.2026.103821

**Published:** 2026-04-03

**Authors:** Long Ma, Tingyao Tu, Mansi Niu, Yuqin Tong, Xingrong Zhao, Junjie Jia, Lei Zheng, Rongkun Tu, Songtao Wang, Suyi Zhang, Caihong Shen

**Affiliations:** a Luzhou Pinchuang Technology Co. Ltd., Luzhou 646000, China; b National Engineering Research Center of Solid-State Brewing, Luzhou 646000, China; c Luzhou Laojiao Co. Ltd., Luzhou 646000, China

**Keywords:** Sauce aroma baijiu, Chishui River basin, Odor activity value, Chemometrics, Regional differentiation

## Abstract

This study employed a multi–technique approach to explore the regional differentiation of sauce aroma baijiu (SAB) within the core production areas of the Chishui River basin. Sensory analysis revealed significant regional differences: right bank (Guizhou) samples showed stronger sauce, qu, roasted, grain, and acidic aromas, while left bank (Sichuan) samples exhibited more intense fruity, floral, and grass notes. Orthogonal partial least squares–discriminant analysis (OPLS–DA) confirmed clear regional differentiation. Aroma recombination experiments demonstrated that the overall aroma profile, especially the characteristic “sauce” aroma, results from the combined contribution of compounds with an odor activity value (OAV ≥ 1), those with OAV < 1, and non–volatile organic acids. Through integrated analysis of OAV, variable importance in projection (VIP > 1), and correlation coefficients, 20 key regional aroma markers were identified that effectively explain the sensory differentiation patterns. These findings provide a scientific foundation for quality control and geographical origin authentication of SAB.

## Highlights

• Revealed sensory differences between the two banks and four regions of Chishui River.
• Identified 20 key regional aroma markers responsible for regional differentiations.
• Verified aroma contributions of OAV ≥ 1, OAV < 1, and non-volatile organic acids to SAB.
• OPLS-DA confirmed significant differentiation among samples from different regions.

## 1 Introduction

Sauce aroma baijiu (SAB), also known as Moutai–flavored baijiu, is one of the most renowned types of baijiu in China. It is highly favored by consumers for its elegant sauce aroma, mellow and sweet taste, soft yet distinct acidity, delicate texture, long aftertaste, and persistent aroma in the empty cup (Guan et al., 2022; Niu et al., 2022). The unique style of SAB is attributed to its distinctive “12987” production process and specific geographical environmental factors (Duan et al., 2024; Gong et al., 2023; Zhang et al., 2024). Due to the unique geographical environment of the Chishui River basin, numerous renowned SAB enterprises are situated in this region (Gong et al., 2023; Li et al., 2024). Notably, the middle reaches of the Chishui River, which form the border between Sichuan Province (SC) and Guizhou Province (GZ), have emerged as the core production area for SAB (Li et al., 2024; Zhang et al., 2024). In 2024, the national output of SAB reached approximately 650,000 kiloliters, generating revenue of 240 billion yuan. The core production area within the Chishui River basin accounted for roughly 53.8% of the production capacity (∼350,000 kiloliters) and 87.5% of the revenue (210 billion yuan). This disparity, where approximately half of the capacity generates nearly 90% of the revenue, is closely linked to the superior quality of SAB in this core area. Flavor components, which constitute about 2% of baijiu, serve as the material basis determining its quality and style characteristics (Hong et al., 2021). These components can be classified into esters, alcohols, acids, aldehydes, ketones, acetals, furans, pyrazines, sulfides, phenols, lactones, and terpenes (Liu & Sun, 2018; Wang, Tang, et al., 2024). The flavor profile of SAB is considered the most complex among all baijiu types. Various hypotheses regarding its main aroma components have been proposed, including 4–ethylguaiacol, pyrazine compounds, furan compounds, and the composite aroma of high–boiling–point acids and low–boiling–point esters (Tang et al., 2020). A consensus has gradually formed that the characteristic aroma of SAB results from the combined effect of multiple flavor compounds. Therefore, characterizing the flavor differences and identifying key aroma compounds among the core production areas within the Chishui River basin is of great significance for the quality control and origin protection of SAB.

“Molecular Sensory Science” is a systematic research methodology pioneered by Professor Peter's team for investigating key food aromas (Lin et al., 2024). The process involves extraction, separation, qualitative and quantitative analysis, threshold determination, odor activity value (OAV) calculation, sensory evaluation, and verification of key aroma components (Lin et al., 2024). This research approach has been widely and successfully applied to identify key flavor compounds in baijiu (Mou et al., 2025; Wang et al., 2023). For instance, Mou et al. (Mou et al., 2025) utilized molecular sensory science techniques to study the key aroma components responsible for floral and fruity notes in SAB. They developed a pervaporation membrane separation technique for flavor extraction and combined gas chromatography–ion mobility spectrometry (GC–IMS)/gas chromatography–mass spectrometry (GC–MS) with sensory analysis to identify 29 potential aroma compounds associated with floral and fruity notes. Through recombination, omission, and addition experiments, they confirmed that 15 flavor compounds are the key contributors to the floral and fruity character in SAB. Gong et al. (Gong et al., 2023) conducted a study on three typical base liquors of SAB from the Chishui River basin (GZ). Using molecular sensory science technology and multivariate statistical analysis, they screened 37 key aroma compounds that differentiate the various types of base liquors.

The flavor composition of SAB is highly complex. Current research on SAB flavors primarily focuses on volatile compounds. The analysis of volatile flavor components requires initial extraction via various pretreatment methods such as direct injection (DI) (Duan et al., 2024; Guan et al., 2022; Li et al., 2024), liquid–liquid extraction (LLE) (Duan et al., 2024; Wang, Gao, et al., 2024; Zhou et al., 2021), and headspace solid–phase microextraction (HS–SPME) (Wu et al., 2023; Zhang et al., 2020; Zhou et al., 2021). The extracts are then analyzed using detection instruments including gas chromatography with a flame ionization detector (GC–FID) (Duan et al., 2024; Li et al., 2024; Lin et al., 2024), GC–MS (Duan et al., 2024; Jia et al., 2024; Zhang et al., 2024), GC-IMS (He et al., 2021; Zhou et al., 2021), and comprehensive two–dimensional gas chromatography–time–of–flight mass spectrometry (GC × GC–TOFMS) (Yan, Chen, Nie, et al., 2020; Yang et al., 2021). Duan et al. (Duan et al., 2024) employed molecular sensory science combined with aroma–active compound reverse verification to identify key aroma compounds in SAB. Using DI and LLE coupled with GC-FID/GC–MS/gas chromatography‑sulfur chemiluminescence detection (GC–SCD), they identified 103 volatile flavor components from two types of SAB. In contrast to volatile compounds, non–volatile compounds in baijiu have received relatively less attention. Non–volatile flavor components are usually analyzed qualitatively and quantitatively using high–performance liquid chromatography (HPLC) (Lyu et al., 2025; Xu, Qiu et al., 2022; Zhao et al., 2024) and ultra–high performance liquid chromatography–tandem mass spectrometry (UHPLC–MS/MS) (Zhao et al., 2025; Zhao et al., 2024). As non–volatile organic acids contribute significantly to the taste and flavor harmony of baijiu (Lyu et al., 2025), research on non–volatile components has primarily focused on them. These organic acids are mainly analyzed by direct injection or derivatization methods coupled with GC–MS, HPLC, and UHPLC–MS/MS (Wang, Jing, et al., 2021; Zhao et al., 2024). Lyu et al. (Lyu et al., 2025) employed direct injection and derivatization methods, combined with HPLC/GC–MS, for the qualitative and quantitative analysis of non–volatile organic acids in their study of flavor differences between northern and southern SABs. Their results showed that the concentration of non–volatile organic acids was generally higher in southern (GZ) samples than in northern ones. This indicates that SABs from different regions exhibit certain regional specificity in their flavor compounds.

In recent years, an increasing number of studies have revealed the regional specificity of baijiu through chemometrics, primarily multivariate statistical analysis (Gong et al., 2023; Li et al., 2024; Lyu et al., 2025; Zhang et al., 2024). Based on molecular sensory science and multivariate analysis, Li et al. (Li et al., 2024) studied the regional dependence of SABs from the Chishui River basin and other northern regions. By screening with variable importance in projection (VIP > 1) and Spearman correlation coefficient (ρ > 0.5), they identified 20 key regional aroma markers for SAB from different regions. Zhang et al. (Zhang et al., 2024) proposed a novel strategy for geographical origin discrimination by collecting empty cup aroma components of SABs *via* in–situ non–targeted flavoromics. They used a partial least squares–discriminant analysis (PLS–DA) model to classify the empty cup aroma data of 15 samples from the Chishui River basin and other northern regions, and the results demonstrated good discriminative ability. However, current research on the regional specificity of SABs mainly focuses on differences between northern and southern regions or different river basins (Li et al., 2024; Lyu et al., 2025; Zhang et al., 2024). There remains a lack of detailed studies examining whether systematic flavor differentiation exists within the core production area of the Chishui River basin, a globally recognized core production area, and identifying the key chemical drivers behind it. Additionally, the role of non–volatile components and low–olfactory contribution compounds in shaping the regional characteristic “sauce” aroma still requires experimental validation.

Therefore, this study aims to focus on the core production area of the Chishui River basin (its left and right banks and four sub–regions), integrating multi–detection technologies with molecular sensory science and chemometrics. It seeks to not only reveal the intricate geographical flavor profile but also verify the synergistic mechanisms of volatile and non–volatile components, as well as high OAV and low OAV compounds, in shaping regional distinctiveness, thereby filling the aforementioned knowledge gap. The details are as follows: (1) Identify the sensory characteristic differences of SAB samples from the core production areas through quantitative descriptive analysis (QDA); (2) Conduct qualitative and quantitative analysis of flavor components in these SABs using GC–FID, GC–MS, HPLC, and UHPLC–MS/MS; (3) Screen and verify the key aroma compounds through OAV analysis, multivariate statistical analysis, correlation network analysis, and aroma recombination experiments; (4) Screen for regional markers of SAB based on VIP > 1, OAV ≥ 1, and Spearman correlation coefficient (|ρ| > 0.5, *P* < 0.05), and explore their respective contributions. Investigating the key aroma compounds and regional markers in SABs from the core production area of the Chishui River basin will help establish a quality evaluation system for SAB and provide a theoretical basis for the protection of its geographical origin.

## 2 Materials and methods

### 2.1 SAB samples

This study aims to elucidate the relatively stable flavor chemical characteristics of SAB produced in the core areas of the Chishui River basin. To achieve this, we implemented a “multi–region, multi–manufacturer, multi–batch” sampling strategy, collecting samples from multiple representative producers within each study area. This design aims to maximize the averaging of random variations caused by individual production batches, annual climate fluctuations, or manufacturer–specific blending techniques by expanding sample size and source diversity. This approach enables more reliable capture and revelation of systematic chemical variation patterns associated with geographical origins.

A total of 40 commercial SAB samples produced in four regions within the Chishui River basin were selected for this study (Table S1). The manufacturers are representative of their respective regions, with stable production processes, substantial production, and sales volumes. Each sample had an alcohol content of 53%vol, including 20 samples (MT1–MT20) from 8 manufacturers in Maotai Town, GuiZhou (GZ, MT), 5 samples (XJ1–XJ5) from Xijiu Town, GuiZhou (GZ, XJ), 9 samples (EL1–EL9) from 2 manufacturers in Erlang Town, SiChuan (SC, EL), and 6 samples (TP1–TP6) from Taiping Town, SiChuan (SC, TP). All samples were stored at 4 °C for subsequent analysis.

### 2.2 Reagents and chemicals

Sodium chloride (NaCl, analytical grade, 99.5%), anhydrous sodium sulfate (Na_2_SO_4_, analytical grade, 99.9%), ammonium formate (NH_4_HCO_2_, MS grade, 99.9%), trifluoroacetic acid (C_2_HF_3_O_2_, MS grade, 99.9%), methanol (HPLC grade, 99.5%), dichloromethane (CH₂Cl₂, HPLC grade, 99.9%), and ethanol (HPLC grade, 99.8%) were purchased from Sinopharm Chemical Reagent Co., Ltd. Hexanal, tert–amyl alcohol (internal standard, IS1), pentyl acetate (IS2), 2–ethylbutanoic acid (IS3), 2–octanol (IS4), L–menthol (IS5), and 154 flavor compounds (listed in Table S2) were purchased from Shanghai Anpu Experimental Technology Co., Ltd. and Aladdin Reagent Co., Ltd. (Shanghai, China), all with purity ≥97%. A C_7_–C_40_ n–alkane mixture (Sigma-Aldrich Co., Ltd) was used for determining linear retention indices (RIs). Water was purified using a Milli–Q system (Millipore, Bedford, MA, USA).

### 2.3 Sensory evaluation analysis

#### 2.3.1 Sensory panel

Sensory analysis was conducted following previous references (Duan et al., 2024; Gong et al., 2023) and the National Standard of China (GB/T 10345–2022), with minor modifications. The panel consisted of 12 assessors (6 males and 6 females, aged 26–33), including 4 national–level and 4 provincial–level baijiu judges, and 4 certified sensory evaluators (Grade II or above). All assessors had extensive experience in sensory assessment, including odor threshold determination and QDA.

#### 2.3.2 Descriptive profile tests

Sensory description training was conducted with reference to literature (Duan et al., 2024) and the National Standard of China (GB/T 10781.4–2024). Panelists were trained to evaluate eight aroma attributes: sauce, qu, roasted, grain, fruity, floral, grass, and acidic. The intensity of each attribute was rated on a 5–point scale (0 = none, 1 = weak, 2 = moderate, 3 = strong, and 4 = very strong). Standard references included 5 g soy sauce (sauce aroma, 4.0), 5 g high–temperature Daqu powder (qu aroma, 4.0), 800 mg/L 2–ethyl-3,5–dimethylpyrazine in 10%vol ethanol (roasted aroma, 4.0), a mixture of 1.3 g/L 3–methylbutyl acetate, 440 mg/L ethyl butanoate, and 700 mg/L ethyl hexanoate in 10%vol ethanol (fruity aroma, 4.0), 2 g/L 2–phenethyl alcohol in 10%vol ethanol (floral aroma, 4.0), 5 g vinegar (acidic aroma, 4.0), 10 g steamed sorghum (grain aroma, 4.0), and 1 g/L hexanal in 10%vol ethanol (grass aroma, 4.0). All sensory evaluations were performed in triplicate.

#### 2.3.3 Determination of odor thresholds

Odor thresholds were determined in a 53%vol ethanol solution as the matrix, following Chinese National Standards (GB/T 33406–2016 and GB/T 22366–2022) with slight modifications. Compounds with initial concentrations exceeding 20 times their recognition threshold were evaluated. Gradient dilutions (factor of 2 or 3) were prepared, and each concentration level was presented as a set of four samples, two containing the compound and two blanks (matrix only). Each sample was labeled with a random 3–digit code. Assessors were asked to identify the different samples in each set through sniffing. The threshold was determined as the concentration at which 67% of the panelists correctly identified the sample, based on linear fitting curves.

### 2.4 Qualitative and quantitative analysis of aroma compounds

Given the wide variety and concentration range of flavor compounds in SAB, multiple pretreatment methods and analytical instruments were employed for comprehensive analysis.

#### 2.4.1 Direct injection combined with GC-FID (DI-GC-FID)

A Shimadzu GC2030 system equipped with an FID was used to identify and quantify the volatile compounds at higher concentrations (≥ 5 mg/L). Samples were prepared as referenced in our previous work (Niu et al., 2025). 10 μL of a mixed internal standard solution (2000 mg/L each of IS1 and IS2, or IS3) was added to a 1 mL of baijiu. After mixing the sample, the autosampler automatically injects 1 μL into the GC–FID for analysis. High–purity nitrogen gas (purity 99.999%) was used as the carrier gas at a flow rate of 0.8 mL/min. The injector and detector temperatures were set to 250 °C, with a split ratio of 40: 1. The initial oven temperature was set at 35 °C and held for 3 min, followed by a temperature increase of 3 °C/min to 80 °C, where it was held for 1 min. The temperature was then ramped up at 5 °C/min to 100 °C and held for 3 min, then further increased at 5 °C/min to 160 °C and held for 2 min. Finally, the temperature was increased at a rate of 10 °C/min to 230 °C and held for 18 min.

Compounds 1–4, 7–9, 13–14, 18, 36–37, 40–41, 45, 48, 51, 53–62, 73–75, 77, 95–100, 102–104, 107–109, 119–120, and 122 (Table S2) were analyzed using DB–WAX chromatography columns (60 m × 250 μm i.d., × 0.25 μm film thickness, Agilent Technologies) with reference to internal standards IS1 and IS2. Due to the tailing peak of the volatile organic acids (Compounds 80–89) on the DB–WAX column, they were analyzed on a DB–FFAP column (60 m × 250 μm i.d., × 0.25 μm film thickness, Agilent Technologies) with reference to internal standards IS3. Under the same injection conditions as the baijiu, the retention index (RI) was calculated using a mixture of C_7_–C_40_ n–alkanes. Compounds were identified by comparing retention indices (RIs) with standards (S). Calibration curves were constructed using mixed standard solutions at various concentrations (i.e., 4000, 2500, 2000, 1500, 1000, 500, 250, 200, 150, 100, 50, 25, 10, and 5 mg/L). The coefficient of determination (R^2^), limits of detection (LOD), limits of quantification (LOQ), and recovery rates were determined for each compound. All experiments were repeated three times.

#### 2.4.2 HS***–***SPME arrow combined with GC–MS

Volatile compounds at lower concentrations were extracted using an automated headspace system (PAL RTC–1600, Guangzhou Ingenious Laboratory Technology Co., Ltd.) with a 120 μm DVB/CAR/WR/PDMS SPME arrow. Analysis was performed on a Shimadzu GCMS–QP2020NX system equipped with a DB–FFAP column (60 m × 250 μm i.d., × 0.25 μm film thickness, Agilent Technologies). The pretreatment conditions for the sample are based on the previous references (Zhang et al., 2020), with slight modifications. A 5 mL SAB sample (diluted to 10%vol ethanol), 2 g of NaCl, and a mixed internal standard (IS4 and IS5, 300 mg/L) were added to a 20 mL headspace glass vial. Before extraction, the SPME arrow should be equilibrated at 250 °C for 5 min, then extract and adsorb at 50 °C for 45 min. After extraction, insert the SPME arrow into the GC–MS injector for analysis. High–purity Helium (99.999% purity) was used as a carrier gas with a flow rate of 1.0 mL/min, in a splitless mode. The injector and detector temperatures were 250 °C, the initial oven temperature was 40 °C (held for 2 min), which was ramped to 120 °C at 3 °C/min (held for 3 min), then increased to 150 °C at 3 °C/min, and then raised to 230 °C at 6 °C/min (held for 10 min). The MS spectra were recorded in the electron ionization mode (EI) at an ionization energy of 70 eV. The transfer line and ion source temperatures were 250 °C and 230 °C, respectively. During the identification of volatiles, the full scan mode (SCAN) with a scanning range of 35–500 *m*/*z* and a scanning period of 0.3 s was used.

For compounds 5–6, 10–12, 15–17, 19–35, 38–39, 42–44, 46–47, 49–50, 52, 63–72, 76, 89, 101, 105–106, 110–118, 121, 123, and 143–154 with lower concentrations (Table S2), HS–SPME arrow combined with GC–MS analysis was used. Identification was based on mass spectra (MS, NIST20 library), as well as by matching the retention index (RI) of the unknown compounds with those of reference standards (S). Quantification was performed using ion chromatograms and calibration curves from standard solutions at various concentrations (i.e., 5000, 2500, 2000, 1500, 1000, 500, 250, 200, 150, 100, 50, 25, 10, 5, and 1 μg/L). All experiments were repeated three times.

#### 2.4.3 LLE combined with GC–MS

Three furanones (compounds 124–126 in Table S2) were extracted by LLE based on a previous method (Wang, Gao, et al., 2024) with modifications. SAB samples (50 mL, diluted to 10%vol ethanol) were saturated with NaCl and extracted three times with CH₂Cl₂. The combined organic phases were dried over anhydrous Na₂SO₄ and concentrated to 1 mL under nitrogen. 10 μL IS4 was added, and 1 μL of the concentrated solution was injected into GC–MS for analysis each time, with the same GC–MS configuration as mentioned above. The injector temperature is 250 °C, with a solvent delay of 5 min, column flow rate of 2.0 mL/min, using splitless mode. The initial oven temperature is 45 °C, held for 2 min, then ramped at 6 °C/min to 230 °C and held for 10 min. The MS spectra were recorded in the electron ionization mode (EI) at an ionization energy of 70 eV. The transfer line and ion source temperatures are set at 250 °C and 230 °C, respectively. Selective ion monitoring (SIM) mode is used for data acquisition, with 128, 142, and 128 selected as quantitative ion fragments for 2,5–dimethyl–4–hydroxy–3(2H)–furanone (HDMF), 2–ethyl–4–hydroxy–5–methyl–3(2H)–furanone (HEMF), and sotolon, respectively. All experiments are repeated three times.

#### 2.4.4 Determination of non***–***volatile organic acid by HPLC

Three organic acids (compounds 78, 90–91 in Table S2) were analyzed using a Shimadzu LC–20A HPLC system with a UV detector (210 nm) and an Agilent Polaris C18–A column (250 mm × 4.6 mm, 5 μm), following a described method (Zhao et al., 2024). After passing the SAB sample through a 0.22 μm microfiltration membrane, the sample is directly injected with a 10 μL injection volume. The column temperature was maintained at 30 °C, and the flow rate was 0.5 mL/min. Mobile phase A is a 0.1% (mass fraction) phosphoric acid aqueous solution, and mobile phase B is methanol. The elution pump program is as follows: 0–10 min, 100% A; 10–10.5 min, 100–90% A; 10.5–16 min, 90% A; 16–16.5 min, 90–50% A; 16.5–45.0 min, 50% A; 45.0–45.5 min, 50%–100% A; 45.5–60.0 min, 100% A. Qualitative analysis was performed using reference standards (S), and quantitative analysis was conducted by external standard method. Under the same analytical conditions as the baijiu samples, a mixed standard solution of three organic acids was prepared in 53%vol ethanol to the following concentrations: 2500, 1500, 1000, 500, 250, 100, 50, 25, 10, 5, and 1 mg/L. Calibration curves were plotted based on the peak area of the selected flavor compounds versus their corresponding concentrations, with all experiments repeated three times.

#### 2.4.5 Determination of the other three non***–***volatile organic acids and pyrazines by UHPLC–MS/MS

Three higher fatty acids (compounds 92–94 in Table S2) and sixteen pyrazines (compounds 127–142 in Table S2) were analyzed using a Shimadzu LCMS–8045 triple quadrupole mass spectrometer. Higher fatty acids were separated on a Waters ACQUITY BEH C18 column (100 mm × 2.1 mm, 1.7 μm) with a sample injection volume of 5 μL. During analysis, the column temperature was maintained at 40 °C with a flow rate of 0.1 mL/min. Mobile phase A consisted of 10 mmol ammonium formate in methanol, while mobile phase B was 10 mmol ammonium formate in water, with an isocratic elution ratio of A: B = 88: 12. The MS conditions were as follows: electrospray ionization (ESI) source in negative ion mode, interface temperature at 300 °C, desolvation line (DL) temperature at 250 °C, desolvation temperature at 526 °C, heating block temperature at 400 °C, nebulizer flow rate at 3 L/min, heater flow rate at 10 L/min, dryer flow rate at 10 L/min, and mass spectrometry scan range of 40–400 *m*/*z*.

The pyrazine compounds were analyzed following previous methods (Xiang et al., 2025; Yan et al., 2021) using a Phenomenex Gemini C6-Phenyl 110 Å liquid chromatography column (250 mm × 4.6 mm, 5 μm). The liquid chromatography conditions were as follows: column temperature at 40 °C, mobile phase A consisted of 0.1% trifluoroacetic acid in water (containing 0.1% formic acid), while mobile phase B was methanol, gradient elution program: 0–22 min, 93%–80% A; 22–33 min, 80%–93% A; 33–40 min, 93%–30% A; 40–47 min, 30%–93% A; 47–50 min, 93% A; flow rate was 0.6 mL/min, and injection volume was 20 μL. Mass spectrometry conditions were as follows: electrospray positive ion (ESI+), multi–reaction monitoring mode (MRM); ion spray voltage at 4.0 kV; nebulizing gas: nitrogen at 3.0 L/min; drying gas: nitrogen at 10 L/min; heating gas: air at 10 L/min; collision gas: argon; desolvation tube temperature at 250 °C; heating module temperature at 400 °C; interface temperature at 300 °C.

Three higher fatty acids and sixteen pyrazines were qualitatively analyzed using standards (S) and quantified by the external standard method. Under the same analytical conditions as the SAB samples, a mixed standard solution of three higher fatty acids was prepared in 53%vol ethanol at the following concentrations: 25000, 10000, 5000, 2500, and 1000 μg/L. Similarly, a mixed standard solution of sixteen pyrazines was prepared in 53%vol ethanol at the following concentrations: 25000, 15000, 10000, 5000, 2500, 1000, 500, 250, 100, 50, and 10 μg/L, and all experiments were repeated three times. Given the complexity of flavor components in baijiu, matrix effects (MEs) are prevalent in baijiu substance detection. During the detection process, specific methods are typically employed to mitigate (e.g., dilution method, extraction method) and compensate for (e.g., matrix matching method, internal standard method) MEs. In this study, based on the external standard method employed for quantitative detection using UHPLC–MS/MS, we adopted matrix matching to compensate for MEs (Lian et al., 2024). Quantitative standard curves for higher fatty acids (with linoleic acid as an example) and pyrazines (with 2,3,5,6–tetramethylpyrazine as an example) were tested using MT–1 as the matrix. The slope of the matrix–matched standard curve was compared with that of the 53%vol ethanol aqueous solution curve. MEs were quantified using the following formula, and compensation for MEs, along with methodological evaluation, was performed based on the matrix matching approach. Typically, MEs < 20% indicate a weak MEs influence in the quantification method (Kwon et al., 2012).

$$\mathrm{MEs}/\%=|K_{1}-K_{2}|/K_{2}\times 100$$

where K_1_ represents the slope of the matrix matching standard curve, K_2_ represents the slope of the solvent curve.

### 2.5 Aroma recombination

Recombination models were prepared by adding flavor compounds at their measured concentrations to a 53%vol ethanol solution. After equilibration, the recombinant and original samples were subjected to QDA to assess similarity.

### 2.6 Statistical analysis

Statistical analysis was performed using SPSS 27 (SPSS Inc., USA). Data are presented as mean ± standard deviation (*n* = 3). Significance was determined at *P* < 0.05. Data visualization, including bar charts and radar plots, was created using Origin 2021 (OriginLab Inc., USA). Cluster heatmaps and correlation analysis were performed using the OmicStudio cloud platform (https://www.omicstudio.cn), and the correlation network was visualized with Gephi–1.10.1 (Université Paris VIII, France). Principal component analysis (PCA) and orthogonal partial least squares–discriminant analysis (OPLS–DA) were conducted using SIMCA 14.1 (Umetrics, Sweden).

## 3 Results and discussions

### 3.1 Aroma profiling analysis

**Fig. 1:**
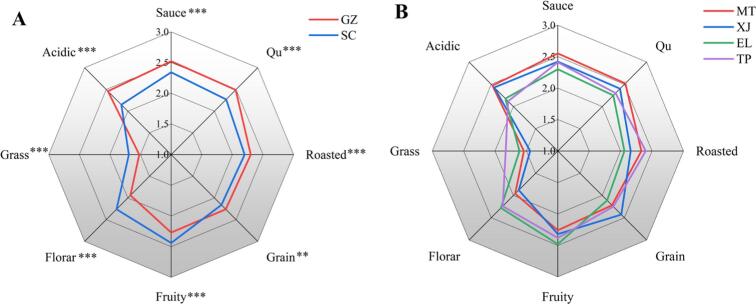
Sensory analysis of sauce aroma baijiu (SAB) samples from the core production area in the Chishui River basin. (A) Average sensory evaluation of samples from both sides of the Chishui River, (B) Average sensory evaluation of samples from four regions. Significance was calculated using the *t*-test, with *P* < 0.05 considered significant.*: *P* < 0.05, **: *P* < 0.01, ***: *P* < 0.001, NS: not significant.

QDA was performed on all samples (n = 3) across eight aroma attributes: sauce, qu, roasted, grain, fruity, floral, grass, and acidic. PCA analysis was performed based on the aroma evaluation scores of each sample (Fig. S1A and B). Samples from the right bank (GZ) clustered in the lower right quadrant, whereas those from the left bank (SC) were positioned in the upper left (Fig. S1A). Fig. S1B reveals substantial overlap in the distribution of samples from EL town and TP town in SC, indicating many similarities in aroma characteristics. Similarly, samples from MT town and XJ town in GZ show high sensory similarity. The average results of QDA for samples from both banks of the Chishui River and four regions were plotted as flavor radar charts (Fig. 1A and B). As shown in Fig. 1A, samples from both banks showed extremely significant differences (*P* < 0.001) in sauce, qu, roasted, grain, fruity, floral, grass, and acidic aroma, followed by significant differences (*P* < 0.01) in grain aroma. Samples from GZ demonstrate higher intensity in sauce, qu, roasted, grain, and acidic aroma, while samples from SC exhibit stronger fruity, floral, and grass aromas. Further analysis of the four sub–regions (MT, XJ, EL, and TP town) indicated distinct sensory profiles (Fig. 1B). MT samples scored highest in sauce, qu, and acidic attributes; XJ samples exhibited the strongest grain aroma; EL samples were dominant in floral and fruity notes; and TP samples showed the most pronounced roasted and grass characteristics. It is worth mentioning that samples from GZ scored higher than those from SC in terms of sauce, qu, and acidic aromas. Correspondingly, MT and XJ samples scored higher than those from the EL and TP towns in these aroma categories. The same pattern was observed for floral, fruity, and grass aromas. However, this regulation was not found in the scores for grain and roasted aromas.

### 3.2 Identification and quantitative analysis of aroma compounds

**Fig. 2:**
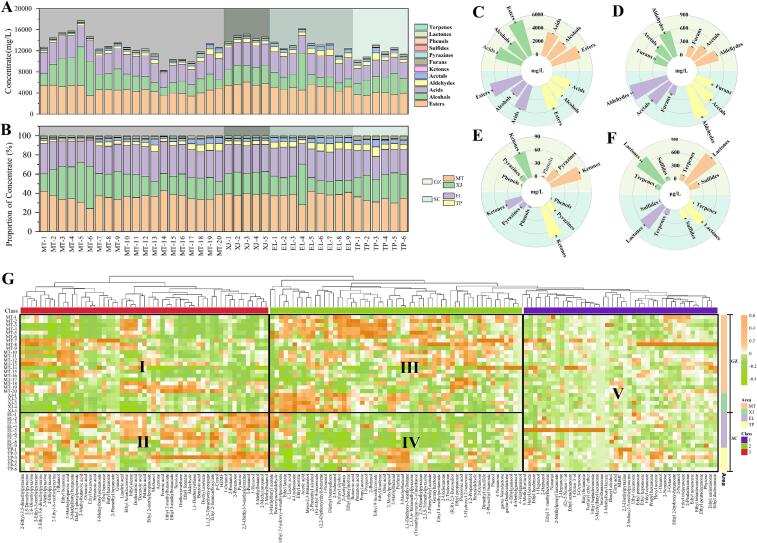
Distribution of various flavor compounds in SABs from the core production area of the Chishui River basin. (A) Distribution of the content of various flavor compounds in all samples; (B) Distribution of the percentage content of various flavor compounds in all samples; Average content distribution of different types of compounds in samples from four regions in the core production area of the Chishui River Basin (C) Esters, alcohols, and acids; (D) Aldehydes, acetals, and furans; (E) Ketones, pyrazines, and phenolic substances; (F) Lactones, sulfides, and terpenes in samples from four regions in the core production area of the Chishui River basin; (G) Cluster heatmap of 154 flavor compounds in SABs from the core production area of the Chishui River basin.

Quantitative analytical methods for 154 flavor compounds were established according to the methodology described in Section 2.4, as shown in Table S2. All quantitative standard curves exhibited good linearity, with corresponding R^2^ values ranging from 0.9710 to 0.9999. The sample spiked recovery ranged between 81.37% and 116.88%. It is noteworthy that the standard curves for linoleic acid and 2,3,5,6–tetramethylpyrazine in the matrix also exhibited good linear relationships, with corresponding coefficient of determination (R^2^) values of 0.9953 and 0.9983, respectively (Fig. S2). Based on the matrix compensation method, the matrix effects for both compounds were calculated to be 12.75% and 7.26%, respectively, both below 20%, indicating negligible MEs (Ferrer et al., 2011). Based on the quantitative methods, the concentrations of various flavor compounds in 40 samples from the core production area of the Chishui River basin were obtained (Table S3–S6). The total content of aroma compounds and the content of various types of flavor compounds in different samples varied significantly (Fig. 2A). MT–5 and MT–14 were the samples with the highest and lowest total aroma compound content among all samples, respectively. However, their proportions of various compounds were similar to those of other samples from MT town (Fig. 2B). This indicates that the aroma characteristics of the baijiu are not only related to the content of flavor compounds but also to the proportions of various flavor compounds.

Esters, alcohols, and acids were the three types of compounds with the highest total content among all types of flavor compounds, which is consistent with the report by He et al. (He et al., 2022). The esters mainly provide floral, fruity, and sweet aromas for baijiu. The types and concentrations of esters in different baijiu have a significant impact on the product style (Xu, Zhao, et al., 2022). The alcohols can bring some floral and fruity aromas and mellow sweetness to baijiu, and a moderate amount of higher alcohols can play a role in helping flavor in SAB (Wang, Zhao, et al., 2020). The acids mainly contribute to the taste, balancing the flavor of the baijiu and coordinating the aroma (Lyu et al., 2025). Some short–chain volatile acid compounds have the fragrance of fruity–like acid and cellar aroma. Notably, the four major ester compounds—ethyl acetate, ethyl butanoate, ethyl hexanoate, and ethyl lactate—along with the four major acid compounds—acetic acid, butanoic acid, hexanoic acid, and lactic acid—are critical aroma indicators in baijiu, significantly influencing its aromatic characteristics and quality (Jia et al., 2024; Liu & Sun, 2018). Although lactic acid is a non–volatile organic acid, it serves not only as the primary source of acidity in SABs but also affects microbial activity during fermentation and the formation of aromatic compounds (Lyu et al., 2025). As shown in Table S3–S6, the lactic acid content in the samples ranged from 727.35 mg/L to 1761.04 mg/L, which aligns closely with the lactic acid concentration distribution reported by Lyu et al. (Lyu et al., 2025) for SABs from Guizhou and northern China (877–2133 mg/L). As shown in Fig. 2C, the average content of esters (*P* < 0.05), alcohols (*P* < 0.001), and acids (*P* < 0.01) in all samples from GZ was higher than that in SC. However, the distribution of esters in the samples from the four regions varies. The XJ samples have the highest average content of esters, followed by EL and MT towns, with TP town having the lowest content. The high ester content in XJ Town is primarily attributed to ethyl acetate (3136.67 mg/L) and ethyl lactate (2205.44 mg/L), both of which significantly exceed levels observed in the other three production regions (Table S3–S6). Li et al. (Li et al., 2024) found regional variations in ethyl acetate concentrations among SAB producers. Northern regions recorded ethyl acetate levels as high as 3071.45 mg/L, markedly exceeding southern production areas—a disparity likely linked to brewing techniques and sorghum cultivars. Generally, northern SAB made from japonica sorghum exhibits higher ethyl acetate content compared to those produced in Sichuan–Guizhou regions using glutinous sorghum as raw material (Wang, Tang, et al., 2024). Furthermore, this study revealed that the differences in ester profiles between samples from XJ Town and EL Town were primarily observed in ethyl acetate and ethyl lactate (Table S3–S6). The concentration distributions of ethyl butanoate (15.3–101.41 mg/L) and ethyl hexanoate (25.63–123.96 mg/L) in all samples were consistent with the findings summarized by Xu et al. (Xu, Zhao, et al., 2022), specifically ethyl hexanoate (14.5–1293 mg/L) and ethyl butanoate (19.2–718 mg/L). The distribution pattern of total acid content is the same as that of esters, namely XJ > EL > MT > TP. Interestingly, the distribution patterns of the four major esters and four major acids not only exhibit consistency with the total acid and total ester concentrations along both banks of the Chishui River, but also show identical distribution patterns across the four subregions. Duan et al. (Duan et al., 2024) found that esters and acids make important contributions to the acidic, fruity, grain, and qu aroma of SABs. The high content of esters and acids in XJ samples may be related to their higher scores in grain, acidic, and qu aroma (Fig. 1B). Regarding alcohols, MT and XJ towns have similar total alcohol content, followed by TP town, with EL town having the lowest content (Fig. 2C).

As depicted in Fig. 2D, the total content of aldehydes, acetals, and furans is second only to that of esters, alcohols, and acids. Their distribution pattern is exactly opposite to that of esters, alcohols, and acids, with significantly higher levels on the SC than on GZ (*P* < 0.01). Aldehydes and acetals mostly possess grass, fruity, and pungent odors (Wang, Tang, et al., 2024). Moreover, acetals are important indicators of baijiu's aging (Sun et al., 2022), which can enhance the softness and aroma of the baijiu. The distribution of aldehydes and acetals across the four regions indicates that EL > TP > XJ > MT, aligning with the fruity aroma scores presented in Fig. 1B. 1,1–diethoxyethane is the most abundant flavor component among acetal compounds, with concentrations ranging from 91.71 to 895.72 mg/L (Table S3–S6). Across both banks of the Chishui River and four sub–regions, its average concentration distribution was consistent with that of total acetals, and matched the fruity aroma score trends of the two banks and four sub–regions shown in Fig. 1. This further confirms a close correlation between 1,1–diethoxyethane and fruity aroma characteristics (Wang, Tang, et al., 2024). As a chromatographic skeleton component of SAB, existing studies have reported that acetal content varies widely, ranging from 6.96 to 713.21 mg/L (Fan, Shen, et al, 2011; Li et al., 2024). Such variation may be related to sample sources and detection methods. Niu et al. (Niu et al., 2020) discovered during their research on ester compounds in SAB that certain flavor compounds exhibited marked variations in detection levels under different pretreatment protocols within the same sample. The furans mainly exhibit caramel, roasted, nutty, and sweet aromas (Wang, Gao, et al., 2024; Wang, Tang, et al., 2024). The content of furans in the TP samples was the highest, followed by that in XJ town, then MT town, and EL town had the lowest concentration. Among furan compounds, furfural was the most abundant, with a content ranging from 27.29 to 427.63 mg/L (Table S3–S6), which was generally consistent with the range of furfural content (33.09–395.52 mg/L) reported in previous studies on SAB (Li et al., 2024; Zhang et al., 2020). Regional variations were observed in furfural concentrations: TP Town recorded the highest average content (381.263 mg/L), while EL Town showed the lowest (211.104 mg/L). This distribution pattern was highly consistent with the sensory scores of roasted aroma in the two producing areas shown in Fig. 1B. It is therefore inferred that furfural is a key furan compound contributing to the roasted aroma of SAB. Notably, furfural concentrations in MT Town (214.471 mg/L) and XJ Town (280.51 mg/L) showed no significant correlation with roasted aroma intensity, indicating that the distinctive roasted aroma profile in SAB results from the synergistic interaction of multiple flavor compounds rather than being solely determined by furfural. Studies have shown that furfural is an important differential aroma compound in the *Chuntian* base liquor and *Jiaodixiang* base liquor of the SABs (Wang, Zhao, et al., 2020).

The concentrations of ketones, pyrazines, phenols, lactones, and sulfides in the GZ samples were significantly higher than those in the SC samples (*P* < 0.001). Similar to aldehydes, ketones make positive contributions to the grass of baijiu (Sun et al., 2022; Wang et al., 2015). The pyrazines and sulfides typically have low thresholds and strong aromas (Yan, Chen, Nie, et al., 2020; Yan et al., 2021). The pyrazines usually exhibit roasted, nutty, and grass odors (Wang et al., 2015), while sulfides commonly possess flavors of coffee and vegetables (Duan et al., 2024). In SABs, sulfides can reduce the threshold of fruity aroma, thereby increasing its intensity (Yan, Chen, Nie, et al., 2020). The phenols in baijiu contribute to smoky, cellar–aged, and grain aromas (Wang, Ma, et al., 2021). The formation of the lactones may be related to the Maillard reaction, and they typically exhibit pleasant aromas such as nutty, coconut, milky, and sweet aromas (Wang, Tang, et al., 2024). The pyrazines, phenols, lactones, and sulfides have the highest content in MT samples (Fig. 2E and F). Most of these compounds are products of the Maillard reaction and can contribute to the sauce and roasted aromas in baijiu (Wang et al., 2015). The high scores of sauce and roasted in MT samples shown in Fig. 1B may be related to the content of these compounds. Although pyrazine compounds do not directly contribute to the sauce aroma profile, they can significantly enhance the typical aroma and exert a positive regulatory effect on the overall aroma quality of the liquor (Sun et al., 2025). For the detection and analysis of pyrazines in baijiu, commonly used sample pretreatment techniques include LLE (Wang et al., 2014), SPME (Sha et al., 2017), and stir bar sorptive extraction (SBSE) (Niu et al., 2015), followed by qualitative and quantitative analysis using GC–MS. Although the nitrogen‑phosphorus detector (NPD) exhibits a specific response toward nitrogen–containing compounds, its sample pretreatment processes which can significantly reduce analytical repeatability, are cumbersome and involve multiple manual steps (Wu & Xu, 2013). Compared with conventional GC–MS, UHPLC–MS/MS can greatly simplify the sample pretreatment procedure. Meanwhile, the electrospray ionization source provides efficient and stable ionization, facilitating the accurate identification of structurally complex compounds, and is thus more suitable for the quantitative analysis of pyrazines in complex matrices (Yan, Chen, He, et al., 2020). 2,3,5,6–tetramethylpyrazine was the most abundant among pyrazine compounds. Our study determined an average mass concentration of 8.88 mg/L for 2,3,5,6–tetramethylpyrazine across all samples (Table S3–S6), which generally exceeds the maximum value of 4.63 mg/L reported by existing gas–phase methods (Sun et al., 2025). This discrepancy may be attributed to variations in sample sources and analytical methodologies. Pyrazines are basic compounds, which reduces their volatility in the acidic matrix of baijiu (Wang et al., 2014), leading to certain losses during detection by gas–phase detection methods. It is worth mentioning that TP town ranked second in terms of pyrazines and sulfides, only after MT town, which corresponds to its higher sauce and roasted aroma scores. Given the limitations of the pretreatment methods and detection techniques employed in this study, only one sulfur–containing compound, dimethyl trisulfide, was identified. The concentration ranging from 11.61 to 671.68 μg/L (Table S3–S6). Notably, despite variations in pretreatment and detection methods, the distribution range of dimethyl trisulfide in SAB closely matches findings reported in previous studies on Sichuan and Guizhou SAB samples (Duan et al., 2024; Yan, Chen, Nie, et al., 2020), indicating that the quantitative method used in this study possesses certain universality. The ketones showed the highest content in TP town (Fig. 2E), and the highest grass score in TP town might also be related to the content of the ketones. It is worth mentioning that TP town ranked second in terms of pyrazines and sulfides, only after MT town, which corresponds to its higher sauce and roasted aroma scores. The terpenes can bring an elegant and delicate taste to SAB (Wang et al., 2015), but their content showed no significant difference among the core production areas in the Chishui River basin.

Hierarchical cluster analysis (HCA) and heatmaps of 154 compounds revealed three distinct abundance patterns (Class 1–3) with clear regional segregation (Fig. 2G), with colors ranging from light to dark representing content levels from low to high. Class 1 contained 55 flavor compounds (red), class 2 contained 56 flavor compounds (green), and the remaining 43 flavor compounds were in class 3 (purple). Obvious differences were observed between samples from the two banks of the Chishui River in classes 1 and 2. The content of compounds in samples from GZ (Area I) was generally lower than in SC (Area II). These flavor components are mainly esters, alcohols, aldehydes, and acetals that contribute floral, fruity, and grass notes in baijiu (e.g., ethyl 3–methylbutanoate, ethyl hexanoate, hexanol, acetaldehyde, and 1,1–diethoxyethane) (Tang et al., 2020). This corresponds to the sensory differences in floral, fruity, and grass notes between samples from the two banks shown in Fig. 1A. Studies have shown that ethyl 3–methylbutanoate, ethyl hexanoate, and acetaldehyde are key aroma components contributing to the floral and fruity notes in SAB (Mou et al., 2025). Acetaldehyde and 1,1–diethoxyethane have the aroma of fresh–cut grass and green apples (Aguera et al., 2018), and their content shows a significant positive correlation with the intensity of grass in SAB. The content of hexanol also positively correlates with the intensity of fruity (Li et al., 2024). The content of class 2 compounds on the GZ (Area III) is generally higher than SC (Area IV), including some acids, pyrazines, phenols, and furans, such as butanoic acid, 2,3,5–trimethylpyrazine, 2,3,5,6–tetramethylpyrazine, 4–ethylguaiacol, and furfuryl alcohol. Most of these compounds are products of the Maillard reaction, and many have been considered as the main aroma components of SAB (Tang et al., 2020). Studies have shown that the content of butanoic acid is significantly correlated with the sauce in three typical base liquors of SAB (Gong et al., 2023). The content of 2,3,5–trimethylpyrazine and 2,3,5,6–tetramethylpyrazine is related to the roasted and qu in SAB (Li et al., 2024). 4–ethylguaiacol has a sauce–like aroma when its concentration is high (Wei et al., 2020). Furfuryl alcohol is a derivative of furfural, and its content may also be related to the roasted. The higher scores of samples from GZ in terms of sauce, qu, roasted, and acidic aroma may be related to the content of these compounds (Fig. 1A). Some flavor components with relatively low content and non–volatile compounds, such as ethyl tridecanoate, 3–octanol, and citric acid, are distributed in class 3 (Area V). Most of these flavor compounds show little difference in content among samples from the core production areas. Ethyl tridecanoate has a cola and sesame aroma, while 3–octanol exhibits a fresh green and mushroom–like odor (Table S2). Their average content in samples from the four regions is less than 100 μg/L (Table 1). Citric acid has a relatively high content among the non–volatile organic acids in SAB, and its content may be related to the production process. During the high–temperature stack fermentation process, yeast and other microorganisms have vigorous metabolic and growth activities, which can metabolize and produce citric acid (Zhao et al., 2024).

**Table 1:** Content and distribution range of 87 flavor substances with odor activity value (OAV ≥ 1) in samples from 4 core towns in the Chishui River Basin.

Name	Odor thresholds (μg/L)	Content range(μg/L)				OAV range			
		GZ		SC		GZ		SC	
		MT	XJ	EL	TP	MT	XJ	EL	TP
Ethyl acetate^a^	32551^b^	1205.5–3159.17	2892.9–3599.04	2344.75–2780.25	2195.82–2604.19	37.03–97.05	88.87–110.57	72.03–85.41	67.46–80
Propyl acetate	4740^b^	1617.67–37,396.89	11,513.87–29,079.06	704.65–28,383.65	6229.93–13,204.35	0.34–7.89	2.43–6.13	0.15–5.99	1.31–2.79
3-Methylbutyl acetate	94^b^	2682.12–9850.72	8175.78–9742.8	3136.63–8649.29	3466.67–8102.36	28.53–104.79	86.98–103.65	33.37–92.01	36.88–86.2
Ethyl propanoate	19000^b^	18,940.94–122,993.95	26,553.66–50,387.74	35,397.56–78,572.65	43,369.94–84,622.36	1–6.47	1.4–2.65	1.86–4.14	2.28–4.45
Ethyl 2-methylpropanoate	57.5^b^	2839.41–22,165.74	12,932.52–14,305.05	9897.83–17,759.11	9935.8–18,853.26	49.38–385.49	224.91–248.78	172.14–308.85	172.8–327.88
Ethyl butanoate	81.5^b^	15,304.68–99,880.39	21,781.37–31,941.19	39,967.71–101,407.13	33,554.83–57,100.26	187.79–1225.53	267.26–391.92	490.4–1244.26	411.72–700.62
Butyl butanoate	110^c^	8.45–299.85	40.4–74.16	3.1–806.58	13.21–124.98	0.08–2.73	0.37–0.67	0.03–7.33	0.12–1.14
3-Methylbutyl butanoate	17^c^	0–713.1	0–444.33	7.76–461.65	10.53–412.61	0–41.95	0–26.14	0.46–27.16	0.62–24.27
2-Phenethyl butanoate	961^c^	142.75–1432.31	181.67–339.31	110.09–408.41	177.31–622.35	0.15–1.49	0.19–0.35	0.11–0.42	0.18–0.65
Ethyl 2-methylbutanoate	18^b^	0–8202.43	2759.42–3702.28	4273.44–8749.96	1371.93–9902.37	0–455.69	153.3–205.68	237.41–486.11	76.22–550.13
Ethyl 3-methylbutanoate	7^b^	1636.56–24,229.77	10,357.26–12,645.17	13,172.93–28,321.76	7148.82–30,968.07	233.79–3461.4	1479.61–1806.5	1881.85–4046	1021.26–4424.0
Ethyl pentanoate	26.8^b^	786.05–22,566.24	1341.45–4507.03	2664.86–14,887.85	4731.45–14,393.56	29.33–842.02	50.05–168.17	99.44–555.52	176.55–537.07
Ethyl hexanoate	55.3^b^	25,630.51–67,977.58	30,224.22–37,349.55	37,933.7–123,955.09	44,072.7–84,521.9	463.48–1229.25	546.55–675.4	685.96–2241.5	796.97–1528.42
3-Methylbutyl hexanoate	1400^b^	300.11–5853.41	1624.77–5291.21	234.38–238,793.67	863.59–30,238.93	0.21–4.18	1.16–3.78	0.17–170.57	0.62–21.6
Hexyl hexanoate	1890^d^	110.75–8796.23	345.48–725.82	230.18–11,836.56	2572.43–7327.5	0.06–4.65	0.18–0.38	0.12–6.26	1.36–3.88
2-Phenethyl hexanoate	94^d^	877.42–43,569.56	2106.54–4239.09	971.89–9889.62	8046.06–19,832.55	9.33–463.51	22.41–45.1	10.34–105.21	85.6–210.98
Ethyl octanoate	12.9^b^	571.23–10,456.92	1701.22–2201.93	441.51–2499.97	822.86–2751.19	44.28–810.61	131.88–170.69	34.23–193.8	63.79–213.27
Ethyl decanoate	1122.3^e^	22.06–823.77	26.95–1266.12	3.04–1618.94	86.1–627.2	0.02–0.73	0.02–1.13	0–1.44	0.08–0.56
Ethyl dodecanoate	500^c^	177.15–1153.24	232.62–360.86	229.13–317.15	215.18–332.35	0.35–2.31	0.47–0.72	0.46–0.63	0.43–0.66
Ethyl tetradecanoate	500^f^	547.2–5206.5	959.59–1271.61	62.26–1428.14	860.99–1762.27	1.09–10.41	1.92–2.54	0.12–2.86	1.72–3.52
Ethyl hexadecanoate	1000^b^	22,094.79–42,885.41	19,037.87–26,576.41	26,056.01–42,052.82	22,081.52–32,869.65	22.09–42.89	19.04–26.58	26.06–42.05	22.08–32.87
Ethyl linoleate	4500^b^	8249.11–30,458.17	8384.42–17,229.52	7096.02–15,995.61	8470.75–13,520.54	1.83–6.77	1.86–3.83	1.58–3.55	1.88–3
Ethyl lactate^a^	32600^b^	957.65–2115.68	2089.23–2336.69	1265.56–2292.31	811.26–1338.84	29.38–64.9	64.09–71.68	38.82–70.32	24.89–41.07
Ethyl 2-hydroxy-4-methylpentanoate	1268^d^	4960.73–13,871.55	11,959.64–13,269.31	5159.04–12,357.43	6065.86–9429.54	3.91–10.94	9.43–10.46	4.07–9.75	4.78–7.44
Ethyl benzoate	1430^c^	5597.08–39,940.25	3565.81–9715.45	6126.21–27,184.46	8406.32–19,987.37	3.91–27.93	2.49–6.79	4.28–19.01	5.88–13.98
Ethyl phenylacetate	407^b^	915.06–5420.06	3225.84–3827.89	1160.58–3569.68	2283.39–4026.79	2.25–13.32	7.93–9.41	2.85–8.77	5.61–9.89
Ethyl 3-phenylpropionate	125^c^	17.05–138.98	26.33–132.96	18.02–184.44	24.67–116.83	0.14–1.11	0.21–1.06	0.14–1.48	0.2–0.93
1-Propanol^a^	54000^f^	594.47–6140.35	2003.58–2657.54	500.9–5576.18	1257.71–2851.98	11.01–113.71	37.1–49.21	9.28–103.26	23.29–52.81
1-Butanol^a^	2733^b^	57.08–135.21	68.12–96.96	73.24–125.3	67.05–91.8	20.89–49.47	24.92–35.48	26.8–45.85	24.53–33.59
2-Butanol^a^	50000^b^	22.26–188.20	49.71–84.03	21.66–110.13	25.50–75.84	0.45–3.76	0.99–1.68	0.43–2.2	0.51–1.52
2-Methylpropanol^a^	40000^b^	92.95–197.22	155.74–190.49	111.51–227.83	157.90–253.29	2.32–4.93	3.89–4.76	2.79–5.7	3.95–6.33
1-Pentanol	4000^c^	7925.44–12,812.94	8861.05–9834.35	11,401.45–17,271.23	9870.15–14,925.78	1.98–3.2	2.22–2.46	2.85–4.32	2.47–3.73
3-Methylbutanol^a^	179000^c^	229.37–382.12	275.7–319.6	285.71–478.88	285.67–406.19	1.28–2.13	1.54–1.79	1.6–2.68	1.6–2.27
1-Hexanol	5370^c^	2726.78–15,988.94	4851.88–8605.54	9755.23–32,987.37	9104.88–25,350.55	0.51–2.98	0.9–1.6	1.82–6.14	1.7–4.72
2-Heptanol	1430^c^	1113.54–19,479.58	585.33–3759.43	3467.84–24,208.66	2241.79–11,740.32	0.78–13.62	0.41–2.63	2.43–16.93	1.57–8.21
1-Octanol	1100^b^	15.11–716.57	372.6–642.68	4.37–1693.31	413.83–1021.96	0.01–0.65	0.34–0.58	0–1.54	0.38–0.93
1-Octen-3-ol	6.12^b^	152.63–975.36	2.89–269.13	129.08–1080.01	152.54–259.42	24.94–159.37	0.47–43.98	21.09–176.47	24.92–42.39
1-Nonanol	50^c^	4.73–174.73	38.16–156.04	7.25–127.46	4.57–101.48	0.09–3.49	0.76–3.12	0.15–2.55	0.09–2.03
2-Nonanol	70^c^	3.34–122.75	8.87–21.23	2.39–50.97	1.69–32.48	0.05–1.75	0.13–0.3	0.03–0.73	0.02–0.46
Acetic acid^a^	160000^b^	1428.92–2182.96	1884.18–2262.6	1603.83–1928.67	1429.31–1679.05	8.93–13.64	11.78–14.14	10.02–12.05	0.01–0.01
Propionic acid	18100^b^	40,987.01–107,815.76	46,816.66–60,449.46	52,982.07–76,931.42	52,921.4–78,970.08	2.26–5.96	2.59–3.34	2.93–4.25	2.92–4.36
Butanoic acid	964^b^	18,915.89–39,802.82	26,867.52–29,775.64	23,444.3–28,296.12	25,174.11–29,685.87	19.62–41.29	27.87–30.89	24.32–29.35	26.11–30.79
2-Methylpropanoic acid	1580^b^	23,470.2–70,080.94	25,394.12–31,409.47	38,120.05–66,103.91	34,457.1–49,158.32	14.85–44.36	16.07–19.88	24.13–41.84	21.81–31.11
Pentanoic acid	389^b^	56,697.48–143,127.51	93,371.53–97,924.5	81,355.08–99,505.92	83,359.47–117,136.3	145.75–367.94	240.03–251.73	209.14–255.8	214.29–301.12
3-Methylbutanoic acid	1045^b^	16,771.74–26,520.68	17,349.08–19,045.32	19,109.38–30,004.32	22,975.8–26,543.63	16.05–25.38	16.6–18.23	18.29–28.71	21.99–25.4
Hexanoic acid	2517^b^	13,559.67–44,624.62	16,130.96–21,231.19	21,914.71–76,151.21	25,778.33–52,342.7	5.39–17.73	6.41–8.44	8.71–30.25	10.24–20.8
Octanoic acid	2700^c^	19,280.59–20,636.27	19,716.49–20,046.98	19,881.32–23,107.79	19,643.15–21,687.01	7.14–7.64	7.3–7.42	7.36–8.56	7.28–8.03
Benzoic acid	5000^d^	1171.04–61,922.55	20,560.95–58,289.71	3651.77–78,772.17	18,946.15–83,311.08	0.23–12.38	4.11–11.66	0.73–15.75	3.79–16.66
Acetaldehyde^a^	1200^b^	144.48–955.81	368.89–626.09	552.98–1105.21	497.77–1213.29	0.12–0.8	307.41–521.74	460.82–921.01	414.81–1011.08
2-Methylpropanal	1300^b^	2565.16–10,721.78	8710.26–10,995.65	2922.42–8528.11	6998.02–13,933.98	1.97–8.25	6.7–8.46	2.25–6.56	5.38–10.72
2-Methylbutanal	16^g^	1622.34–9707.62	7904.39–11,701.19	745.5–8077.98	6927.3–11,353.85	101.4–606.73	494.02–731.32	46.59–504.87	432.96–709.62
3-Methylbutanal	16.51^c^	14,048.28–51,313.05	37,127.31–55,547.68	14,388.06–43,567.45	51,514.4–69,276.24	850.9–3108	2248.78–3364.5	871.48–2638.85	3120.19–4196
Nonanal	122^c^	0–587.59	22.76–56.16	26.24–337.58	22.89–88.9	0–4.82	0.19–0.46	0.22–2.77	0.19–0.73
Benzaldehyde	4200^c^	986.09–5128.75	1516.69–2388.75	807.31–2282.86	2811.08–4699.69	0.23–1.22	0.36–0.57	0.19–0.54	0.67–1.12
Benzeneacetaldehyde	262^d^	868.35–43,149.1	11,829.93–49,452.26	9565.53–57,648.65	5861.98–24,047.5	3.31–164.69	45.15–188.75	36.51–220.03	22.37–91.78
Diethoxymethane	24,074.07^h^	721.28–27,434.8	5664.59–7794.78	8460.07–22,396.04	4292.38–32,567.27	0.03–1.14	0.24–0.32	0.35–0.93	0.18–1.35
1,1-Diethoxyethane^a^	2090^c^	91.72–691.31	282.68–440.09	382.67–763.19	384.33–895.72	43.88–330.77	135.26–210.57	183.1–365.16	183.89–428.58
1,1-Diethoxy-3-methylbutane	122.74^d^	8131.89–33,790.2	23,617.64–37,336.76	7384.76–27,714.86	34,141.44–47,000.27	66.25–275.3	192.42–304.19	60.17–225.8	278.16–382.93
Acetone	21,395.22^h^	8958.49–37,408.28	19,984.33–25,656.74	12,963.27–27,924.51	23,835.37–45,417.69	0.42–1.75	0.93–1.2	0.61–1.31	1.11–2.12
2-Pentanone	13806^f^	2827.34–15,118.63	10,570.79–14,211.52	5698.97–24,116.8	12,796.16–27,328.62	0.2–1.1	0.77–1.03	0.41–1.75	0.93–1.98
3-Hydroxyl-2-butanone	259^b^	5060.52–79,428.44	17,274.26–28,038.95	0–11,372.63	15,368.58–36,030.29	19.54–306.67	66.7–108.26	0–43.91	59.34–139.11
2-Heptanone	140^d^	2.98–1787.28	15.59–1098.38	7.01–4814.07	369.66–703.26	0.02–12.77	0.11–7.85	0.05–34.39	2.64–5.02
3-Octanone	70^d^	26.32–184.14	11.07–115.15	24.07–268.49	25.33–65.02	0.38–2.63	0.16–1.65	0.34–3.84	0.36–0.93
Furfuryl alcohol	2000^a^	3764.04–25,727.16	15,814.48–22,527.14	3243.99–15,351.6	4323.87–27,577.95	1.88–12.86	7.91–11.26	1.62–7.68	2.16–13.79
Furfural^a^	44030^b^	27.29–318.54	239.83–308.29	156.68–269.5	302.39–427.63	0.62–7.23	5.45–7	3.56–6.12	6.87–9.71
Furfuryl ethyl ether	30.89^d^	938.98–3380.98	594.24–2160.26	776.62–8378.74	798.45–1859.4	30.4–109.45	19.24–69.93	25.14–271.24	25.85–60.19
HDMF	30.42^i^	23–101.55	52.94–72.41	35.09–88.23	63.96–156.63	0.76–3.34	1.74–2.38	1.15–2.9	2.1–5.15
Sotolon	19^i^	9.36–148.75	13.94–25.79	19.35–79.36	30.06–120.62	0.49–7.83	0.73–1.36	1.02–4.18	1.58–6.35
2,5-Dimethylpyrazine	3032^d^	859.15–5650.24	1298.55–2503.15	1424.79–5578.93	2902.12–5393.51	0.28–1.86	0.43–0.83	0.47–1.84	0.96–1.78
2,6-Dimethylpyrazine	791^e^	159.44–1306.88	157.67–537.57	297.07–547.86	522.73–838.29	0.2–1.65	0.2–0.68	0.38–0.69	0.66–1.06
2-Ethyl-6-methylpyrazine	40^d^	214.38–5511.39	1294.64–2734.37	1777.88–4896.97	2555.7–5269.45	5.36–137.78	32.37–68.36	44.45–122.42	63.89–131.74
2,3,5-Trimethylpyrazine	730^b^	976.37–9604.45	1688.93–2929.31	1170.61–3671.91	3458.01–5351.36	1.34–13.16	2.31–4.01	1.6–5.03	4.74–7.33
2-Ethyl-3,5-dimethylpyrazine	7.5^j^	783.26–12,005.81	895.77–1889.6	1719.52–4105.78	1956.95–6677.2	104.43–1600.77	119.44–251.95	229.27–547.44	260.93–890.29
2-Ethyl-3,6-dimethylpyrazine	8.6^d^	101.57–430.57	71.19–281.97	204.55–582.95	169.71–360.86	11.81–50.07	8.28–32.79	23.78–67.78	19.73–41.96
2,3-Diethylpyrazine	172^d^	0–69.43	14.11–46.43	27.13–78.02	8–875.28	0–0.4	0.08–0.27	0.16–0.45	0.05–5.09
2,3-Diethyl-5-methylpyrazine	18^j^	22.06–126.79	79.97–106	184.22–282.24	3.41–355.99	1.23–7.04	4.44–5.89	10.23–15.68	0.19–19.78
2-Isobutyl-3-methylpyrazine	130^j^	41.22–199.01	78.05–149.92	121.82–191.82	140.01–463.68	0.32–1.53	0.6–1.15	0.94–1.48	1.08–3.57
Dimethyl trisulfide	0.4^b^	29.09–671.68	11.61–205.93	19.06–30.62	145.73–362.45	72.73–1679.2	29.03–514.83	47.65–76.55	364.33–906.13
p-Cresol	167^d^	48.76–1299.94	293.66–446.19	531.17–1971.4	322.2–1585.2	0.29–7.78	1.76–2.67	3.18–11.8	1.93–9.49
Guaiacol	13.41^f^	161.21–4738.87	195.25–391.41	104.57–2238.63	349.72–1198.87	12.02–353.38	14.56–29.19	7.8–166.94	26.08–89.4
4-Methylguaiacol	315^d^	17.18–388.17	51.24–83.27	17.71–60.47	6.75–32.4	0.05–1.23	0.16–0.26	0.06–0.19	0.02–0.1
4-Ethylguaiacol	123^d^	201.59–2236.84	155.27–467.83	178.19–821.25	133.94–373.12	1.64–18.19	1.26–3.8	1.45–6.68	1.09–3.03
4-Vinylguaiacol	209.3^d^	0.81–619.45	1.48–12.72	0.78–9.07	0.83–6.13	0–2.96	0.01–0.06	0–0.04	0–0.03
gams-Nonalactone	90.66^b^	77.51–760.89	227.09–360.64	76.3–266.22	139.03–234.98	0.85–8.39	2.5–3.98	0.84–2.94	1.53–2.59
gama-Dodecalactone	61^c^	323.73–1084.34	323.68–559.79	334.43–628.7	298–410.01	5.31–17.78	5.31–9.18	5.48–10.31	4.89–6.72
beta-Damascone	0.12^f^	1.11–35.2	3.72–14.86	0.5–448.2	1.73–8.53	9.25–293.33	31–123.83	4.17–3735	14.42–71.08
Geranylacetone	60^c^	4.93–83.19	19.13–45.57	0–25.15	10.44–32	0.08–1.39	0.32–0.76	0–0.42	0.17–0.53

Note: ^a^the concentration unit: mg/L; ^b^Represents that the aroma threshold is derived fro m reference (Yi et al., 2022); ^c^Represents that the aroma threshold is derived from reference (Liu & Sun, 2018); ^d^Represents that the aroma threshold is derived from reference (Wang, Tang, et al., 2024); ^e^Represents that the aroma threshold is derived from reference (Fan & Xu, 2011); ^f^Represents that the aroma threshold is derived from reference (Duan et al., 2022); ^g^Represents that the aroma threshold is derived from reference (Wang, Fan, et al., 2020); ^h^Threshold value determined experimentally in this study; ^i^Represents that the aroma threshold is derived from reference (Wang, Gao, et al., 2024); ^j^Represents that the aroma threshold is derived from reference (Yan et al., 2021).

### 3.3 OAV analysis and screening of compounds with regional differences

#### 3.3.1 OAV analysis

The SAB samples from the core production area of the Chishui River basin showed significant differences in aroma characteristics and flavor compound contents. However, the concentration of flavor compounds in baijiu does not fully represent their contribution to aroma. Compounds with an OAV ≥ 1 are generally considered to have a direct contribution to aroma. OAV is the ratio of flavor compound concentration to its corresponding odor threshold, and the odor threshold serves as a bridge connecting concentration with aroma contribution. To our knowledge, the odor thresholds of most flavor compounds identified in this study have been reported (Table 1). This supplementary study determined the thresholds of 15 unreported volatile flavor compounds—ethyl formate, ethyl 5–methylhexanoate, ethyl linolenate, (E)–2–nonen–1–ol, 2–undecanol, (*R*, *R*)–2,3–butanediol, diethoxymethane, acetone, 4–nonanone, (2,2–diethoxyethyl)–benzene, 4–undecanone, 2–tridecanone, phytone, 2–ethylpyrazine, and (trimethylpyrazin–2–yl)methanol—in a 53%vol ethanol solution (Fig. S3 and Table 1). Based on the content and threshold values, the OAV of flavor compounds in 40 samples was calculated (Table 1), and a Venn diagram analysis was conducted for flavor compounds with OAV ≥ 1 (Fig. S4 and Fig. S5). A total of 87 flavor compounds had OAV ≥ 1 (Table 1 and Fig. S4), among which 47 compounds had OAV ≥ 1 in all samples (Table 1 and Fig. S5). 23 flavor compounds, including ethyl acetate, 1,1–diethoxyethane, and 2–ethyl–3,5–diethylpyrazine, had OAV ≥ 10 in all samples (Table 1). Ethyl butanoate, ethyl 3–methylbutanoate, ethyl hexanoate, pentanoic acid, acetaldehyde, 2–methylbutanal, 3–methylbutanal, and 2–ethyl–3,5–dimethylpyrazine—these eight compounds exhibited an OAV ≥ 100 in all samples, highlighting their substantial contribution to the aroma profile of SABs. Fortunately, based on the thresholds determined in this study, it was calculated that dimethoxymethane and acetone had OAV ≥ 1 in multiple samples from both banks of the Chishui River (Table 1), indicating their certain contribution to the aroma of SABs. Dimethoxymethane, a product of the dehydration and condensation of methanol and acetaldehyde, plays a role in alleviating the irritancy of methanol and acetaldehyde in SABs. Acetone, with its sweet fragrance, can promote the volatilization of esters and enhance the richness of the aroma in SABs (Xu et al., 2024).

As shown in Table 1 and Fig. S4, there are six flavor compounds with an OAV ≥ 1 that are exclusively found in MT samples: phenethyl butanoate, ethyl dodecanoate, 2–nonanol, 4–methylguaiacol, 4–vinylguaiacol, and geranylacetone. 4–methylguaiacol, 4–vinylguaiacol, and geranylacetone are associated with sauce, qu, and roasted aroma (Li et al., 2024; Wei et al., 2020), which corresponds to the higher scores of these attributes in MT town. 1–Octanol only showed OAV ≥ 1 in EL samples, where it contributed to the highest fruity aroma with citrus–like aroma. Similarly, 2,3–diethylpyrazine was only detected with OAV ≥ 1 in TP samples, suggesting its potential contribution to the highest roasted in TP town. Hexyl hexanoate, acetone, 2–heptanone, and 2–methylpropyl–3–methylpyrazine demonstrated OAV ≥ 1 in all samples from TP town (Fig. S5), highlighting their substantial contribution to the TP samples. Hexyl hexanoate imparts fruity and sweet aromas to baijiu (He et al., 2022). Acetone can promote the volatilization of the esters (Xu et al., 2024), and 2–heptanone has fruity aromas such as cherry (Table S2). Besides its roasted, 2–isobutyl–3–methylpyrazine also possesses a specific grass aroma (Wu et al., 2023). These compounds have a positive contribution to the grass, roasted, and fruity aroma of TP town. As indicated by Table 1, 3–methylbutyl hexanoate demonstrated an OAV ≥ 1 in all XJ samples. It typically imparts a fruity aroma to baijiu, which may also contribute to the highest grain aroma score observed in XJ town.

#### 3.3.2 OPLS-DA screening of regionally differentiated compounds

**Fig. 3:**
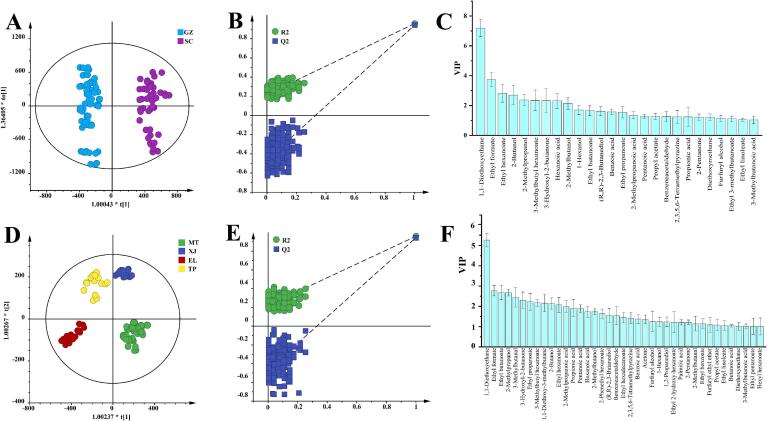
Orthogonal partial least squares-discriminant analysis (OPLS–DA) based on 154 flavor components in SAB samples from the core production area of the Chishui River basin. (A–C) OPLS–DA results for samples from both sides of the Chishui River: (A) score plot, (B) 200 permutation test plot, and (C) variable importance in projection (VIP) plot. (D–F) Corresponding OPLS–DA results for samples from four sub–regions on both sides of the Chishui River: (D) score plot, (E) 200 permutation test plot, and (F) VIP plot.

The core logic of the sampling design in our study lies in the averaging effect. Even if a specific batch of a manufacturer's products deviates from its normal characteristics due to special circumstances, these occasional and non–regional deviations are diluted in the pooled data as we aggregate products from multiple baijiu manufacturers in the same region. Our focus is on identifying compound patterns that can statistically distinguish between different regional sample groups, which are more likely to originate from deep–seated and persistent environmental factors (such as regional climate, soil microbial communities, and brewing microecology) rather than accidental batch and seasonal variations. Based on the content of 154 flavor compounds in 40 samples, OPLS–DA was conducted. The permutation test (*n* = 200) results indicated that both models presented R^2^ > Q^2^, suggesting that the predictive model performed better than the random model. The intersection point of the Q^2^ regression line with the vertical axis was less than zero, indicating no overfitting in the model (Zhang et al., 2024). VIP value can display the contribution of variable differences between samples, and compounds with VIP > 1 are generally considered as key compounds causing differences between groups (Li et al., 2024). As shown in Fig. 3, the OPLS–DA model clearly differentiates samples from different production regions (Fig. 3A and D)—a result that strongly indicates the robustness of the chemical patterns detected at the regional level. The Q^2^ values in Fig. 3B and E were 0.954 and 0.922, respectively, both greater than 0.5, demonstrating that the model fitting results had good predictive ability. From the OPLS–DA models of both sides of the Chishui River and the four regions, 26 (Fig. 3C and Table S7) and 39 flavor compounds (Fig. 3F and Table S8) with VIP > 1 were selected, respectively. Among them, 25 compounds were common regional difference compounds shared by samples from both sides of the Chishui River and the four regions. Ethyl 3–methylbutanoate was the unique compound causing regional differences between SC and GZ samples, while 14 compounds, including ethyl pentanoate, butanoic acid, and 3–methylbutanal, were unique in causing regional differences in samples from the four regions. A total of 40 key compounds causing regional differences among samples within the core production area were screened from two OPLS–DA models. Among them, 1,1–diethoxyethane and ethyl formate are the two compounds with the highest VIP values (Fig. 3C and F), indicating their significant contribution to distinguishing samples from both banks of the Chishui River and among the four regions. It is noteworthy that among the 40 regional difference compounds, 33 have an OAV ≥ 1 (including ethyl hexanoate, pentanoic acid, and 3–methylbutanal). This indicates that these compounds not only contribute to the regional differentiation of samples but also impact the aroma profile of SAB through variations in their concentrations. The other seven compounds—ethyl formate, ethyl 2–hydroxyhexanoate, 2,3,5,6–tetramethylpyrazine, 2–methylbutanol, (*R*, *R*)–2,3–butanediol, 1,2–propanediol, and palmitic acid—all exhibit OAV < 1 in all samples, suggesting that they do not directly contribute to the aroma profile of SABs. Considering the complexity of the aroma in SABs and the potential interactions between flavor compounds, it remains to be further explored whether these seven compounds make potential contributions to the aroma of SABs from different regions.

### 3.4 Screening and validation of key aroma compounds

#### 3.4.1 Correlation network analysis for screening potential key aroma compounds

**Fig. 4:**
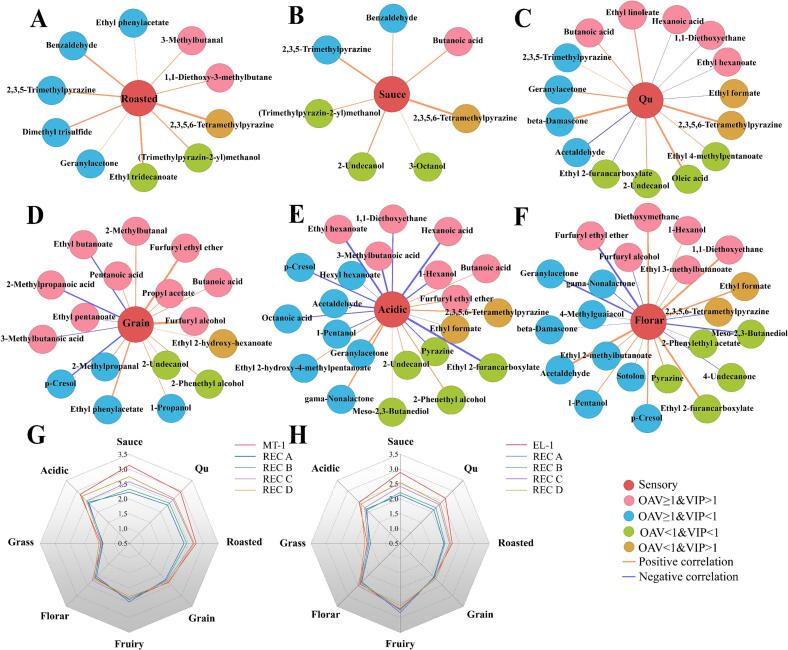
Correlation analysis between flavor compounds and sensory attributes based on Spearman correlation coefficient (|ρ| > 0.5, *P* < 0.05). (A) Roasted aroma, (B) sauce aroma, (C) qu aroma, (D) grain aroma, (E) acidic aroma, and (F) Floral aroma. Sensory radar charts of (G) MT–1 and (H) ET–1 in four different reconstruction schemes.

Network analysis is a powerful tool for exploring potential correlations between flavor compounds and sensory attributes. Spearman correlation coefficients were calculated for 154 flavor compounds and 8 sensory attributes, and nodes and edges with correlation coefficient |ρ| > 0.5 (*P* < 0.05) were selected to construct the correlation network graph (Table S9 and Fig. 4A–4F) (He et al., 2020; Li et al., 2024). Correlation analysis revealed that 52 flavor compounds had correlation coefficients |ρ| > 0.5 (*P* < 0.05) with sensory attributes, including 20 flavor compounds with OAV ≥ 1 and VIP > 1 (pink), 17 compounds with OAV ≥ 1 and VIP < 1 (cyan), 3 compounds with OAV < 1 and VIP > 1 (orange), and 12 aroma compounds with OAV < 1 and VIP < 1 (green). As shown in Fig. 4A–4F, six sensory attributes (roasted (Fig. 4A), sauce (Fig. 4B), qu (Fig. 4C), grain (Fig. 4D), acidic (Fig. 4E), and floral aroma (Fig. 4F)) were significantly correlated with 10, 7, 15,17, 22, and 22 aroma compounds, respectively.

As indicated in Table S9 and Fig. 4A, all ten flavor compounds associated with roasted characteristics exhibited positive correlations. Among these, four compounds—2,3,5–trimethylpyrazine, 2,3,5,6–tetramethylpyrazine, dimethyl trisulfide, and geranylacetone—were consistent with previous findings (Li et al., 2024). Ethyl tridecanoate, 3–methylbutanal, benzaldehyde, and (trimethylpyrazin–2–yl)methanol contribute their monomeric aromas, reminiscent of roasted foods such as nuts, sesame, and cocoa, among others (Table S2). Notably, ethyl tridecanoate and (trimethylpyrazin–2–yl)methanol, due to their low concentrations, may have their nutty and roasted aromas enhanced by other flavor compounds present in the SABs. Correlation analysis also reveals that ethyl phenylacetate, which has a rose–like odor, and 1,1-diethoxy–3–methylbutane, which possesses a fatty odor, both exhibit positive correlations with roasted aromas. Similar to the roasted flavor, seven compounds (butanoic acid, benzaldehyde, 2,3,5–trimethylpyrazine, 2,3,5,6–tetramethylpyrazine, (trimethylpyrazin–2–yl)methanol, 2–undecanol, and 3–octanol) associated with the sauce aroma exhibited significant positive correlations (Fig. 4B). Among these, benzaldehyde, (trimethylpyrazin–2–yl)methanol, 2,3,5,6–tetramethylpyrazine, and 2,3,5–trimethylpyrazine, were concurrently linked to both roasted and sauce aromas, indicating that the origin of the sauce aroma may be connected to the Maillard reaction. Of the remaining three compounds, only butanoic acid has an OAV ≥ 1. Studies have shown that butanoic acid is a key compound related to the sauce aroma in SABs (Gong et al., 2023). The other two higher alcohols, 2–undecanol and 3–octanol, exhibited significant positive correlations with sauce, aligning with the findings of the previous report (Li et al., 2024). They discovered that three higher alcohols—2–methylbutanol, 3–methylbutanol, and nonanol—in SAB also showed significant positive correlations with sauce attributes. Among the 15 compounds associated with qu (Fig. 4C), three (benzaldehyde. 2,3,5–trimethylpyrazine, and 2,3,5,6–tetramethylpyrazine) also exhibit significant positive correlations with roasted and sauce aromas. This correlation may be linked to the “four highs and two longs” production process of SABs. Under high–temperature and high–humidity production conditions, the Maillard reaction intensifies, and compounds with a roasted aroma, such as pyrazine compounds, are produced more readily (Wu et al., 2023; Xiang et al., 2025) and key aroma compounds in soy sauce (Gao et al., 2024) are generated through the Maillard reaction. Research has indicated that intense Maillard reactions are more likely to produce high–quality Daqu, which in turn exhibits superior aroma attributes, including those of sauce, qu, and roasted aromas (Tang et al., 2024). Additionally, ethyl linoleate, butanoic acid, oleic acid, 3–methylbutanal, 1,1–diethoxy–3–methylbutane, geranylacetone, and beta–damascenone exhibited significant positive correlations with qu aroma. Conversely, ethyl formate, ethyl hexanoate, hexanoic acid, acetaldehyde, and acetal demonstrated negative correlations with qu aroma. Among them, 3–methylbutanal, 1,1–diethoxy–3–methylbutane, and geranylacetone are compounds associated with the roasted aroma attribute, whereas butanoic acid is a compound related to the sauce aroma. The correlation among these compounds may be related to the brewing process of SABs. The contribution of oleic acid and ethyl linoleate to the aroma may be associated with microbial metabolic activities during the Daqu–making process. Thermophilic actinomycetes enhance their metabolic activities during the high–temperature Daqu–making process, which facilitates the release of long–chain fatty acids. These microorganisms can also promote ester accumulation through esterase activity, resulting in the formation of corresponding esters (Ma et al., 2025). Both beta–damascenone and geranylacetone belong to terpenes, therefore, their contribution mechanisms to the aroma might be similar. Additionally, five compounds—ethyl hexanoate, ethyl furoate, hexanoic acid, acetaldehyde, and 1,1–dimethoxyethane—exhibit a negative correlation with qu and typically impart fruity and grass aromas to baijiu. These compounds are all low–boiling point compounds, and their volatilization process may suppress the aroma of qu.

Among the 17 compounds associated with grain aroma (Fig. 4D and Table S9), 12 exhibited a significant positive correlation with grain, with furfuryl alcohol demonstrating the highest correlation. Five compounds exhibited a significant negative correlation with grain aroma, with 2–methylpropanoic acid showing the highest correlation. Samples from XJ town had the highest average content of furfuryl alcohol and the lowest average content of 2–methylpropanoic acid (Table S3–S6), corresponding to the highest grain score in XJ town (Fig. 1B). Compounds associated with acidic and floral aromas were the most prevalent, totaling 22 (Fig. 4E and Fig. 4F). Nine of these compounds—including butanoic acid, 2–undecanol, and 2,3,5,6–tetramethylpyrazine—exhibited a significant positive correlation with acidity, as shown in Table S9. Additionally, five compounds—2–undecanol, *meso*–2,3–butanediol, butanoic acid, 2,3,5,6–tetramethylpyrazine, and geranylacetone—were linked to the aromas of sauce and qu, aligning with the findings of Zhang et al. (Zhang et al., 2024). In their study on the empty cup aroma of SAB, they found that certain acids, alcohols, and pyrazines were simultaneously associated with the attributes of acidic, qu, and sauce aroma. The remaining 13 compounds that are negatively correlated with an acidic aroma are primarily esters and alcohols with fruity and floral notes, such as ethyl hexanoate and hexanol. Surprisingly, hexanoic acid, 3–methylbutanoic acid, and octanoic acid also exhibited negative correlations with acidic aroma (Table S9), which may be attributed to their origins and molecular structures. The acids in baijiu are typically produced by microorganisms during fermentation through the metabolism of sugars, proteins, and lipids. The distinct aromas of 3–methylbutanoic acid, hexanoic acid, and octanoic acid have an additional fatty aroma compared to butanoic acid (Table S2). In contrast, butanoic acid exhibits a significant positive correlation with an acidic aroma. This may be due to butanoic acid's shorter carbon chain, which results in more complete lipid metabolism, and its monomeric aroma presents only an acidic note, lacking a fatty aroma. Sixteen compounds demonstrated a positive correlation with floral aroma, including five esters (ethyl formate, phenethyl acetate, ethyl 2–methylbutanoate, ethyl 3–methylbutanoate, and ethyl 2–furancarboxylate) and two alcohols (pentanol and hexanol). Additionally, flavor compounds such as acetaldehyde, diethoxymethane, 1,1–diethoxyethane, sotolon, 4–methylphenol, 4–methylguaiacol, pyrazine, beta–damascone, and gamma–nonalactone also showed significant positive correlations with floral aroma. Studies have indicated that acetaldehyde is a key component of the floral aroma in SAB (Mou et al., 2025), and beta–damascone and 4–methylguaiacol have demonstrated a significant positive correlation with floral aroma in baijiu (He et al., 2020). Six compounds, including *meso*–2,3–butanediol, furfuryl alcohol, 4–undecanone, 2,3,5,6–tetramethylpyrazine, ethyl furfuryl ether, and geranylacetone, have shown a significant negative correlation with floral aroma.

#### 3.4.2 Verification of the aroma contribution of compounds to SABs

Aroma recombination experiments are currently one of the internationally recognized methods for verifying the aroma contribution of flavor compounds (Lin et al., 2024). It is generally believed that volatile flavor compounds with OAV ≥ 1 make direct contributions to the aroma of baijiu and are key aroma compounds in baijiu. Duan et al. (Duan et al., 2024) added volatile flavor components with OAV < 1 to the LSAB recombination model, which showed good reproducibility for the typical soy sauce aroma of LSAB. Previously, it was commonly thought that volatile compounds significantly influenced the flavor of baijiu, whereas non–volatile flavor components frequently received less attention. Recent research has indicated that the inclusion of non–volatile organic acids can improve the aromatic qualities of SAB recombinant samples, enhancing scents such as those of sauce and grain (Lyu et al., 2025). Considering the complexity of SAB and the differences in sensory attributes and flavor compound contents among samples from the core production areas in the Chishui River basin, MT–1 and EL–1 were selected as representative samples from both sides of the Chishui River for aroma recombination experiments, based on baijiu quality and consumer preferences. This study designed four sets of recombination experiments to verify the contributions of flavor compounds with an OAV ≥ 1, twelve volatile compounds with an OAV < 1 selected through correlation analysis for their potential contribution to the aroma of SABs, seven regional difference compounds (with a VIP > 1 and an OAV < 1), and non–volatile organic acids to the aroma of SABs refer to Table S10. The specific schemes are as follows: Recombination A (Rec A) involves adding flavor compounds with an OAV ≥ 1 to a 53%vol ethanol solution. Recombination B (Rec B) entails incorporating 12 aroma compounds, including oleic acid, which have an OAV < 1 and were selected through correlation analysis, into Rec A. Recombination C (Rec C) consists of adding 7 flavor compounds, such as palmitic acid, which have an OAV < 1 and a VIP > 1, to Rec B. Finally, Recombination D (Rec D) involves the addition of 3 non–volatile organic acids—lactic acid, citric acid, and linoleic acid—to Rec C.

QDA analysis was conducted on the reconstructed samples, and the results are presented in Fig. 4G and H. Rec A effectively simulated the grain (MT: 2.23/2.35; EL: 2.10/2.13), fruity (MT: 2.47/2.39; EL: 2.74/2.83), floral (MT: 2.06/2.12; EL: 2.33/2.41), and grass (MT: 1.38/1.46; EL: 1.50/1.61) characteristics of MT–1 and EL–1. Among the flavor components with an OAV of 1 or greater, the majority were esters (Table 1). These esters impart floral and fruity aromas to the SAB, thereby explaining why Rec A can effectively mimic the floral and fruity notes of the original sample. Nonetheless, there remain notable disparities in the typical sauce attributes of SAB, encompassing sauce (MT: 2.23/3.13; EL: 2.13/2.89), qu (MT: 2.33/2.95; EL: 2.10/2.65), roasted (MT: 2.35/2.76; EL: 1.97/2.25), and acidic (MT: 2.46/2.84; EL: 2.13/2.45). The sauce, qu, and roasted aromas are intricate, and their origins are linked to the Maillard reaction. Merely incorporating flavor compounds with an OAV ≥ 1 cannot adequately replicate the corresponding aroma attributes. The shortfall in acidic aroma might stem from the absence of certain potent acidic compounds.

Compared to Rec A, the aroma attributes of sauce (MT: 2.33/2.23; EL: 2.21/2.13), qu (MT: 2.42/2.33; EL: 2.23/2.10), and roasted (MT: 2.44/2.35; EL: 2.06/2.01) increased in Rec B, which verified the contribution of certain flavor compounds with an OAV < 1 to the sauce, qu, and roasted aroma, as shown in Fig. 4A–4C. The fruity aroma in Rec B samples decreased slightly (MT: 2.40/2.47; EL: 2.72/2.83), possibly due to the addition of oleic acid in Rec B. Studies have shown that oleic acid can inhibit the volatilization of ethyl acetate and ethyl hexanoate, thereby affecting their aroma characteristics (Q. Liu et al., 2024). Furthermore, we observed that the decline in EL–1 was greater than that in MT–1, which may be related to the content of ethyl hexanoate in the samples. The content of ethyl hexanoate in EL–1 is approximately three times that of MT–1 (Table S3 and Table S5). Ethyl acetate and ethyl hexanoate also contribute to the floral aroma of baijiu. However, the floral aroma scores of the two Rec B samples increased (MT: 2.06/2.14; EL: 2.33/2.36), possibly due to the addition of pyrazine and ethyl 2–furancarboxylate, which showed a significant positive correlation with floral aroma.

After incorporating seven regional difference compounds into Rec C, based on Rec B, significant improvements were observed in the scores for sauce (MT: 2.58/2.33; EL: 2.42/2.21), qu (MT: 2.59/2.42; EL: 2.35/2.23), roasted (MT: 2.66/2.44; EL: 2.16/2.06), acidic (MT: 2.53/2.41; EL: 2.19/2.08), and floral (MT: 2.26/2.09; EL: 2.56/2.36). These enhancements also confirmed the contribution of these flavor components to the aroma of SABs. Additionally, MT–1 and EL–1 demonstrated differences in their ability to enhance these aroma characteristics. MT–1 showed greater enhancement in the aroma of sauce, qu, roasted, and acidic flavors compared to EL–1, whereas EL–1 exhibited a more pronounced enhancement in floral aroma than MT–1. The findings suggest that the seven regional differences not only contribute to the aroma of SAB but also vary in their levels of contribution depending on the region. Notably, 2,3,5,6–tetramethylpyrazine correlates with five aroma characteristics, exhibiting positive correlations with sauce, qu, roasted, and acidic aromas, and a negative correlation with floral (Fig. 4F). The concentration of 2,3,5,6–tetramethylpyrazine in MT–1 was higher than in EL–1 (Table S3 and Table S5), aligning with the differing intensities of these five aroma characteristics between MT–1 and EL–1. Additionally, we observed that the intensities of sauce and roasted aromas were greater than those of acidic and qu, which may be related to their compositions. Among these seven regional differential compounds, there are high–boiling point acids (such as palmitic acid with a boiling point of 351 °C) and low–boiling point esters (like ethyl formate with a boiling point of 54.7 °C. Their composite aroma may be related to the improvement of sauce scores. The enhancement of the roasted aroma might also be associated with (*R*, *R*)–2,3–butanediol, an important precursor of acetoin. Acetoin can generate pyrazine compounds with a roasted aroma through the Maillard reaction (Shi et al., 2022). In Rec C, the enhancement of the acid aroma in MT–1 was more significant than that in EL–1, possibly due to the higher palmitic acid content in MT–1 (Table S3 and Table S5).

After adding lactic acid, citric acid, and linoleic acid, the sauce attributes of Rec D (MT: 2.75/2.58; EL: 2.55/2.42), acidic (MT: 2.75/2.53; EL: 2.37/2.19), and grass (MT: 1.52/1.43; EL: 1.69/1.58) were significantly enhanced compared to Rec C. This enhancement might be attributed to the presence of numerous low–boiling point esters among the aroma compounds with an OAV ≥ 1. By incorporating additional high–boiling point acids, more complex aroma combinations could be formed, thereby promoting the expression of sauce attributes. Lactic acid and acetic acid are the two most prevalent acidic compounds in SAB (Lyu et al., 2025), with similar concentrations (Table S3–S6). Given that lactic acid is more potent than acetic acid, the intensity of the acidic aroma in Rec C increased notably. Chen et al. (Chen et al., 2022) reported that linoleic acid can produce hexanal, a compound with a grass flavor, *via* enzymatic oxidative cleavage. The linoleic acid content in samples from the left bank of the Chishui River was found to be higher than that from the right bank (Table 1), correlating with the elevated grass aroma scores in samples from the left bank depicted in Fig. 1A. Furthermore, we found that the fruity attribute in Rec D decreased further, linoleic acid may also inhibit the volatilization of ethyl acetate and ethyl hexanoate.

Although the four recombination experiments still exhibit certain discrepancies compared to the original samples in terms of sauce (MT: 2.75/3.13; EL: 2.55/2.89) and qu (MT: 2.64/2.95; EL: 2.41/2.65), it can be confirmed that volatile compounds with an OAV < 1, particularly regional difference compounds (OAV < 1 and VIP > 1), as well as non–volatile organic acids, contribute to the aroma of SABs. These compounds not only enhance the sauce and roasted flavor of SABs but also help to converge the fruity flavor.

### 3.5 Screening key regional markers

**Fig. 5:**
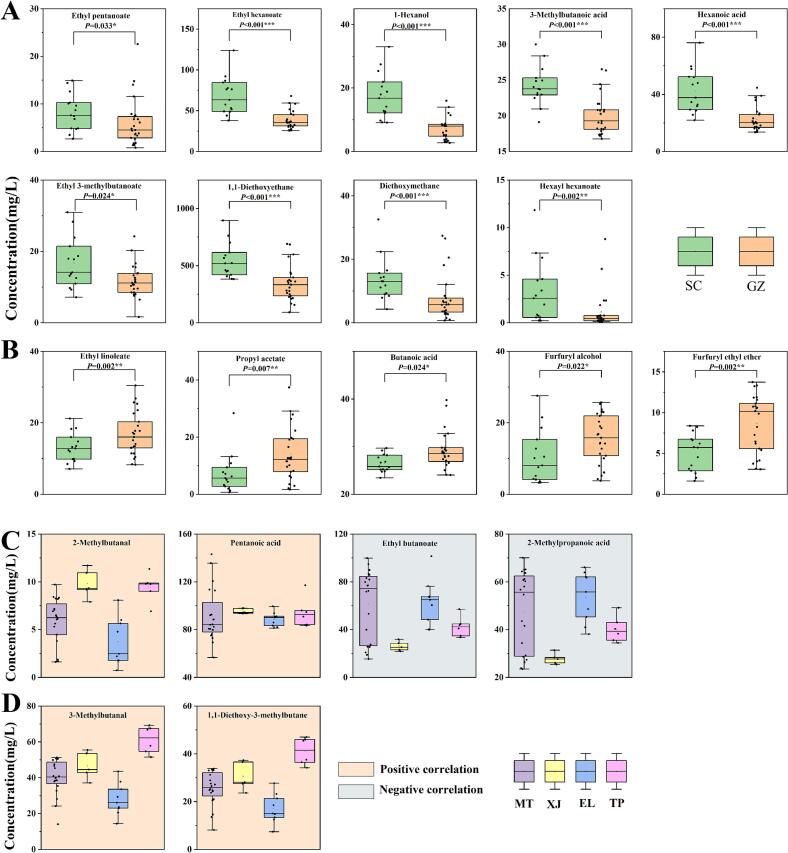
Distribution of key regional markers in the core production area of the Chishui River basin. (A) Content distribution of the 9 key regional markers contributing to floral, fruity, and grass aromas on both sides of the Chishui River; (B) Content distribution of five key regional markers contributing to the sauce, qu, roasted, and grain scent on both sides of the Chishui River; (C) Content distribution of four key regional markers contributing to grain aroma in four regions; (D) Content distribution of two key regional markers contributing to the roasted aroma in four regions. Significance was calculated using the Mann-Whitney test, with *P* < 0.05 considered significant.*: *P* < 0.05, **: *P* < 0.01, ***: *P* < 0.001, NS: not significant.

Considering the significant differences in sensory attributes and flavor compound contents among samples from the core production area of SABs, flavor compounds with OAV ≥ 1 and |ρ| > 0.5 (*P* < 0.05) were selected from 40 regionally differentiated compounds (VIP > 1) as key regional markers for the core production area of the Chishui River basin. As shown in Fig. S6, this study identified 20 key regional aroma markers, which exhibited significant differences in content distribution between the left and right banks of the Chishui River and across various sub–regions. This difference is dominated by the metabolic activities of brewing microorganisms, while the microbial community structure and metabolic functions are regulated by the long–term synergistic effects of the terroir conditions (geographical factors, climatic factors) in the Chishui River basin, brewing raw materials, and traditional brewing techniques. Zhou et al. (Zhou et al., 2024) demonstrated in their study that the distribution of brewing microbial communities in the Chishui River basin is significantly correlated with environmental factors such as temperature, atmosphere, and altitude. Notably, the abundance of *Bacillus* and *Oceanobacillus* species showed a positive correlation with altitude. The low temperature and hypoxic environment in high–altitude areas is more conducive to the enrichment of anaerobic or facultative anaerobic bacterial communities, including *Clostridium*, *Bacillus*, *Oceanobacillus*, and *Lactiplantibacillus plantarum* (*L. plantarum*). In contrast, the high–temperature and high–humidity environment in low–altitude areas favors the enrichment and metabolism of thermotolerant microorganisms (such as *Bacillus licheniformis* and *thermophilic actinomycetes*). Additionally, the low–altitude production areas in the Chishui River basin exhibit richer functional fungal communities associated with brewing (Yuan et al., 2025). The elevation of the SAB production areas on the left bank of the Chishui River (approximately 450 m in EL Town and 1100 m in TP Town) is generally higher than that on the right bank (approximately 400 m in MT Town and XJ Town). Based on this, it is inferred that the left bank environment is more conducive to the enrichment of microbial communities such as *Clostridium*, *Bacillus*, and *L*. *plantarum*. Previous studies have confirmed that the synthesis of key short–chain fatty acids such as hexanoic acid and 3–methylbutanoic acid in SAB is closely associated with *L*. *plantarum* (Yuan et al., 2025). Additionally, *Clostridium* and *Bacillus* can generate short–chain fatty acids like hexanoic acid and butanoic acid through fatty acid oxidation pathways (Qiu et al., 2024; Ren et al., 2025). Notably, *Bacillus* strains not only synthesize hexanoic acid and butanoic acid but also catalyze their esterification reactions with alcohols to produce ester compounds such as ethyl hexanoate and ethyl butanoate (Wen et al., 2025). The microbial metabolic mechanisms described above exhibit strong correlation with the distribution patterns of regional markers in this study: 9 characteristic floral and fruity aroma compounds—including 3–methylbutanoic acid, hexanoic acid, ethyl 3–methylbutanoate, ethyl hexanoate, and hexyl hexanoate—showed higher abundance on the left bank of the Chishui River (Fig. 5A). This finding corroborates the significant differences in sensory scores for floral and fruity aromas observed between samples from both banks in Fig. 1A.

The high–temperature and high–humidity climatic conditions are conducive to the synthesis and accumulation of starch (particularly amylopectin) in sorghum (Ke et al., 2025). The relatively high amylopectin content in sorghum makes it more prone to water absorption and swelling during cooking, leading to complete gelatinization and subsequent easier degradation and utilization by brewing microorganisms (Zahid et al., 2025). Meanwhile, high–temperature and high–humidity environments can promote the Maillard reaction during Daqu–making and pile fermentation, thereby enhancing the formation of characteristic flavor compounds such as sauce aroma and qu aroma in the SAB (Tang et al., 2024). The aforementioned environmental and process effects are consistent with the findings of this study, namely that the abundance of five characteristic compounds associated with sauce and qu aroma (propyl acetate, ethyl linoleate, butanoic acid, furfuryl alcohol, and ethyl furfuryl ether) was significantly higher on the right bank compared to the left bank (Fig. 5B). The enrichment of these characteristic compounds may be closely related to the more diverse fungal community structure in this region. As a fungal group with prominent functions and diversity in the brewing system, *Saccharomyces cerevisiae* not only synthesizes large amounts of ethanol but also produces important flavor compounds such as butanoic acid and furfural (Wang, 2022). A seemingly contradictory phenomenon was observed in this study. According to the above analysis, bacterial groups such as *Bacillus* were more abundant on the left bank of the Chishui River, which theoretically should result in a higher butanoic acid content. Meanwhile, fungi such as *Saccharomyces cerevisiae* were more abundant on the right bank, which also favors the production of butanoic acid. The measured results of this study showed that butanoic acid content was higher on the right bank, suggesting that fungi may contribute more than bacteria to the formation of flavor compounds in SAB. This result is consistent with the conclusion of Liu et al. (Liu et al., 2023), who reported that fungi in the core production regions play a more prominent role during the fermentation of SAB. Furfuryl alcohol is a derivative of furfural. In SAB, its content was significantly negatively correlated with the abundance of *Oceanobacillus* (Xu et al., 2023). In addition, *thermophilic actinomycetes* can participate in the formation of furfuryl alcohol (Yang et al., 2025), while the production of ethyl linoleate is closely related to *Mucor* among fungi (Han et al., 2012). The above microbial metabolic pathways are highly consistent with the distribution characteristics of regional markers in this study.

The remaining six markers (ethyl butanoate, 2–methylpropanoic acid, pentanoic acid, 2–methylbutanal, 3–methylbutanal, and 1,1–diethoxy–3-methylbutane) did not show significant differences between the two banks but displayed distinct abundance patterns across the four sub–regions (MT, XJ, EL, and TP town) (Fig. 5C and D). These compounds are associated with grain and roasted aromas (Fig. 4C and D). Notably, the content distribution of ethyl butanoate and 2–methylpropanoic acid (negatively correlated with grain aroma) was inversely related to grain aroma scores across regions, whereas pentanoic acid and 2–methylbutanal (positively correlated with grain aroma) showed concordant trends with sensory scores (Fig. 5C). In Fig. 5D, except for MT town, the regional abundance of 3–methylbutanal and 1,1–diethoxy–3–methylbutane still largely aligned with roasted aroma intensity in three of the four regions. The reason for the abnormal data of MT town may be related to 2,3,5,6–tetramethylpyrazine, which has the strongest correlation with roasted attributes (Table S9), while its content in MT town samples is the highest among the four towns (Table S3–S6). Moreover, although the content of 3–methylbutanal and 1,1–diethoxy–3–methylbutane in MT town samples ranks third among the four towns, their roasted aroma attributes are second only to TP town. These 20 key markers effectively capture the regional aroma specificity of SABs and provide a chemical basis for the observed sensory differences between production areas. The key regional biomarkers identified in this study provide clear chemical clues and verification targets for the above scientific hypotheses. Future integrated correlation analysis combining metagenomics, metabolomics, and flavoromics will further reveal the microbial metabolic pathways underlying the formation of these biomarkers, thereby enabling a deeper elucidation of the terroir characteristics of SAB at the molecular mechanism level.

The methodology employed in this study incorporates widely adopted techniques from existing research, including detection methods, threshold determination, sampling methods, and sensory reconstitution. Nevertheless, we acknowledge that these approaches still present certain limitations. (a) Inherent limitations in selectivity and coverage of multi–technology combination approaches: this study integrated multiple techniques including DI, HS–SPME, LLE, GC–FID, GC–MS, HPLC, and UHPLC–MS/MS. While these methods maximized compound detection throughput, they introduced inherent methodological biases such as competitive adsorption in SPME, sample loss in LLE, sensitivity constraints in DI, and detection blind spots for thermally unstable or strongly polar compounds. Additionally, co–elution issues in chromatography may interfere with accurate quantification. Therefore, our results represent the best available information under current technical conditions but do not exhaust all chemical constituents of SAB. (b) Matrix effects in threshold determination: although the threshold was measured in a 53%vol ethanol solution to simulate real matrices, the complex interactions among hundreds of trace components in baijiu may still affect the accuracy of OAV calculations. (c) Limitations of sample sources: although we strive to control batch variations, the use of commercial samples implies that specific blending practices of individual manufacturers may introduce certain style deviations that cannot be entirely attributed to geographical factors. (d) Limitations of the aroma reconstruction model: although we conducted reconstruction experiments by sequentially adding OAV ≥ 1, OAV < 1, and non–volatile compounds, subtle sensory differences remained between reconstructed samples and original samples, indicating that complex interactions (e.g., synergistic/antagonistic effects) among all matrix components in authentic liquor bodies have not been fully replicated. Future studies should employ a broader range of original liquor samples, advanced detection technologies, and data analysis methods to conduct multidimensional validation by integrating factors such as brewing processes, microbial metabolism, and geographical influences.

## 4 Conclusion

This study systematically investigated the key aroma compounds and regional markers of SAB from the core production area of the Chishui River basin by integrating multi–detection technologies, molecular sensory science, and chemometrics. Significant regional aroma differences were identified: samples from the right bank (GZ) exhibited stronger sauce, qu, roasted, grain, and acidic aromas, whereas those from the left bank (SC) displayed more intense fruity, floral, and grass notes. A total of 154 aroma compounds were identified and quantified, among which 87 had OAV ≥ 1—including 47 present in all samples, 23 with OAV ≥ 10, and 8 with OAV ≥ 100—underscoring their significant contribution to the aroma profile of SAB. The OPLS–DA model effectively distinguished samples from different regions, based on a VIP > 1, 40 regional difference compounds were screened. Spearman correlation analysis (|ρ| > 0.5, *P* < 0.05) further revealed potential relationships between 52 flavor compounds and 6 sensory attributes. Recombination experiments confirmed that compounds with OAV ≥ 1, certain low–odor compounds (OAV < 1), and non–volatile organic acids collectively shape the overall aroma of SAB. From these 40 regional differential compounds, 20 key regional markers were further identified using OAV ≥ 1 and correlation analysis. These markers not only provide a chemical explanation for the sensory differences of SAB in the core production areas but also hold potential for practical application as identification tools. For instance, a standardized quantitative detection panel targeting the key regional markers can be established based on the validated UHPLC–MS/MS and GC–MS methods in this study. This panel covers representative compounds including esters, acids, pyrazines, and aldehydes, utilizing a combination of multiple reaction monitoring (MRM) and selected ion monitoring (SIM) modes to ensure accurate quantification with high sensitivity and specificity in complex matrices. Subsequently, a comprehensive chemical fingerprint database can be constructed, covering the core production regions (Such as Maotai Town, Xijiu Town, Erlang Town, and Taiping Town). The database would integrate concentration data of the markers from different years, batches, and manufacturers, forming a regionally representative benchmark dataset. By incorporating machine learning algorithms (such as random forest, support vector machine, or partial least squares discriminant analysis), discriminative models for geographical origin authentication can be developed, enabling rapid identification and verification of unknown samples. At the enterprise level, this detection panel can serve as an internal quality control tool to monitor batch–to–batch flavor consistency and the stability of regionally characteristic profiles. Furthermore, the established analytical methods and marker panels demonstrate strong transferability and can be extended to the origin traceability and authenticity protection of other types of baijiu and fermented foods, thereby providing technical support for the quality supervision of geographically indicated food products.

Additionally, the quantitative methods employed in this study were rigorously validated for linearity, recovery rate, and matrix effect compensation, with the obtained compound concentrations consistent with those reported in multiple independent studies on SAB from different production regions. This consistency indicates that the data reflect the relatively stable chemical characteristics of SAB rather than random or sample–specific variations. Furthermore, the integrated research paradigm adopted in this study—combining sensory analysis, OAV calculation, chemometrics (OPLS–DA, correlation networks), recombination experiments, and biomarker screening—is a well–established molecular sensory science methodology. This approach is not only applicable to SAB but can also be extended to other aroma types of baijiu and even to research on the regional characteristics or authenticity of other food and beverages, thereby highlighting its strong methodological transferability and practical application potential.

## CRediT authorship contribution statement

**Long Ma:** Writing – original draft, Investigation, Formal analysis, Data curation, Conceptualization. **Tingyao Tu:** Validation, Methodology, Data curation, Conceptualization. **Mansi Niu:** Validation, Methodology, Formal analysis. **Yuqin Tong:** Software, Methodology. **Xingrong Zhao:** Formal analysis. **Junjie Jia:** Writing – review & editing, Supervision, Funding acquisition, Conceptualization. **Lei Zheng:** Supervision, Methodology. **Rongkun Tu:** Supervision, Resources. **Songtao Wang:** Supervision, Project administration, Funding acquisition. **Suyi Zhang:** Supervision, Resources. **Caihong Shen:** Supervision, Resources.

## Ethical statement

The sensory analysis in this study was conducted by a trained evaluation team who performed sensory evaluations without any invasive procedures or collection of personal data beyond the scope of sensory responses. All members of the evaluation team voluntarily participated after understanding the purpose and process of the study, and participants provided written informed consent before participating in the study. Therefore, ethical permission was not required for this work.

## Declaration of competing interest

The authors declare that they have no known competing financial interests or personal relationships that could have appeared to influence the work reported in this paper.

## Data Availability

Data will be made available on request.
