# Supplementary material for: Revealing regional markers of sauce aroma baijiu in the Chishui River basin: Integrating multi-detection technologies with molecular sensory science and chemometrics
Source: Food Chem X. 2026 Apr 3;35:103821. doi: 10.1016/j.fochx.2026.103821 (PMC13091158; doi:10.1016/j.fochx.2026.103821)
Supplement: Supplementary file 1 — Supplementary material [file mmc1.docx]

**Supporting Information**

**Revealing Regional Markers of Sauce Aroma Baijiu in the Chishui River Basin: Combining Multi-Detection Technologies with Molecular Sensory Science and Chemometrics**

Long Ma^a,b,c,†^, Tingyao Tu^a,b,c,†^, Mansi Niu^a,b,c^, Yuqin Tong^a,b,c^, Xingrong Zhao^a,b,c^, Junjie Jia^a,b,c^,* Lei Zheng^b,c^, Rongkun Tu^b,c^, Songtao Wang^a,b,c^*, Suyi Zhang^b,c^, Caihong Shen^b,c^

^a^Luzhou Pinchuang Technology Co. Ltd., Luzhou 646000, China

^b^National Engineering Research Center of Solid-State Brewing, Luzhou 646000, China

^c^Luzhou Laojiao Co. Ltd., Luzhou 646000, China

^†^These authors contributed to this work equally and should be regarded as co-first authors.

***Corresponding Author:**

E-mail: * Corresponding authors at: Luzhou Pinchuang Technology Co. Ltd., National Engineering Research Center of Solid-State Brewing, Luzhou 646000, China (J. Jia; S. Wang).

E-mail addresses: jiajunjie1010@126.com (J. Jia), lzpckj@126.com (S. Wang)


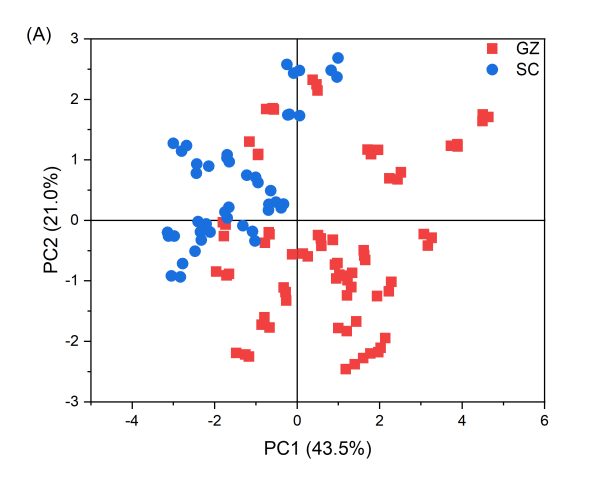

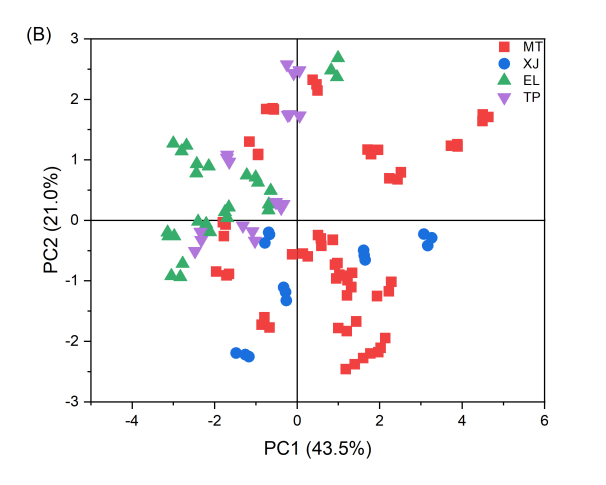


Fig. S1 Principal component analysis (PCA) score plot of quantitative descriptive analysis (QDA) results for sauce aroma baijiu (SAB) samples from the core production area of the Chishui River Basin. (A) PCA scores of samples from both sides of the Chishui River; (B) PCA scores of samples from four regions of the Chishui River.


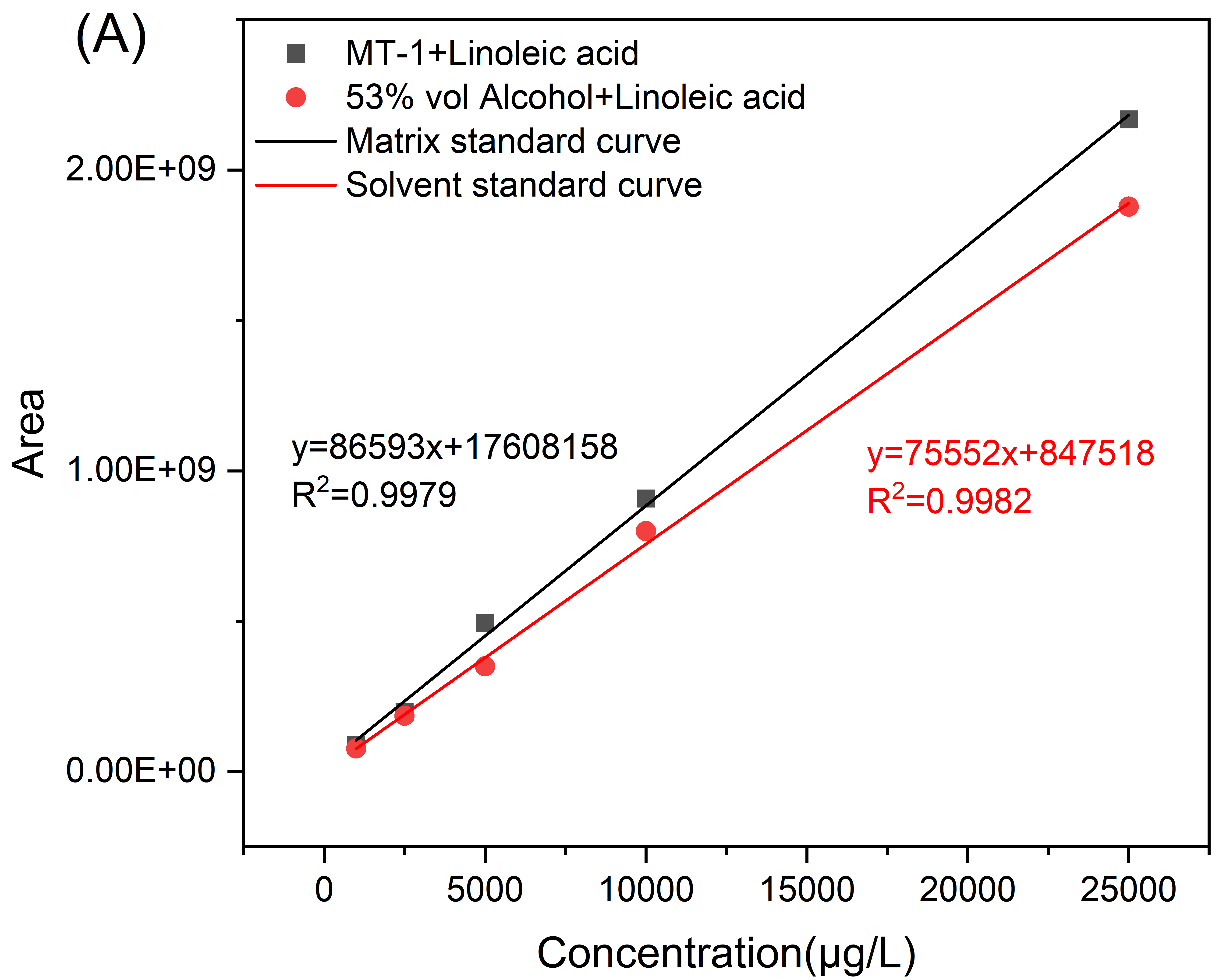

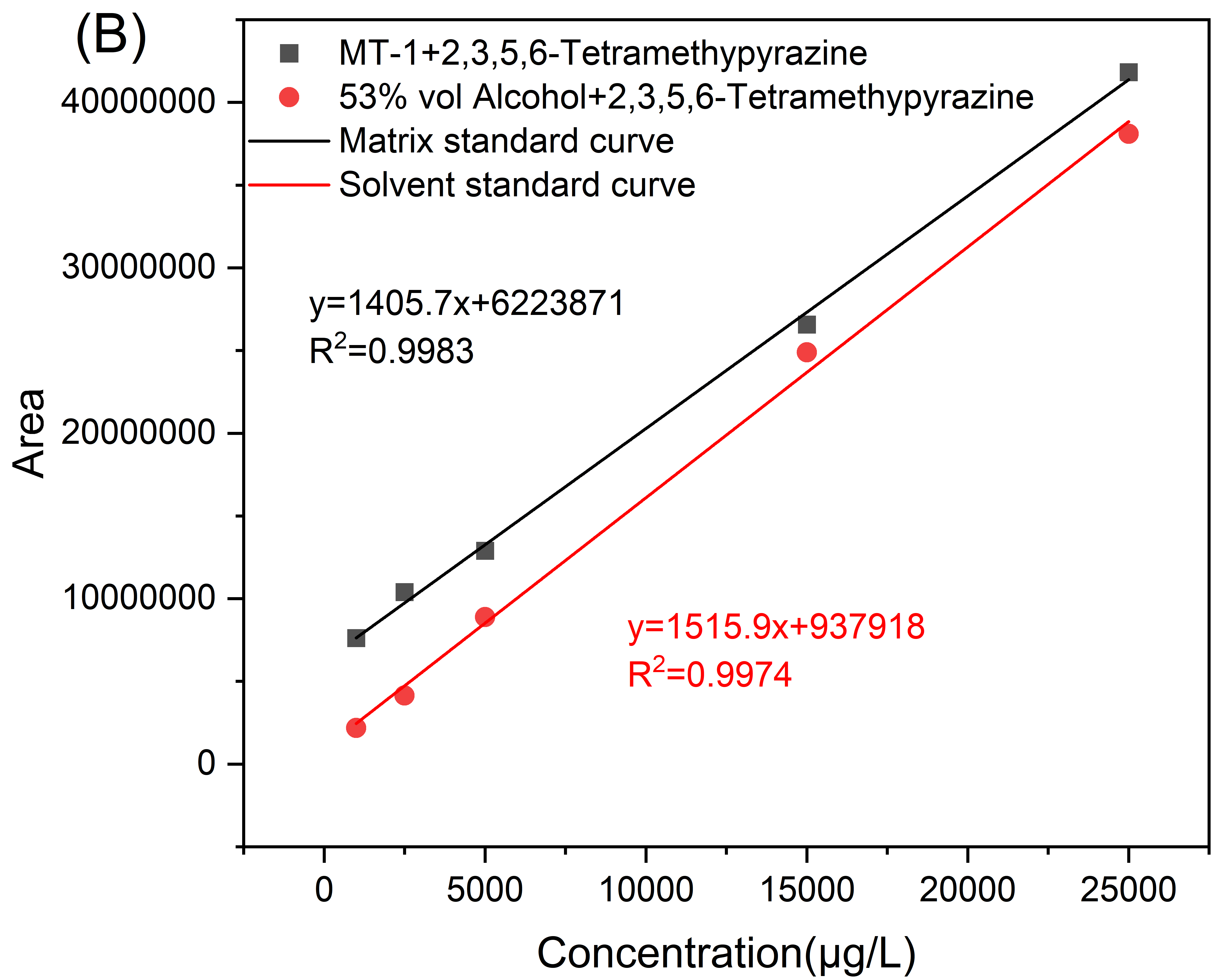


Fig. S2 Matrix standard curves for (A) linoleic acid and (B) 2,3,5,6-tetramethylpyrazine.


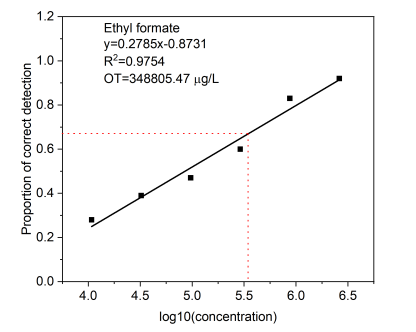

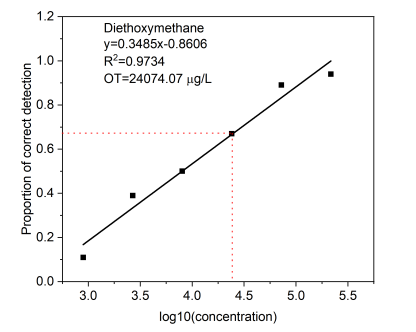

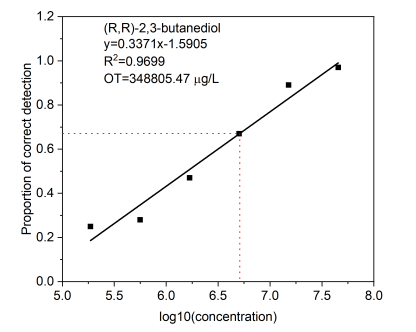

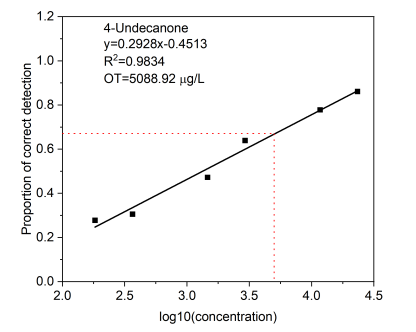

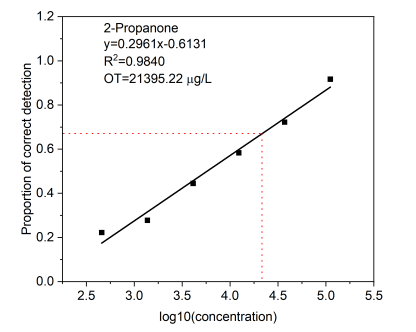

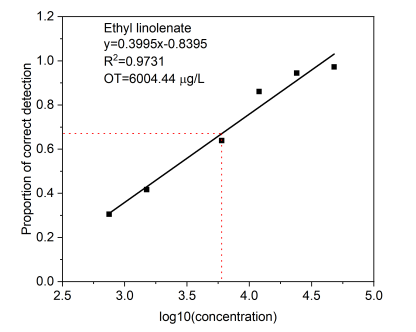

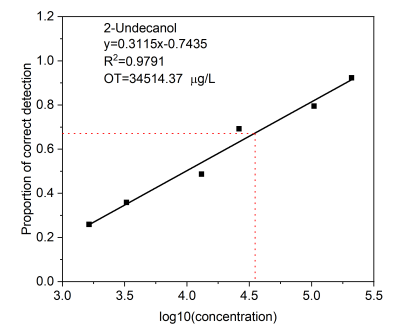

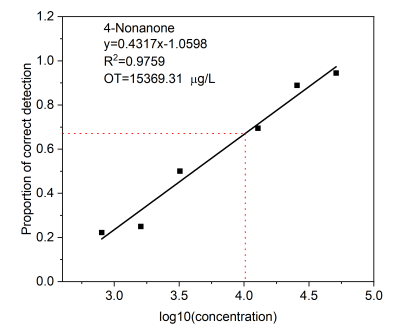

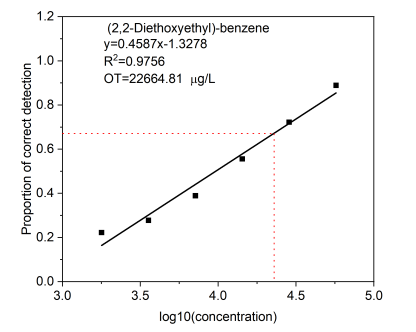

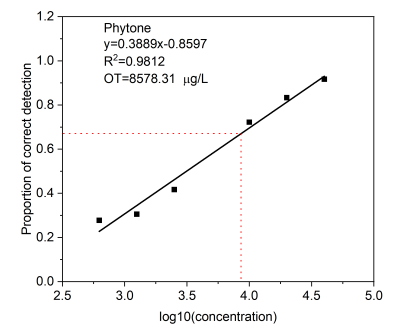

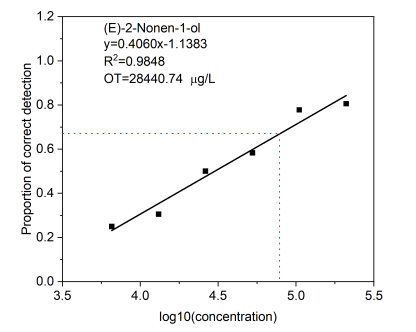

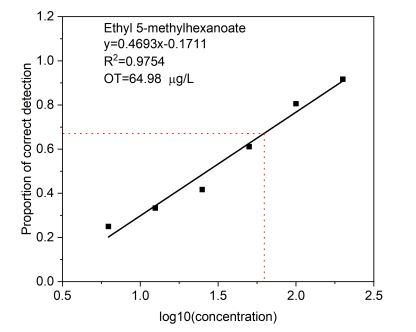

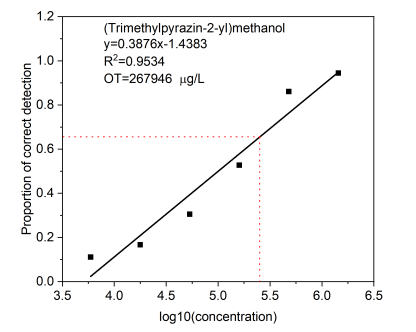

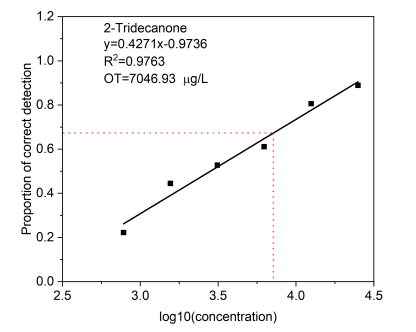

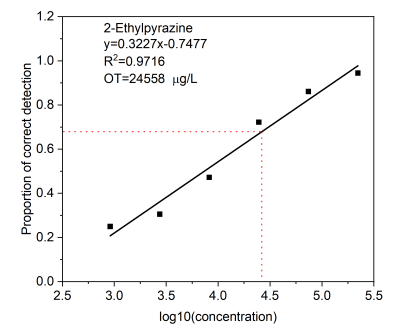
Fig. S3 Threshold fitting curves of the 15 flavor compounds determined in this study.


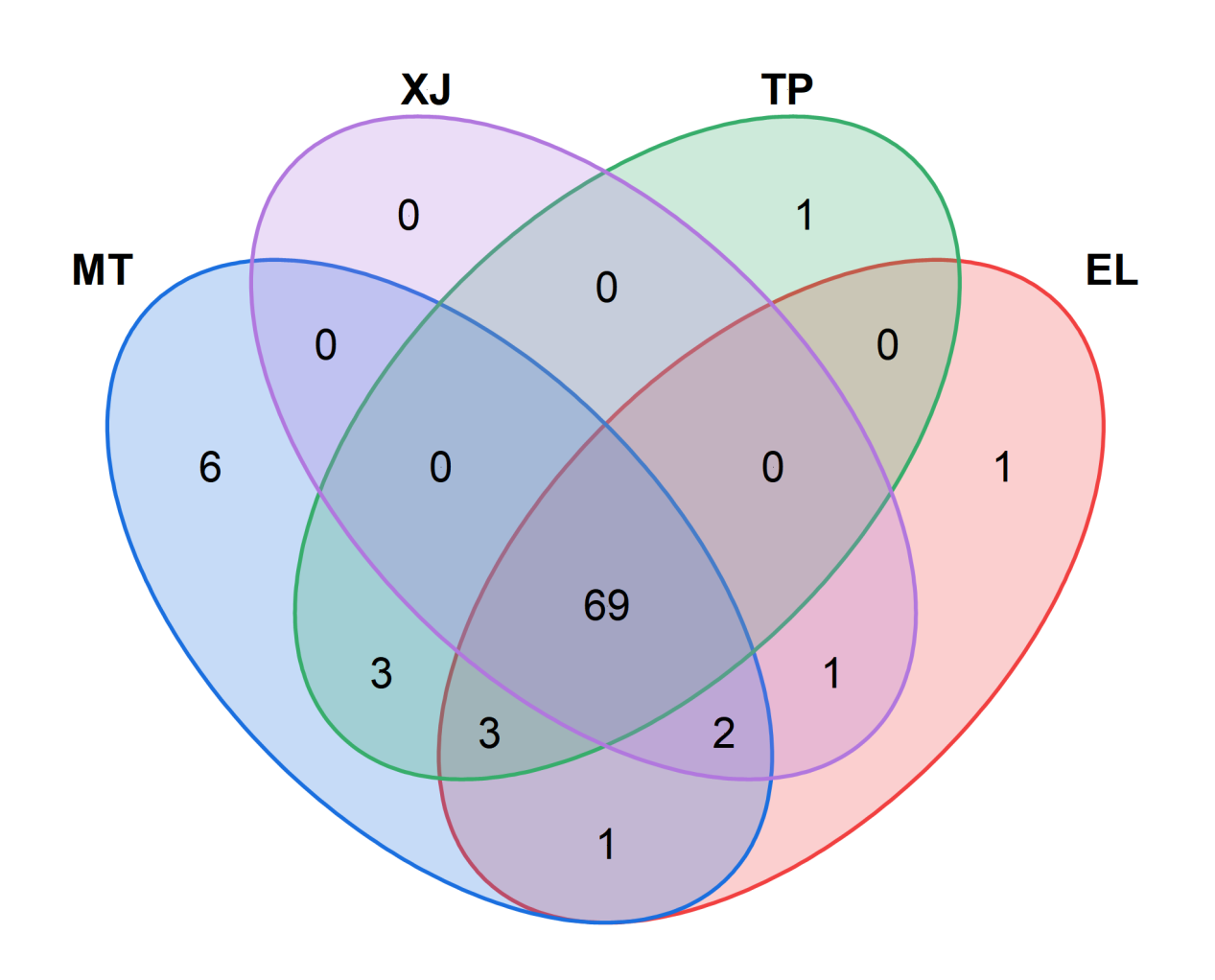


Fig. S4 Distribution of flavor compounds with odor activity value (OAV ≥ 1) in four regions.


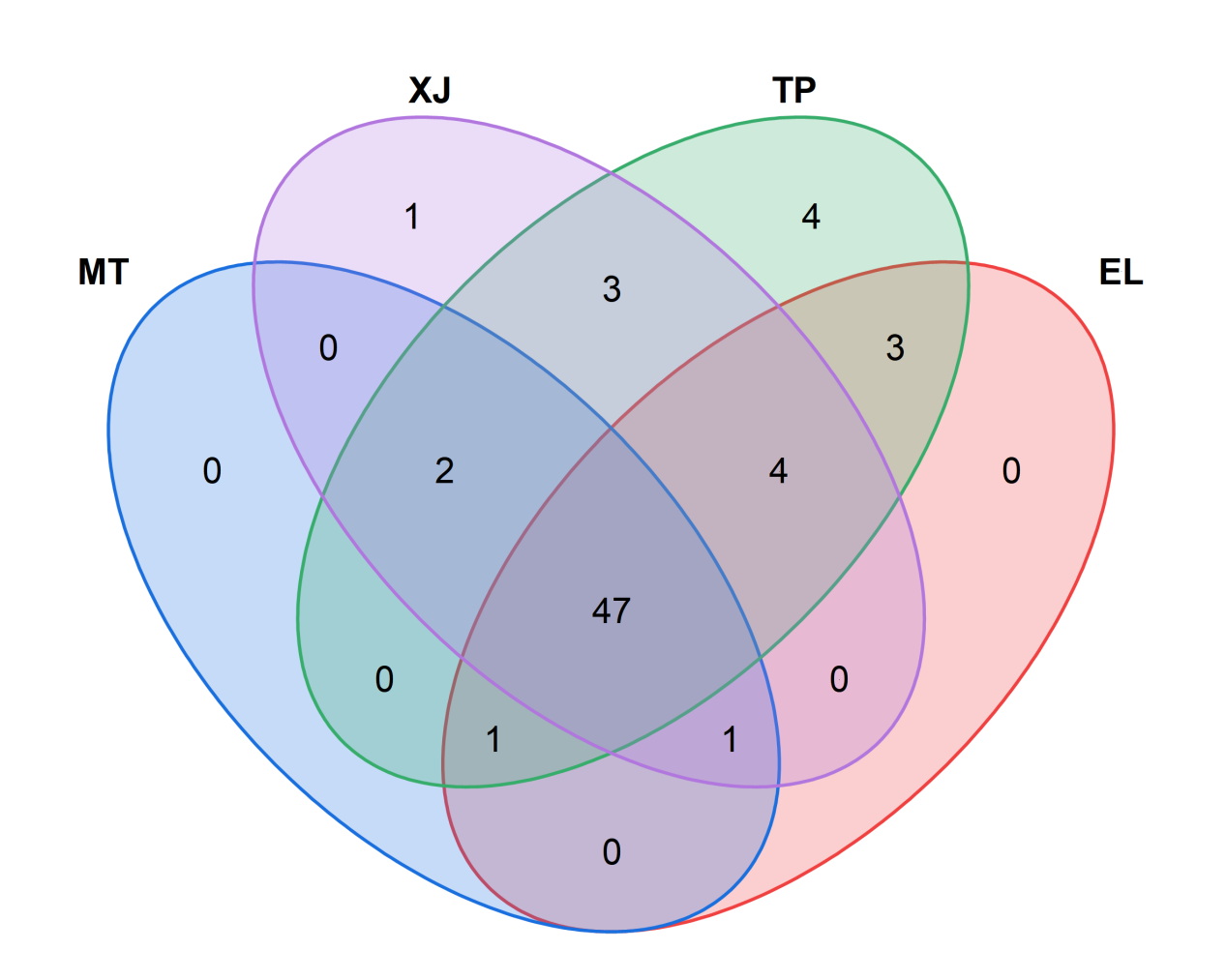
Fig. S5 Distribution of flavor compounds with OAV ≥ 1 in all samples across four regions.


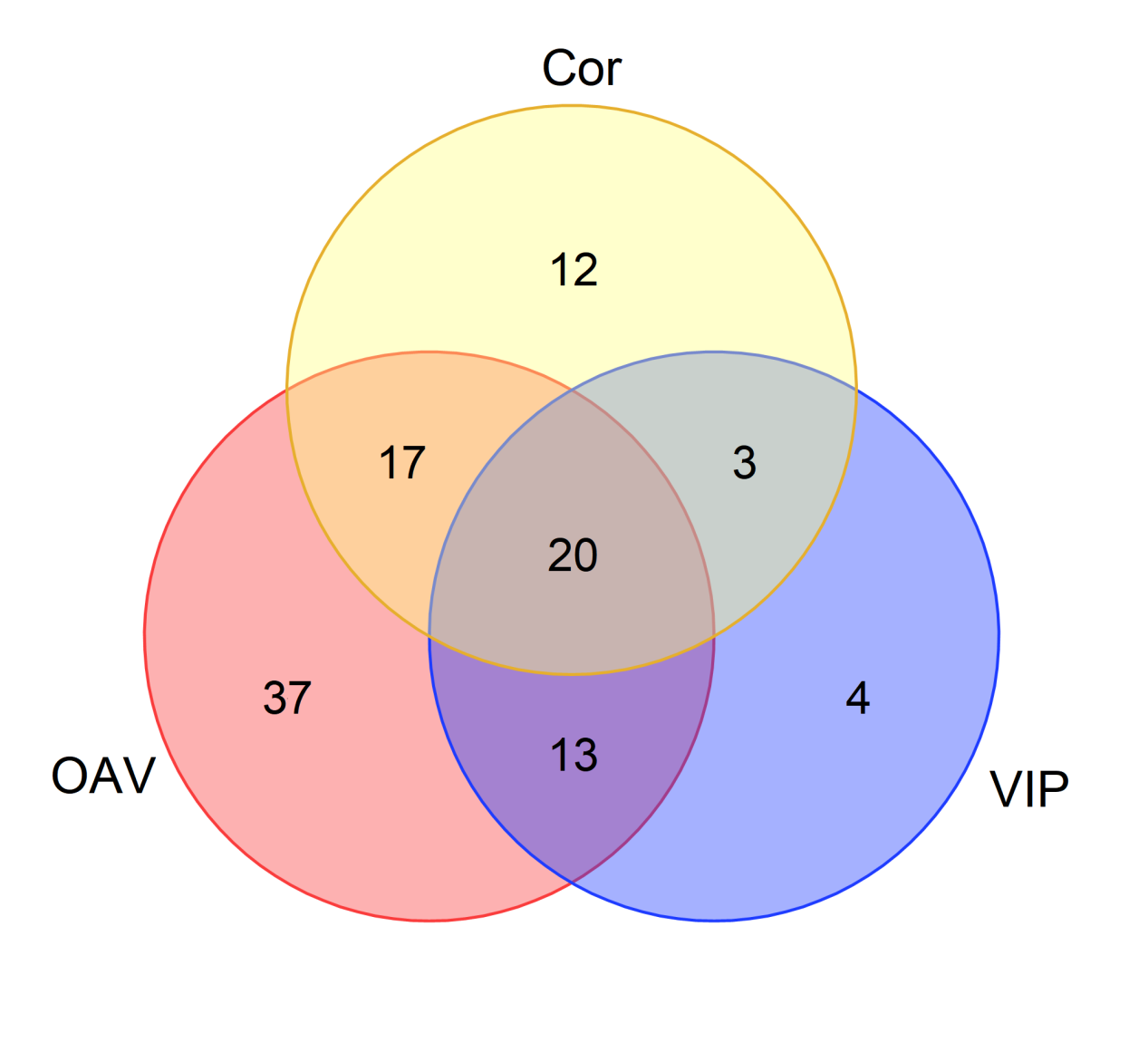
Fig. S6 Screening of key regional markers for core production areas in the Chishui River Basin.

Table S1 40 sauce aroma baijiu (SAB) samples information of different geographical origins

| NO. | Regions | Code | Manufacturers | Produced date | Produced season |
| --- | --- | --- | --- | --- | --- |
| 1 | Maotai Town | MT-1 | Kweichow Moutai Co., Ltd. | 20230915 | Autumn |
| 2 |  | MT-2 | Kweichow Moutai Co., Ltd. | 20240229 | Winter |
| 3 |  | MT-3 | Kweichow Moutai Co., Ltd. | 20241212 | Winter |
| 4 |  | MT-4 | Kweichow Moutai Co., Ltd. | 20231226 | Winter |
| 5 |  | MT-5 | Guizhou Zhongxin Brewing Group Co., Ltd. | 20231026 | Autumn |
| 6 |  | MT-6 | Guizhou Zhongxin Brewing Group Co., Ltd. | 20240123 | Winter |
| 7 |  | MT-7 | Guizhou Guotai Liquor Group Co., Ltd. | 20230926 | Autumn |
| 8 |  | MT-8 | Guizhou Guotai Liquor Group Co., Ltd. | 20230802 | Summer |
| 9 |  | MT-9 | Guizhou Guotai Liquor Group Co., Ltd. | 20231013 | Autumn |
| 10 |  | MT-10 | Guizhou Diaoyutai State Guest Liquor Co., Ltd. | 20230330 | Spring |
| 11 |  | MT-11 | Guizhou Diaoyutai State Guest Liquor Co., Ltd. | 20230310 | Spring |
| 12 |  | MT-12 | Guizhou Diaoyutai State Guest Liquor Co., Ltd. | 20230504 | Spring |
| 13 |  | MT-13 | Guizhou Jinjiang Liquor Co., Ltd. | 20240908 | Autumn |
| 14 |  | MT-14 | Guizhou Jinjiang Liquor Co., Ltd. | 20230219 | Winter |
| 15 |  | MT-15 | Guizhou Hutu Liquor Industry (Group) Co., Ltd. | 20240713 | Summer |
| 16 |  | MT-16 | Guizhou Hutu Liquor Industry (Group) Co., Ltd. | 20230602 | Summer |
| 17 |  | MT-17 | Guizhou Hutu Liquor Industry (Group) Co., Ltd. | 20240611 | Summer |
| 18 |  | MT-18 | Guizhou Huaizhuang Liquor Industry (Group) Co., Ltd. | 20230621 | Summer |
| 19 |  | MT-19 | Guizhou Tangzhuang Liquor Industry Co., Ltd. | 20230602 | Summer |
| 20 |  | MT-20 | Guizhou Tangzhuang Liquor Industry Co., Ltd. | 20230313 | Spring |
| 21 | Xijiu Town | XJ-1 | Guizhou Xijiu Co.,Ltd. | 20230807 | Summer |
| 22 |  | XJ-2 | Guizhou Xijiu Co.,Ltd. | 20240131 | Winter |
| 23 |  | XJ-3 | Guizhou Xijiu Co.,Ltd. | 20230813 | Summer |
| 24 |  | XJ-4 | Guizhou Xijiu Co.,Ltd. | 20230424 | Spring |
| 25 |  | XJ-5 | Guizhou Xijiu Co.,Ltd. | 20231025 | Autumn |
| 26 | Erlang Town | EL-1 | Sichuan Langjiu Group Co., Ltd. | 20240201 | Winter |
| 27 |  | EL-2 | Sichuan Langjiu Group Co., Ltd. | 20241017 | Autumn |
| 28 |  | EL-3 | Sichuan Langjiu Group Co., Ltd. | 20230330 | Spring |
| 29 |  | EL-4 | Sichuan Langjiu Group Co., Ltd. | 20230212 | Winter |
| 30 |  | EL-5 | Sichuan Province Liquor Group Co., Ltd. | 20240730 | Summer |
| 31 |  | EL-6 | Sichuan Province Liquor Group Co., Ltd. | 20230927 | Autumn |
| 32 |  | EL-7 | Sichuan Province Liquor Group Co., Ltd. | 20230808 | Summer |
| 33 |  | EL-8 | Sichuan Province Liquor Group Co., Ltd. | 20240422 | Spring |
| 34 |  | EL-9 | Sichuan Province Liquor Group Co., Ltd. | 20240728 | Summer |
| 35 | Taiping Tow | TP-1 | Sichuan Xiantan Liquor Group Co., Ltd. | 20230807 | Summer |
| 36 |  | TP-2 | Sichuan Xiantan Liquor Group Co., Ltd. | 20241222 | Winter |
| 37 |  | TP-3 | Sichuan Xiantan Liquor Group Co., Ltd. | 20241124 | Autumn |
| 38 |  | TP-4 | Sichuan Xiantan Liquor Group Co., Ltd. | 20230615 | Summer |
| 39 |  | TP-5 | Sichuan Xiantan Liquor Group Co., Ltd. | 20231118 | Spring |
| 40 |  | TP-6 | Sichuan Xiantan Liquor Group Co., Ltd. | 20231106 | Autumn |

Table S2 Qualitative and quantitative parameters of 154 flavor compounds

| Class | NO. | Name | CAS | Methods | Identification | Descriptor | RI | Slope | Intercept | R^2^ | Recovery | LOD (μg/L) |
| --- | --- | --- | --- | --- | --- | --- | --- | --- | --- | --- | --- | --- |
| Esters | 1 | Ethyl formate | 109-94-4 | DI-GC-FID | RI,S | bitter almond | 767 | 0.00171425 | 0.00611078 | 0.9997 | 92.38% | 3.43^a^ |
|  | 2 | Ethyl acetate | 141-78-6 | DI-GC-FID | RI,S | pineapple, fruity | 827 | 0.00313169 | -0.149332 | 0.9999 | 99.13% | 1.33^a^ |
|  | 3 | Propyl acetate | 109-60-4 | DI-GC-FID | RI,S | fruity, sweet, banana | 997 | 0.00326386 | 0.00334827 | 0.9989 | 100.97% | 862 |
|  | 4 | 3-Methylbutyl acetate | 123-92-2 | DI-GC-FID | RI,S | fruity, banana, sweet | 1153 | 0.00451829 | -0.00748137 | 0.9995 | 103.76% | 841 |
|  | 5 | Hexyl acetate | 142-92-7 | HS-SPME Arrow-GC-MS | RI,MS,S | fruity, herb, floral | 1236 | 0.00070237 | -0.00537941 | 0.9939 | 107.88% | 434^b^ |
|  | 6 | 2-Phenylethyl acetate | 103-45-7 | HS-SPME Arrow-GC-MS | RI,MS,S | honey, floral | 1780 | 0.005046618 | 0.1192429 | 0.9811 | 100.24% | 4^b^ |
|  | 7 | Ethyl propanoate | 105-37-3 | DI-GC-FID | RI,S | pineapple, fruity | 970 | 0.00326746 | -0.0152822 | 0.9995 | 113.52% | 1.19^a^ |
|  | 8 | Ethyl 2-methylpropanoate | 97-62-1 | DI-GC-FID | RI,S | fruity, sweet | 1072 | 0.00426163 | -0.000239079 | 0.9992 | 103.26% | 691 |
|  | 9 | Ethyl butanoate | 105-54-4 | DI-GC-FID | RI,S | pineapple, apple | 1055 | 0.00316147 | 0.00840707 | 0.9997 | 113.09% | 1.1^a^ |
|  | 10 | Butyl butanoate | 109-21-7 | HS-SPME Arrow-GC-MS | RI,MS,S | fruity, pineapple, apple, alcoholic | 1187 | 0.03063186 | -0.0786703 | 0.9993 | 109.40% | 170^b^ |
|  | 11 | 3-Methylbutyl butanoate | 106-27-4 | HS-SPME Arrow-GC-MS | RI,MS,S | fruit, floral | 1226 | 0.00116134 | -0.00758476 | 0.9970 | 92.58% | 144^b^ |
|  | 12 | 2-Phenethyl buanoate | 103-52-6 | HS-SPME Arrow-GC-MS | RI,MS,S | honey, sweet, fruity | 1978 | 0.005834553 | -0.1917574 | 0.9937 | 100.83% | 460^b^ |
|  | 13 | Ethyl 2-methylbutanoate | 7452-79-1 | DI-GC-FID | RI,S | wines, berry | 1072 | 0.00396034 | 0.00369797 | 0.9999 | 103.26% | 805 |
|  | 14 | Ethyl 3-methylbutanoate | 108-64-5 | DI-GC-FID | RI,S | apple, alcoholic, fruity | 1087 | 0.00402495 | 0.0037079 | 0.9997 | 105.73% | 743 |
|  | 15 | Ethyl pentanoate | 539-82-2 | HS-SPME Arrow-GC-MS | RI,MS,S | apple, fruity, honey peach | 1118 | 0.00402495 | 0.0037079 | 0.9957 | 99.07% | 269^b^ |
|  | 16 | 3-Methylbutyl pentanoate | 2050-09-1 | HS-SPME Arrow-GC-MS | RI,MS,S | fruity, floral | 1331 | 0.05046637 | -0.050087593 | 0.9976 | 102.28% | 3^b^ |
|  | 17 | Ethyl 4-methylpentanoate | 25415-67-2 | HS-SPME Arrow-GC-MS | RI,MS,S | fruity, floral | 1165 | 0.000839686 | -0.000857414 | 0.9984 | 99.21% | 148^b^ |
|  | 18 | Ethyl hexanoate | 123-66-0 | DI-GC-FID | RI,S | fruity, pineapple, apple, alcoholic | 1255 | 0.00670588 | -0.156268 | 0.9999 | 94.60% | 785 |
|  | 19 | Propyl hexanoate | 626-77-7 | HS-SPME Arrow-GC-MS | RI,MS,S | pineapple, sweet, fruity | 1275 | 0.00048125 | 0.018634516 | 0.9904 | 96.21% | 13^b^ |
|  | 20 | Butyl hexanoate | 626-82-4 | HS-SPME Arrow-GC-MS | RI,MS,S | fruity, sweet, pineapple | 1390 | 0.05961521 | -0.1504093 | 0.9973 | 97.49% | 270^b^ |
|  | 21 | 2-Methylpropyl hexanoate | 105-79-3 | HS-SPME Arrow-GC-MS | RI,MS,S | fruity, alcoholic | 1323 | 0.03506828 | -0.3119736 | 0.9982 | 93.21% | 13^b^ |
|  | 22 | 3-Methylbutyl hexanoate | 2198-61-0 | HS-SPME Arrow-GC-MS | RI,MS,S | fruity, apple | 1432 | 0.00013231 | 0.007928122 | 0.9929 | 93.81% | 1.68 |
|  | 23 | Hexyl hexanoate | 6378-65-0 | HS-SPME Arrow-GC-MS | RI,MS,S | fruity, peach, apple | 1580 | 0.001048439 | -0.0341501 | 0.9979 | 96.52% | 860^b^ |
|  | 24 | 2-Phenethyl hexanoate | 6290-37-5 | HS-SPME Arrow-GC-MS | RI,MS,S | flower, fruity | 2190 | 0.000221869 | -0.001225199 | 0.9938 | 114.57% | 1.07 |
|  | 25 | Ethyl 5-methylhexanoate | 10236-10-9 | HS-SPME Arrow-GC-MS | RI,MS,S | pineapple | 1261 | 0.010936005 | -0.002650563 | 0.9982 | 94.75% | 9^b^ |
|  | 26 | Ethyl heptanoate | 106-30-9 | HS-SPME Arrow-GC-MS | RI,MS,S | fruity | 1309 | 0.002847137 | 0.08070335 | 0.9922 | 105.22% | 27^b^ |
|  | 27 | Ethyl octanoate | 106-32-1 | HS-SPME Arrow-GC-MS | RI,MS,S | fruity, pear, floral | 1411 | 0.003316345 | 0.085495204 | 0.9996 | 95.07% | 18^b^ |
|  | 28 | Ethyl nonanoate | 123-29-5 | HS-SPME Arrow-GC-MS | RI,MS,S | soapy | 1509 | 0.002364932 | -0.011647014 | 0.9871 | 93.07% | 40^b^ |
|  | 29 | Ethyl decanoate | 110-38-3 | HS-SPME Arrow-GC-MS | RI,MS,S | floral, fruity, wines | 1612 | 0.008137348 | -0.01671941 | 0.9816 | 100.56% | 16^b^ |
|  | 30 | (E)-Ethyl 4-decenoate | 76649-16-6 | HS-SPME Arrow-GC-MS | RI,MS,S | green, greasy, floral | 1669 | 0.001771759 | 0.02106382 | 0.9984 | 102.06% | 53^b^ |
|  | 31 | Ethyl undecanoate | 627-90-7 | HS-SPME Arrow-GC-MS | RI,MS,S | coconut,fruity,nutty,fatty | 1721 | 0.01048558 | 0.04371799 | 0.9998 | 96.61% | 21^b^ |
|  | 32 | Ethyl dodecanoate | 106-33-2 | HS-SPME Arrow-GC-MS | RI,MS,S | fruity, floral, soapy | 1849 | 0.01094894 | -0.3743211 | 0.9813 | 105.70% | 35^b^ |
|  | 33 | Ethyl tridecanoate | 28267-29-0 | HS-SPME Arrow-GC-MS | RI,MS,S | coke, sesame | 1951 | 0.002522265 | 0.1638868 | 0.9937 | 102.23% | 67^b^ |
|  | 34 | Ethyl tetradecanoate | 124-06-1 | HS-SPME Arrow-GC-MS | RI,MS,S | alcoholic | 2054 | 0.001824134 | 0.09713888 | 0.9945 | 101.42% | 76^b^ |
|  | 35 | Ethyl pentadecanoate | 41114-00-5 | HS-SPME Arrow-GC-MS | RI,MS,S | sweet baked | 2160 | 0.002171949 | -0.007326631 | 0.9926 | 105.08% | 20^b^ |
|  | 36 | Ethyl hexadecanoate | 628-97-7 | DI-GC-FID | RI,S | wax, grease, frankincense | 2205 | 0.00892339 | -0.122556 | 0.9998 | 102.58% | 1.14^a^ |
|  | 37 | Ethyl 9-hexadecenoate | 54546-22-4 | DI-GC-FID | RI,MS,S | fruity | 2233 | 0.00332161 | -0.000850517 | 0.9989 | 98.32% | 1.47^a^ |
|  | 38 | Ethyl heptadecanoate | 14010-23-2 | HS-SPME Arrow-GC-MS | RI,MS,S | pungent,waxy | 2378 | 0.001876357 | -0.04645924 | 0.9941 | 90.06% | 500^b^ |
|  | 39 | Ethyl octadecanoate | 111-61-5 | HS-SPME Arrow-GC-MS | RI,MS,S | waxy | 2618 | 0.003062419 | -0.1735975 | 0.9935 | 101.16% | 28^b^ |
|  | 40 | Ethyl oleate | 111-62-6 | DI-GC-FID | RI,S | floral, fatty | 2430 | 0.00744876 | -0.0187246 | 0.9979 | 96.88% | 1.19^a^ |
|  | 41 | Ethyl linoleate | 544-35-4 | DI-GC-FID | RI,S | floral, fatty, fruity | 2480 | 0.00763481 | -0.0202806 | 0.9997 | 101.48% | 833 |
|  | 42 | Ethyl linolenate | 1191-41-9 | HS-SPME Arrow-GC-MS | RI,MS,S | milky sweet roast | 2632 | 0.005336518 | -0.2013854 | 0.9967 | 98.29% | 840^b^ |
|  | 43 | Diethyl butanedioate | 123-25-1 | HS-SPME Arrow-GC-MS | RI,MS,S | fruity | 1646 | 0.000254868 | -0.00291884 | 0.9964 | 96.04% | 144^b^ |
|  | 44 | Diethyl azeleate | 624-17-9 | HS-SPME Arrow-GC-MS | RI,MS,S | sweet | 2224 | 0.000210544 | 0.0034624 | 0.9710 | 106.93% | 421^b^ |
|  | 45 | Ethyl lactate | 105-58-8 | DI-GC-FID | RI,S | fruity, alcoholic | 2369 | 0.00318663 | 0.0329147 | 0.9993 | 96.54% | 1.41^a^ |
|  | 46 | 3-Methylbutyl lactate | 19329-89-6 | HS-SPME Arrow-GC-MS | RI,MS,S | fruity, creamy | 1573 | 0.000145291 | 0.01825194 | 0.9854 | 95.17% | 807^b^ |
|  | 47 | Ethyl 2-furancarboxylate | 614-99-3 | HS-SPME Arrow-GC-MS | RI,MS,S | green apple，kiwifruit，mushroom | 1628 | 0.000586947 | -0.001064922 | 0.9983 | 103.14% | 151^b^ |
|  | 48 | Ethyl 2-hydroxy-4-methylpentanoate | 10348-47-7 | DI-GC-FID | RI,S | fruity, blackberry, freshness | 1573 | 0.00461878 | 0.00452317 | 0.9987 | 92.23% | 911 |
|  | 49 | Ethyl 2-hydroxy-hexanoate | 52089-55-1 | HS-SPME Arrow-GC-MS | RI,MS,S | floral | 1596 | 0.000326123 | 0.01558512 | 0.9981 | 95.63% | 186^b^ |
|  | 50 | Ethyl benzoate | 93-89-0 | HS-SPME Arrow-GC-MS | RI,MS,S | honey-like, fruity, floral | 1639 | 0.000598275 | 0.004087439 | 0.9981 | 101.05% | 7.8^b^ |
|  | 51 | Ethyl phenylacetate | 101-97-3 | DI-GC-FID | RI,S | rose, honey, sweet, fruity | 1739 | 0.00764634 | 0.00269723 | 0.9999 | 81.38% | 596 |
|  | 52 | Ethyl 3-phenylpropionate | 2021-28-5 | HS-SPME Arrow-GC-MS | RI,MS,S | fruity, rose, wine, honey, floral | 1984 | 0.009397577 | 0.02939956 | 0.9973 | 108.76% | 216^b^ |
| Alcohols | 53 | Methanol | 67-56-1 | DI-GC-FID | RI,S | alcoholic | 854 | 0.00237223 | -0.0452275 | 0.9997 | 98.00% | 3.31^a^ |
|  | 54 | 1-Propanol | 71-23-8 | DI-GC-FID | RI,S | alcoholic, earthy, yeasty, fruity | 1059 | 0.00494588 | -0.189811 | 0.9995 | 100.16% | 1.04^a^ |
|  | 55 | 1-Butanol | 71-36-3 | DI-GC-FID | RI,S | fruity, alcoholic, medicine | 1168 | 0.00506429 | -0.104231 | 0.9994 | 106.94% | 514 |
|  | 56 | 2-Butanol | 78-92-2 | DI-GC-FID | RI,S | fruity, alcoholic | 1046 | 0.00404549 | 0.0449259 | 0.9993 | 113.35% | 680 |
|  | 57 | 2-Methylpropanol | 78-83-1 | DI-GC-FID | RI,S | pine and cypress incense, wine, fruity, malt | 1119 | 0.00533054 | -0.123265 | 0.999 | 98.99% | 572 |
|  | 58 | 1-Pentanol | 71-41-0 | DI-GC-FID | RI,S | fruity, ester | 1274 | 0.00669289 | -0.0361851 | 0.9991 | 83.06% | 621 |
|  | 59 | 2-Pentanol | 6032-29-7 | DI-GC-FID | RI,S | fruity, alcoholic, alcoholic | 1145 | 0.00401707 | -0.000764959 | 0.9998 | 115.24% | 540 |
|  | 60 | 2-Methylbutanol | 137-32-6 | DI-GC-FID | RI,S | alcoholic, fruity | 1229 | 0.00668403 | 0.0898805 | 0.999 | 110.30% | 1.6^a^ |
|  | 61 | 3-Methylbutanol | 123-51-3 | DI-GC-FID | RI,S | alcohol-like, pungent, nail polish, fruity, malt | 1231 | 0.00615754 | 0.0144375 | 0.9991 | 95.55% | 830 |
|  | 62 | 1-Hexanol | 111-27-3 | DI-GC-FID | RI,S | fruity, herbal, floral | 1378 | 0.00663931 | 0.00785228 | 0.9993 | 91.44% | 580 |
|  | 63 | 1-Heptanol | 111-70-6 | HS-SPME Arrow-GC-MS | RI,MS,S | alcoholic, fruity | 1424 | 0.009734137 | -0.1223269 | 0.9953 | 92.09% | 40^b^ |
|  | 64 | 2-Heptanol | 543-49-7 | HS-SPME Arrow-GC-MS | RI,MS,S | fruity, citrus | 1292 | 0.001644317 | 0.0350805 | 0.995 | 86.32% | 38^b^ |
|  | 65 | 1-Octanol | 111-87-5 | HS-SPME Arrow-GC-MS | RI,MS,S | fruity, wax, alcoholic, citrus | 1527 | 0.000931463 | 0.001280388 | 0.9978 | 101.45% | 32^b^ |
|  | 66 | 1-Octen-3-ol | 3391-86-4 | HS-SPME Arrow-GC-MS | RI,MS,S | mushroom, earthy | 1416 | 0.01328713 | -0.009324888 | 0.9968 | 99.52% | 138^b^ |
|  | 67 | 3-Octanol | 589-98-0 | HS-SPME Arrow-GC-MS | RI,MS,S | green, mushroom | 1384 | 0.001759443 | -0.007228404 | 0.9975 | 94.34% | 16^b^ |
|  | 68 | 1-Nonanol | 143-08-8 | HS-SPME Arrow-GC-MS | RI,MS,S | floral, alcoholic | 1662 | 0.003731222 | -0.000780523 | 0.9968 | 99.86% | 22^b^ |
|  | 69 | 2-Nonanol | 628-99-9 | HS-SPME Arrow-GC-MS | RI,MS,S | floral, fruity | 1515 | 0.00823777 | 0.07952195 | 0.9975 | 100.88% | 36^b^ |
|  | 70 | (E)-2-Nonen-1-ol | 31502-14-4 | HS-SPME Arrow-GC-MS | RI,MS,S | rhizome-like | 1678 | 0.000386534 | -0.02756303 | 0.9976 | 100.41% | 88^b^ |
|  | 71 | 1-Decanol | 112-30-1 | HS-SPME Arrow-GC-MS | RI,MS,S | anxious burnt, acid, sweet | 1767 | 0.000812013 | 0.01291199 | 0.9968 | 96.84% | 245^b^ |
|  | 72 | 2-Undecanol | 1653-30-1 | HS-SPME Arrow-GC-MS | RI,MS,S | grassy | 1722 | 0.002458182 | 0.00325616 | 0.9988 | 99.44% | 25^b^ |
|  | 73 | 1,2-Propanediol | 57-55-6 | DI-GC-FID | RI,S | alcoholic | 1526 | 0.0230985 | -0.294287 | 0.9994 | 115.11% | 1.53^a^ |
|  | 74 | (R,R)-2,3-Butanediol | 24347-58-8 | DI-GC-FID | RI,S | sweet,fruity | 1486 | 0.00316755 | -0.0412793 | 0.9996 | 96.83% | 830 |
|  | 75 | Meso-2,3-Butanediol | 513-85-9 | DI-GC-FID | RI,S | paint,green | 1508 | 0.00929233 | -0.0179501 | 0.9995 | 107.64% | 1.23^a^ |
|  | 76 | Benzyl alcohol | 100-51-6 | HS-SPME Arrow-GC-MS | RI,MS,S | floral, phenolic, bitter almond odour | 1833 | 0.000810001 | 0.01924986 | 0.9907 | 96.46% | 219^b^ |
|  | 77 | 2-Phenethyl alcohol | 60-12-8 | DI-GC-FID | RI,S | rose, honey | 1875 | 0.0122711 | 0.000591923 | 0.9996 | 96.46% | 560 |
| Acids | 78 | Formic acid | 64-18-6 | DI-HPLC | S | / | / | 0.000211 | -0.28901 | 0.9995 | 90.51% | 150 |
|  | 79 | Acetic acid | 64-19-7 | DI-GC-FID | RI,S | acid, vinegar | 1469 | 0.0191522 | -0.818693 | 0.9997 | 105.78% | 3.87^a^ |
|  | 80 | Propionic acid | 79-09-4 | DI-GC-FID | RI,S | acid, vinegar, sweat, cheesy | 1554 | 0.0283844 | -0.271208 | 0.9999 | 97.24% | 1.9^a^ |
|  | 81 | Butanoic acid | 107-92-6 | DI-GC-FID | RI,S | sour and stinky, pit mud, rancid, cheesy | 1642 | 0.0372533 | -0.324223 | 0.9998 | 99.39% | 1.5^a^ |
|  | 82 | 2-Methylpropanoic acid | 79-31-2 | DI-GC-FID | RI,S | cheesy, sweat, rancid, rotten | 1581 | 0.0387306 | -0.493508 | 0.9999 | 109.47% | 1.32^a^ |
|  | 83 | Pentanoic acid | 109-52-4 | DI-GC-FID | RI,S | sweat, acid, pit mud, cheesy, sour and stinky | 1755 | 0.0441332 | -0.704710 | 0.9999 | 107.53% | 1.06^a^ |
|  | 84 | 3-Methylbutanoic acid | 503-74-2 | DI-GC-FID | RI,S | sweat, acid, cheesy, fatty | 1684 | 0.02131217 | -0.854417 | 0.9983 | 113.87% | 3.03^a^ |
|  | 85 | Hexanoic acid | 142-62-1 | DI-GC-FID | RI,S | sweat, pungent, cheesy, acid, fatty | 1862 | 0.0555107 | -0.633177 | 0.9999 | 108.69% | 812 |
|  | 86 | Octanoic acid | 124-07-2 | DI-GC-FID | RI,S | cheesy, sweat, fatty, rotten | 2076 | 0.0561282 | -1.04933 | 0.9998 | 109.89% | 81 |
|  | 87 | Decanoic acid | 334-48-5 | DI-GC-FID | RI,S | bad odour, sweat, fatty | 2290 | 0.0589931 | -0.513601 | 0.9998 | 106.77% | 910 |
|  | 88 | Dodecanoic acid | 143-07-7 | DI-GC-FID | RI,S | fatty | 2489 | 0.285553 | -0.0754785 | 0.9893 | 101.37% | 190 |
|  | 89 | Benzoic acid | 65-85-0 | HS-SPME Arrow-GC-MS | RI,MS,S | fruity, astringency, urine | 2337 | 1.98918E-05 | 0.0237988 | 0.9837 | 111.69% | 42^b^ |
|  | 90 | L-Lactic acid | 79-33-4 | DI-HPLC | S | / | / | 0.000315 | -0.7727 | 0.9998 | 105.86% | 300 |
|  | 91 | Citric acid | 77-92-9 | DI-HPLC | S | / | / | 0.000109 | 0.1769 | 0.9998 | 103.16% | 300 |
|  | 92 | Palmitic acid | 57-10-3 | DI-UPLC-MS/MS | S | / | / | 25504 | 9892204 | 0.9989 | 86.14% | 55 |
|  | 93 | Oleic acid | 112-80-1 | DI-UPLC-MS/MS | S | / | / | 82286 | 54143972 | 0.9984 | 103.29% | 51 |
|  | 94 | Linoleic acid | 60-33-3 | DI-UPLC-MS/MS | S | / | / | 75552 | 847518 | 0.9986 | 99.87% | 35 |
| Aldehydes | 95 | Acetaldehyde | 75-07-0 | DI-GC-FID | RI,S | astringency, grassy, fruity | 715 | 0.00215964 | -0.0207355 | 0.9995 | 94.67% | 3.08^a^ |
|  | 96 | 2-Methylpropanal | 78-84-2 | DI-GC-FID | RI,S | alcoholic,green | 759 | 0.00338461 | -0.00193973 | 0.999 | 90.25% | 1.51^a^ |
|  | 97 | 2-Methylbutanal | 96-17-3 | DI-GC-FID | RI,S | roasted, coffee, cocoa, mouldy, almonds | 884 | 0.00440931 | 0.0045129 | 0.9995 | 93.75% | 1.12^a^ |
|  | 98 | 3-Methylbutanal | 590-86-3 | DI-GC-FID | RI,S | malt, alcoholic, cocoa | 895 | 0.00325593 | 0.00181137 | 0.9997 | 106.61% | 1.58^a^ |
|  | 99 | Nonanal | 124-19-6 | HS-SPME Arrow-GC-MS | RI,MS,S | waxy, fatty, soap, grassy | 1368 | 0.01543072 | -0.334055 | 0.9942 | 90.28% | 170^b^ |
|  | 100 | Benzaldehyde | 100-52-7 | DI-GC-FID | RI,S | apricot, cherry, nutty, fruity | 1463 | 0.00726397 | 0.00223934 | 0.9918 | 110.31% | 790 |
|  | 101 | Benzeneacetaldehyde | 122-78-1 | HS-SPME Arrow-GC-MS | RI,MS,S | floral, honey, sweet | 1618 | 0.000244885 | 0.000365737 | 0.9941 | 102.26% | 388^b^ |
| Acetals | 102 | Diethoxymethane | 462-95-3 | DI-GC-FID | RI,S | Irritating, chloroform | 785 | 0.00317708 | 0.00412304 | 0.9994 | 116.88% | 1.87^a^ |
|  | 103 | 1,1-Diethoxyethane | 105-57-7 | DI-GC-FID | RI,S | fruity, fruits, nutty, earthy | 836 | 0.00378736 | -0.0261421 | 0.9993 | 87.89% | 1.94^a^ |
|  | 104 | 1,1-Diethoxy-3-methylbutane | 3842-03-3 | DI-GC-FID | RI,S | fruity, fatty, grassy | 1073 | 0.00230971 | 0.00510716 | 0.9948 | 108.52% | 1.1^a^ |
|  | 105 | (2,2-Diethoxyethyl)-benzene | 6314-97-2 | HS-SPME Arrow-GC-MS | RI,MS,S | sweet lemon, floral, fruity | 1685 | 0.004628562 | -0.05997597 | 0.9857 | 84.64% | 35^b^ |
|  | 106 | 1,1,3,3-Tetraethoxy-propane | 122-31-6 | HS-SPME Arrow-GC-MS | RI,MS,S | pungent | 1471 | 0.01007593 | -0.048388597 | 0.9969 | 82.90% | 37^b^ |
| Ketones | 107 | Acetone | 67-64-1 | DI-GC-FID | RI,S | sweet, spicy | 760 | 0.00323099 | -0.00279067 | 0.9999 | 81.44% | 1.24^a^ |
|  | 108 | 2-Pentanone | 107-87-9 | DI-GC-FID | RI,S | fruity | 1001 | 0.00189942 | -0.00159095 | 0.9971 | 97.66% | 1.08^a^ |
|  | 109 | 3-Hydroxyl-2-butanone | 513-86-0 | DI-GC-FID | RI,S | grease, creamy, sweet, fatty | 1313 | 0.00549853 | 0.00754853 | 0.9994 | 102.41% | 1.24^a^ |
|  | 110 | 2-Heptanone | 110-43-0 | HS-SPME Arrow-GC-MS | RI,MS,S | cheese, fruity, copra, pear | 1165 | 0.04080573 | -0.02387884 | 0.9998 | 95.17% | 38^b^ |
|  | 111 | 2-Octanone | 111-13-7 | HS-SPME Arrow-GC-MS | RI,MS,S | floral, fruity, mouldy, herbal | 1272 | 0.660057 | -0.06551658 | 0.9986 | 98.02% | 0.7^b^ |
|  | 112 | 3-Octanone | 106-68-3 | HS-SPME Arrow-GC-MS | RI,MS,S | fumes, aldehyde | 1239 | 0.01454796 | -0.05914169 | 0.9999 | 95.18% | 50^b^ |
|  | 113 | 2-Nonanone | 821-55-6 | HS-SPME Arrow-GC-MS | RI,MS,S | floral, herbal, fruity | 1383 | 0.003856471 | 0.072041872 | 0.9946 | 98.02% | 16^b^ |
|  | 114 | 4-Nonanone | 4485-09-0 | HS-SPME Arrow-GC-MS | RI,MS,S | fatty,waxy | 1296 | 0.03617427 | 0.02011936 | 0.9985 | 101.43% | 22^b^ |
|  | 115 | 2-Undecanone | 112-12-9 | HS-SPME Arrow-GC-MS | RI,MS,S | peach, fatty, fruity, sweet, waxy | 1622 | 0.009889095 | 0.064715657 | 0.9996 | 101.16% | 8^b^ |
|  | 116 | 4-Undecanone | 14476-37-0 | HS-SPME Arrow-GC-MS | RI,MS,S | fatty,waxy | 1505 | 0.000410908 | 0.05003198 | 0.9859 | 87.25% | 25^b^ |
|  | 117 | 2-Tridecanone | 593-08-8 | HS-SPME Arrow-GC-MS | RI,MS,S | grease, fatty | 1816 | 0.007861851 | 0.06676557 | 0.9998 | 102.21% | 8^b^ |
|  | 118 | 2-Pentadecanone | 2345-28-0 | HS-SPME Arrow-GC-MS | RI,MS,S | cream, fatty | 2031 | 0.005886244 | -0.01555091 | 0.9934 | 98.66% | 14^b^ |
| Furans | 119 | Furfuryl alcohol | 98-00-0 | DI-GC-FID | RI,S | caramel, mouldy, alcoholic, alcoholic | 1595 | 0.00451946 | -0.0146611 | 0.9998 | 92.53% | 900 |
|  | 120 | Furfural | 98-01-01 | DI-GC-FID | RI,S | sweet, caramel, woody, mouldy, baked, nutty, roasted | 1374 | 0.00414733 | -0.040059 | 0.9999 | 98.60% | 130 |
|  | 121 | 5-Methylfurfural | 620-02-0 | HS-SPME Arrow-GC-MS | RI,MS,S | sweet, caramel, baked, nutty, roasted | 1550 | 0.003044121 | 0.04216675 | 0.9984 | 107.97% | 54^b^ |
|  | 122 | Furfuryl ethyl ether | 6270-56-0 | DI-GC-FID | RI,S | coffee,roast | 1307 | 0.00469313 | -0.00170086 | 0.9999 | 104.29% | 1.38^a^ |
|  | 123 | 2-Acetylfuran | 1192-62-7 | HS-SPME Arrow-GC-MS | RI,MS,S | sweet, caramel, nutty | 1480 | 0.001032797 | -0.02204839 | 0.9915 | 93.66% | 123^b^ |
|  | 124 | HDMF | 3658-77-3 | LLE-GC-MS | RI,MS,S | caramel, spice | 2041 | 0.00009081 | -0.0003913 | 0.9996 | 93.03% | 4.24 |
|  | 125 | HEMF | 27538-10-9 | LLE-GC-MS | RI,MS,S | caramel, spice | 2088 | 0.00007448 | 0.0005959 | 0.9932 | 103.85% | 8.46 |
|  | 126 | Sotolon | 28664-35-9 | LLE-GC-MS | RI,MS,S | caramel, spice, herb | 2213 | 0.0004948 | -0.004127 | 0.9817 | 86.9%% | 1.6 |
| Pyrazines | 127 | Pyrazine | 290-37-9 | DI-UPLC-MS/MS | S | green | / | 118.87 | -53.175 | 0.9972 | 99.65% | 95.21 |
|  | 128 | 2-Methylpyrazine | 109-08-0 | DI-UPLC-MS/MS | S | cocoa, hazelnuts, popcorn, roast | / | 113.19 | 5747.8 | 0.9954 | 85.46% | 38.24 |
|  | 129 | 2,3-Dimethylpyrazine | 5910-89-4 | DI-UPLC-MS/MS | S | roast, nut | / | 437.55 | 19896 | 0.9993 | 103.71% | 3.66 |
|  | 130 | 2,5-Dimethylpyrazine | 123-32-0 | DI-UPLC-MS/MS | S | cocoa, roasted nut | / | 558.45 | 37952 | 0.9991 | 98.37% | 970^b^ |
|  | 131 | 2,6-Dimethylpyrazine | 108-50-9 | DI-UPLC-MS/MS | S | rice, nut, roasted, mouldy | / | 294.17 | 24871 | 0.9948 | 92.11% | 3.29 |
|  | 132 | 2-Ethylpyrazine | 13925-00-3 | DI-UPLC-MS/MS | S | roasted,rum,woody | / | 403.64 | 33276 | 0.9991 | 105.67% | 2.02 |
|  | 133 | 2-Ethyl-3-methylpyrazine | 15707-23-0 | DI-UPLC-MS/MS | S | green, musty, nuts, potatoes, barbecue | / | 1103.3 | 65796 | 0.9993 | 102.50% | 2.1 |
|  | 134 | 2-Ethyl-6-methylpyrazine | 13925-03-6 | DI-UPLC-MS/MS | S | baked, nutty, roasted, baked potato | / | 585.47 | 259196 | 0.996 | 88.84% | 0.26 |
|  | 135 | 2,3,5-Trimethylpyrazine | 14667-55-1 | DI-UPLC-MS/MS | S | peanuts, roasted, earthy mouldy, roasted nut | / | 532.09 | 287829 | 0.9973 | 90.93% | 2.11 |
|  | 136 | 2-Ethyl-3,5-dimethylpyrazine | 13925-07-0 | DI-UPLC-MS/MS | S | roasted potato | / | 1779 | 102243 | 0.9983 | 82.22% | 4.81 |
|  | 137 | 2-Ethyl-3,6-dimethylpyrazine | 13360-65-1 | DI-UPLC-MS/MS | S | roast, nut, roasted | / | 1392.2 | 41383 | 0.9982 | 89.49% | 3.01 |
|  | 138 | 2,3,5,6-Tetramethylpyrazine | 1124-11-4 | DI-UPLC-MS/MS | S | peanuts, nutty, baked, baked potato, roasted | / | 1515.9 | 937018 | 0.9974 | 96.53% | 4.49 |
|  | 139 | 2,3-Diethylpyrazine | 15707-24-1 | DI-UPLC-MS/MS | S | baked | / | 154.41 | 11312 | 0.9973 | 96.48% | 3.95 |
|  | 140 | 2,3-Diethyl-5-methylpyrazine | 18138-04-0 | DI-UPLC-MS/MS | S | smoky, woody | / | 484.74 | 15324 | 0.998 | 96.15% | 4.36 |
|  | 141 | 2-Isobutyl-3-methylpyrazine | 13925-06-9 | DI-UPLC-MS/MS | S | green | / | 272.15 | 14141 | 0.9962 | 99.55% | 3.34 |
|  | 142 | (Trimethylpyrazin-2-yl)methanol | 75907-74-3 | DI-UPLC-MS/MS | S | nutty, baked | / | 9432.2 | 89491 | 0.9979 | 84.47% | 4.09 |
| Sulfide | 143 | Dimethyl trisulfide | 3658-80-8 | HS-SPME Arrow-GC-MS | RI,MS,S | sulfur, cabbage, onion, salted vegetables | 1354 | 0.00743048 | -0.053202286 | 0.9754 | 99.39% | 32 |
| Phenols | 144 | Phenol | 108-95-2 | HS-SPME Arrow-GC-MS | RI,MS,S | phenolic flavor, medicine, feed-like, caoutchouc | 1947 | 0.000263954 | 0.02475722 | 0.9858 | 96.65% | 249^b^ |
|  | 145 | p-Cresol | 106-44-5 | HS-SPME Arrow-GC-MS | RI,MS,S | smoke | 2010 | 0.004679139 | 0.05061239 | 0.986 | 111.17% | 49^b^ |
|  | 146 | Guaiacol | 90-05-1 | HS-SPME Arrow-GC-MS | RI,MS,S | woody | 1819 | 0.002829766 | -0.03545961 | 0.9971 | 95.73% | 85^b^ |
|  | 147 | 4-Methylguaiacol | 93-51-6 | HS-SPME Arrow-GC-MS | RI,MS,S | fumes, phenolic aldehyde, medicine, lilac, smoke | 1903 | 0.000500902 | 0.004517031 | 0.9928 | 97.39% | 286^b^ |
|  | 148 | 4-Ethylguaiacol | 2785-89-9 | HS-SPME Arrow-GC-MS | RI,MS,S | lilac, smoke, phenolic flavor, clove, spicy | 1969 | 0.01657396 | -0.007261659 | 0.9972 | 99.50% | 14^b^ |
|  | 149 | 4-Vinylguaiacol | 7786-61-0 | HS-SPME Arrow-GC-MS | RI,MS,S | woody, Chinese medicine | 2116 | 0.000599781 | -0.005414437 | 0.9968 | 96.93% | 755^b^ |
| Lactones | 150 | gama-Nonalactone | 104-61-0 | HS-SPME Arrow-GC-MS | RI,MS,S | creamy, copra, sweet | 1972 | 0.007742114 | 0.02896136 | 0.9935 | 97.61% | 16^b^ |
|  | 151 | gama-Dodecalactone | 2305-05-7 | HS-SPME Arrow-GC-MS | RI,MS,S | sweet, coconut | 2278 | 0.000684327 | 0.01924986 | 0.9921 | 99.77% | 35^b^ |
| Terpenes | 152 | Phytone | 502-69-2 | HS-SPME Arrow-GC-MS | RI,MS,S | mushroom, acid | 2044 | 0.001164041 | -7.17472E-05 | 0.9826 | 85.25% | 66^b^ |
|  | 153 | beta-Damascone | 23696-85-7 | HS-SPME Arrow-GC-MS | RI,MS,S | honey, sweet, floral | 1760 | 0.06281435 | -0.03113523 | 0.9994 | 96.99% | 4^b^ |
|  | 154 | Geranylacetone | 3796-70-1 | HS-SPME Arrow-GC-MS | RI,MS,S | floral,rose,honey | 1810 | 0.004027097 | 0.002434598 | 0.9896 | 101.42% | 19^b^ |

^a^The unit of compound is mg/L.

^b^The unit of compound is ng/L.

Table S3 The average content of 154 flavor compounds in MT towns samples (MT-1 to MT-10) (μg/L)

| Name | MT-1 | MT-2 | MT-3 | MT-4 | MT-5 | MT-6 | MT-7 | MT-8 | MT-9 | MT-10 |
| --- | --- | --- | --- | --- | --- | --- | --- | --- | --- | --- |
| Ethyl formate | 37918.15±1580.55 | 36693.24±716.89 | 32916.85±463.67 | 39359.98±1163.96 | 36903.82±1400.67 | 8187.97±789.11 | 48388.92±955.93 | 44424.94±575.03 | 48695.76±807.14 | 38830.78±893.31 |
| Ethyl acetate^a^ | 2889.84±73.21 | 3063.73±55.33 | 2859.97±55.72 | 3159.17±61.42 | 3096.83±63.07 | 1205.5±11.87 | 2626.5±81.27 | 2650.73±26.77 | 2835.58±32.05 | 2478.53±48.99 |
| Propyl acetate | 7904.52±943.47 | 19737.53±1137.65 | 26370.41±525.96 | 27943.78±1058.81 | 37396.89±943.36 | 19151.26±128.96 | 12872.01±370.28 | 19395.92±272.24 | 22290.39±8.74 | 12660.15±861.65 |
| 3-Methylbutyl acetate | 3137.79±60.23 | 5827.91±20.39 | 3320.49±104.58 | 3896.67±227.24 | 9850.72±88.19 | 9396.64±325.5 | 3476.09±24.05 | 3596.51±42.01 | 3733.82±6.72 | 3732.26±82.63 |
| Hexyl acetate | 255.37±3.47 | 137.87±11.1 | 133.26±16.6 | 165.68±7.03 | 77.27±7.96 | 120.34±5.21 | 445.9±15.63 | 1194.42±59.95 | 162.72±10.66 | 297.34±8.54 |
| 2-Phenylethyl acetate | 35.61±2.53 | 230.33±10.74 | 134.97±4.4 | 146.65±13.55 | 147.06±4.42 | 176.31±6.87 | 97.41±4.97 | 298.5±15.56 | 31.2±1.12 | 108.16±9.69 |
| Ethyl propanoate | 30741.94±1930.04 | 40602.61±1249.78 | 52519.76±1394.12 | 63376.08±2828.84 | 71586.37±1117.41 | 40179.34±1340.49 | 65910.29±1686.81 | 108986.72±1481.91 | 77209.52±458.47 | 62280.59±996.65 |
| Ethyl 2-methylpropanoate | 16957.6±1022.42 | 22165.74±1045.71 | 21550.04±1059.5 | 18243.41±585.03 | 17359.45±337.7 | 9377.76±330.7 | 13458.53±360.2 | 14804.22±67.47 | 9205.77±350.22 | 15581.21±479.59 |
| Ethyl butanoate | 39891.75±1993.01 | 25667.81±821.13 | 18823.3±753.41 | 27582.94±1187.44 | 20834.9±615.99 | 15304.68±252.86 | 76997.79±1674.06 | 71755.82±1348.69 | 72352.95±1601.75 | 89888.63±1674.83 |
| Butyl butanoate | 65.76±4.73 | 57.55±3.62 | 33.43±2.07 | 36.44±2.26 | 31.65±0.25 | 114.99±6.63 | 75.87±0.43 | 59.01±1.92 | 35.43±0.88 | 148.42±9.5 |
| 3-Methylbutyl butanoate | 219.32±23.87 | 235.33±2.46 | 20.52±0.72 | 180.45±12.28 | 0±0 | 0±0 | 480.33±44.72 | 713.1±13.1 | 129.46±12.67 | 537.67±8.57 |
| 2-Phenethyl buanoate | 452.16±33.93 | 352.34±28.52 | 249.44±23.73 | 276.06±22.21 | 221.8±14.03 | 418.37±40.58 | 1432.31±38.79 | 663.34±27.93 | 595.27±11.67 | 802.61±6.54 |
| Ethyl 2-methylbutanoate | 3323.91±393.25 | 4889.44±273.21 | 4491.39±240.3 | 4279.33±256.81 | 3700.26±184.46 | 2287.94±98.11 | 2328.01±101.45 | 2787.78±81.1 | 2693.01±118.53 | 2389.63±80.64 |
| Ethyl 3-methylbutanoate | 11607.69±578.68 | 16658.89±524.92 | 15557.66±695.1 | 13815.53±563.29 | 13300.95±396.98 | 9505.19±152.29 | 8028.97±298.76 | 9582.95±305.78 | 6471.95±78.93 | 8503.35±243.67 |
| Ethyl pentanoate | 3586.68±169.46 | 1783.01±101.87 | 786.05±12.4 | 3347.21±353.56 | 1707.26±85.17 | 1381.25±105.59 | 3766.95±364.74 | 22566.24±1494.3 | 6098.88±447.81 | 5345.71±441.17 |
| 3-Methylbutyl pentanoate | 174.6±14.46 | 92.56±10.66 | 38.6±2.85 | 86.76±3.6 | 48.81±1.45 | 78.9±7.83 | 1046.56±62.63 | 488.94±41.96 | 437.93±8.46 | 232.63±11.72 |
| Ethyl 4-methylpentanoate | 228.87±7.87 | 583.29±46.74 | 181.15±9.04 | 624.98±23.12 | 156.08±12.65 | 207.46±7.61 | 231.83±12.99 | 605.38±36.59 | 94.48±9.21 | 345.76±13.49 |
| Ethyl hexanoate | 31313.06±154.46 | 27522.56±30.64 | 25905.88±55.09 | 30017.45±69.99 | 26249.4±41.44 | 25630.51±94.24 | 67977.58±260.99 | 49352.53±15.66 | 58531.58±222.01 | 36063.89±64.44 |
| Propyl hexanoate | 5.7±0.49 | 292.9±22.98 | 92.29±4.26 | 646.5±8.99 | 193.36±4.15 | 259.11±29.68 | 2161.31±201.53 | 4199.42±367.92 | 745.39±49.19 | 471.48±26.42 |
| Butyl hexanoate | 28.05±1.22 | 13.38±1.31 | 10.93±0.95 | 18.41±0.9 | 10.39±0.4 | 3.09±0.06 | 317.05±2.19 | 41.35±0.64 | 2.92±0.11 | 49.26±1.26 |
| 2-Methylpropyl hexanoate | 24.93±1.66 | 9.87±0.58 | 9.18±0.27 | 9.05±0.08 | 10.04±0.62 | 9.29±0.25 | 11.26±0.24 | 9.07±0.07 | 9.53±0.43 | 10.07±0.95 |
| 3-Methylbutyl hexanoate | 5354.7±222.43 | 5820.27±118.44 | 2808.39±51 | 4863.09±249.04 | 3137.81±110.03 | 5611.2±182.08 | 1233.36±20.91 | 2650.62±223.94 | 1316.11±97.22 | 4822.17±394.37 |
| Hexyl hexanoate | 375.7±4.52 | 179.68±16.69 | 110.74±9.25 | 367.61±39.88 | 119.16±7.98 | 152.39±13.64 | 5659.07±102.26 | 2347.05±222.59 | 2343.23±76.72 | 622.79±36.79 |
| 2-Phenethyl hexanoate | 2421.97±164.98 | 1364.61±128.33 | 877.41±57.98 | 1443.36±110.49 | 1112.88±58.33 | 1368.34±166.92 | 43569.56±188.94 | 11783.45±250.44 | 11903.49±149.68 | 3139.89±69.03 |
| Ethyl 5-methylhexanoate | 20.32±0.88 | 21.62±1.25 | 15.43±1.06 | 26.46±1.62 | 10.55±1.12 | 15.51±0.86 | 12.06±0.25 | 28.14±0.19 | 4.1±0.23 | 9.07±0.96 |
| Ethyl heptanoate | 585.21±40.36 | 288.28±10.23 | 105.35±3.79 | 536.28±8.02 | 64.91±3.61 | 128.44±15.64 | 2471.44±104.02 | 6610.34±503.19 | 735.87±85.7 | 758.34±27.26 |
| Ethyl octanoate | 1416.45±31.14 | 1560.53±28.02 | 928.98±21.07 | 571.23±70.63 | 776.19±54.76 | 1255.7±51.85 | 2636.9±254.45 | 10456.91±739.17 | 1236.94±23.82 | 1758.11±211.28 |
| Ethyl nonanoate | 582.12±21.72 | 487.43±30.74 | 350.23±21.84 | 518.07±61.41 | 301.06±17.34 | 444.52±30.33 | 680.58±64.11 | 2636.65±180.21 | 290.92±13.85 | 467.3±45.08 |
| Ethyl decanoate | 163.76±18.95 | 483.67±11.11 | 170.83±19.2 | 513.06±26.63 | 22.06±1.36 | 274.08±7.01 | 528.87±14.57 | 339.17±15.21 | 218.8±22.25 | 268.55±26.77 |
| (E)-Ethyl 4-decenoate | 108.91±5.65 | 281.06±1.9 | 67.19±3.94 | 191.59±6.46 | 80.13±0.72 | 40.81±2.94 | 46.94±3.33 | 30.69±2.38 | 0±0 | 49.83±4.02 |
| Ethyl undecanoate | 7.26±0.3 | 3.81±0.13 | 1.15±0.03 | 4.14±0.21 | 0±0 | 3.66±0.13 | 4.52±0.1 | 24.79±1.54 | 0±0 | 3.11±0.07 |
| Ethyl dodecanoate | 291.16±26.43 | 263.94±9.4 | 215.18±8.89 | 311.92±17.17 | 279.65±30.98 | 335.72±7.64 | 259.62±10.59 | 1153.24±74.88 | 177.15±13.49 | 240.37±20.56 |
| Ethyl tridecanoate | 56.37±3.83 | 56.11±2.27 | 42.42±4.24 | 49.65±1.89 | 40.41±3.55 | 50.79±3.92 | 39.36±2.42 | 121.77±8.67 | 26.5±1.3 | 38.87±3.86 |
| Ethyl tetradecanoate | 1227.71±90.05 | 969.89±61.46 | 635.46±27.22 | 1160.28±32.18 | 1314.12±27.7 | 1287.24±11.4 | 1510.68±34.93 | 5206.5±361.09 | 547.2±31.18 | 1169.81±94.85 |
| Ethyl pentadecanoate | 253.55±23.69 | 180.04±16.17 | 104.95±2.44 | 182.99±4.7 | 218.66±15.68 | 211.46±5.89 | 329.1±14.92 | 1018.64±88.59 | 113.82±4.22 | 236.26±19.17 |
| Ethyl hexadecanoate | 32435.16±1160.93 | 40799.44±1157.31 | 41161.85±1372.95 | 41683.98±1281.08 | 34185.14±469.37 | 35211.59±1193.59 | 28372.8±371.77 | 39700.18±584.86 | 33762.66±507.75 | 23314.27±151.29 |
| Ethyl 9-hexadecenoate | 2167.07±165.06 | 2589.24±215.13 | 2789.53±163.18 | 2523.72±153.99 | 2952.69±116.74 | 2881.36±90.57 | 1386.41±87.63 | 2526.75±8.91 | 2009.44±135.79 | 1237.29±47.11 |
| Ethyl heptadecanoate | 83.49±9.04 | 85.26±3.18 | 74.84±4.17 | 104.7±6.74 | 66.32±0.4 | 78.63±5.64 | 291.96±2.05 | 139.98±10.18 | 138.22±8.5 | 74.76±1.56 |
| Ethyl octadecanoate | 110.38±11.1 | 121.45±11.35 | 100.66±5.96 | 140.2±9.1 | 88.82±2.55 | 96.5±1.66 | 254.77±2.4 | 144.29±6.66 | 149.38±10.4 | 91.28±0.51 |
| Ethyl oleate | 9640.63±487.75 | 14420.17±505.78 | 15784.5±523.56 | 14310.18±486.54 | 11674.71±326.17 | 13233.28±556.61 | 8880.08±139.44 | 13455.63±246.14 | 12207.98±215.63 | 7213.99±180.15 |
| Ethyl linoleate | 17759.19±847.24 | 26816.87±850.54 | 30458.17±1331.15 | 25342.01±809.53 | 22633.82±596.83 | 25716.39±1047.19 | 14084.75±219.14 | 23553.62±551.66 | 17766.04±543.5 | 11400.45±249.86 |
| Ethyl linolenate | 527.55±19.28 | 452.67±6.44 | 382.01±19.98 | 500.21±58.85 | 371.69±3.02 | 521.48±62.6 | 601.43±47.39 | 650.04±59.17 | 596.3±15.71 | 481±28.3 |
| Diethyl butanedioate | 2089.43±71.97 | 2015.05±39.63 | 1683.45±209.28 | 1820.63±39.77 | 2349.35±29.78 | 1739.9±68.96 | 2145.88±72.71 | 873.93±56.75 | 983.1±63.52 | 2707.23±151.86 |
| Diethyl azeleate | 250.84±23.87 | 239.43±6.53 | 104.51±5.29 | 195.04±9 | 162.06±4.36 | 159.4±3.57 | 607.65±21.55 | 1575±96.16 | 238.77±25.42 | 441.34±13.75 |
| Ethyl lactate^a^ | 2115.68±120.43 | 2071.34±85.67 | 1963.17±91.49 | 1906.6±86.14 | 1978.73±55.33 | 2016.18±159.82 | 1480.19±34.82 | 1411±30.47 | 1278.22±35.9 | 1713.17±38.78 |
| 3-Methylbutyl lactate | 2206.28±196.17 | 2380.76±252.74 | 24306.03±1894.01 | 1786.7±109.41 | 351.1±20.85 | 1122.11±55.85 | 2163.28±34.15 | 9916.1±679.09 | 987.43±109.56 | 1999.39±70.25 |
| Ethyl 2-furancarboxylate | 126.66±8.64 | 122.82±14.64 | 156.95±5.49 | 211.9±10.97 | 237.83±15.76 | 249.2±12.53 | 115.97±1.66 | 115.72±11.66 | 332.34±13.16 | 193.08±7.8 |
| Ethyl 2-hydroxy-4-methylpentanoate | 13871.55±653.06 | 11277.17±759.68 | 10206.88±264.68 | 10949.11±614.47 | 10840.17±667.17 | 11530.03±335.87 | 10133.31±128.4 | 9604.34±165 | 7867.75±131.25 | 12627.37±37.42 |
| Ethyl 2-hydroxy-hexanoate | 38400.79±1443.65 | 25052.49±1400.05 | 18846.57±794.74 | 20627.01±2403.71 | 26722.83±305.63 | 28248.46±458.03 | 15519.6±1358.16 | 16300.51±1156.17 | 10573.36±664.38 | 28525.1±2507.59 |
| Ethyl benzoate | 14177.15±752.74 | 9081.55±1083.32 | 6512.05±528.88 | 9410.27±469.04 | 9124.31±171.37 | 12721.19±841.15 | 39940.25±440.5 | 19612.35±372.84 | 15316.91±993.57 | 14374.27±919.38 |
| Ethyl phenylacetate | 4467.4±255.76 | 5334.52±219.73 | 5420.06±231.87 | 4134.04±247.68 | 3780.33±128.06 | 4827.16±202.41 | 1811.94±27.59 | 1979.56±51.8 | 1359.29±114.68 | 2641.24±154.51 |
| Ethyl 3-phenylpropionate | 83.43±2.14 | 121.45±0.69 | 55.04±0.76 | 84.41±4.71 | 17.05±0.34 | 51.59±1.7 | 138.98±5.2 | 122.76±8.04 | 48.84±2.31 | 82.1±7.28 |
| Methanol^a^ | 174.71±0.25 | 175.91±0.86 | 179.29±0.57 | 180.32±0.29 | 187.36±0.5 | 158.14±2.28 | 169.14±0.38 | 166±0.26 | 146.38±0.09 | 183.39±0.42 |
| 1-Propanol^a^ | 1391.37±1.64 | 2988.02±0.69 | 4292.03±4.68 | 4195.65±10.42 | 6140.35±33.84 | 5348.34±107.28 | 1894.12±10.23 | 2154.43±2.66 | 3086.69±5.66 | 1862.24±2.81 |
| 1-Butanol | 111814.82±109.43 | 87551.08±53.37 | 69403.86±54.03 | 76966.02±106.42 | 69409.49±208.85 | 80550.54±1446.3 | 97028.72±455.05 | 85033.07±60.18 | 97743.23±44.79 | 135214.79±182.59 |
| 2-Butanol | 22263.68±99.02 | 61944.43±120.56 | 137833.99±10.59 | 116102.23±244.86 | 188203.56±993.36 | 180643.57±3456.95 | 67735.61±41.9 | 60092.97±112.51 | 72353.74±73.03 | 78502.89±138.07 |
| 2-Methylpropanol^a^ | 161.47±0.13 | 175.76±0.03 | 177.96±0.2 | 170.33±0.14 | 182.06±0.79 | 180.44±2.89 | 151.57±0.04 | 167.28±0.15 | 145.83±0.11 | 181.95±0.04 |
| 1-Pentanol | 9996.91±457.23 | 9404.85±320.22 | 8548.85±533.89 | 9176.1±363.6 | 9432.57±271.82 | 9017.46±405.44 | 12516.28±235.87 | 12090.89±186.52 | 10815.74±243.79 | 11748.8±172.36 |
| 2-Pentanol | 2098.93±128.57 | 2354.08±98.74 | 2162.26±22.25 | 2433.9±31.7 | 2225.47±88.29 | 2452.43±9.63 | 3728.62±32 | 2437.95±51.76 | 2256.49±3.79 | 2204.95±5.67 |
| 2-Methylbutanol | 46786.28±2520.9 | 45299.37±936.1 | 45299.12±1717.21 | 45862.15±4371.79 | 54095.92±1024.81 | 49999.57±1129.99 | 41180.41±2233.59 | 47878.64±3675.25 | 38115.31±1746.42 | 54542.76±158.42 |
| 3-Methylbutanol^a^ | 257.11±15.03 | 277.78±14.3 | 304.69±14.16 | 285.78±11.15 | 329.64±8.04 | 319.51±14.4 | 293.57±5.45 | 302.76±3.31 | 280.18±6.43 | 341.88±8.92 |
| 1-Hexanol | 5179.4±346.98 | 3881.19±83.9 | 3081.7±162.35 | 3332.76±218.55 | 3715.16±111.95 | 3076.97±182.87 | 15988.94±315.43 | 12159.48±263.59 | 11446.2±135.42 | 8589±453.78 |
| 1-Heptanol | 256.36±20.05 | 199.43±13.75 | 138.91±4.34 | 187.46±11.7 | 166.96±15.73 | 186.4±18.61 | 1093.64±30.87 | 237.61±22.92 | 230.64±27.61 | 423.68±2.74 |
| 2-Heptanol | 3628.27±207.9 | 3911.41±470.86 | 2373.43±35.07 | 3013.93±126.19 | 3285.01±79.34 | 4607.76±205.37 | 19479.58±803.34 | 1113.54±49.53 | 10592.75±1142.87 | 5404.09±48.15 |
| 1-Octanol | 517.52±41 | 433.37±11.07 | 299.91±11.41 | 343.12±7.36 | 428.37±13.26 | 358.59±36.24 | 628.13±37.53 | 15.11±0.75 | 333.25±33.8 | 655.72±69.82 |
| 1-Octen-3-ol | 284.66±19.43 | 342.22±17.28 | 271.03±31.04 | 264.14±7.93 | 308.79±15.23 | 227.84±22.02 | 660.37±59.4 | 357.74±37.35 | 383.41±15.2 | 319.47±3.46 |
| 3-Octanol | 155.52±4.43 | 116.47±12.44 | 55.54±4.81 | 134.71±7.58 | 58.94±5.27 | 77.13±1.82 | 91.73±7.63 | 295.25±9.94 | 52.6±2.96 | 83.73±4.88 |
| 1-Nonanol | 45.94±4.33 | 144.27±4.46 | 22.13±0.57 | 141.32±10.3 | 168.7±4.63 | 174.73±4.57 | 109.42±5.97 | 23.39±2.5 | 54.54±3.67 | 115.57±4.82 |
| 2-Nonanol | 50.9±1.17 | 41.18±0.97 | 26.63±0.48 | 27.29±0.91 | 10.65±0.76 | 51.87±1.55 | 19.09±2.15 | 122.75±6.31 | 5.84±0.37 | 14.3±0.96 |
| (E)-2-Nonen-1-ol | 335.23±9.33 | 194.95±10.78 | 629.24±10.72 | 628.65±16.47 | 198.96±7.57 | 1446.64±49.46 | 428.2±14.85 | 1044.05±75.73 | 693.95±29.76 | 840.09±77.31 |
| 1-Decanol | 1.53±0.17 | 1.22±0.14 | 0±0 | 0.94±0.03 | 0±0 | 0.49±0.02 | 3.67±0.27 | 10.31±0.55 | 0±0 | 0±0 |
| 2-Undecanol | 79.65±2.88 | 68.11±3.22 | 29.55±2.73 | 53.46±3.25 | 33.58±0.87 | 48.01±0.99 | 37.61±3.85 | 34.11±1.46 | 9.51±0.41 | 45.62±2.18 |
| 1,2-Propanediol | 30247.73±467.86 | 27307.16±611.16 | 28401.84±458.53 | 28646.5±260.45 | 23313.8±2112.63 | 27813.09±976.46 | 26606.99±194.42 | 30341.71±224.29 | 28993.75±324.23 | 30464.58±246.98 |
| (R,R)-2,3-Butanediol | 50154.28±1861.95 | 51050.07±1247.6 | 52896.61±2087.19 | 61398.87±2281.59 | 55785.65±5536.46 | 62633.98±3751.03 | 60506.42±709.42 | 59669.62±794.65 | 52002.75±858.75 | 63987.95±1078.7 |
| Meso-2,3-Butanediol | 13947.65±1270.62 | 11473.15±283.49 | 11587.66±410.78 | 13709.52±582.41 | 13340.75±659.59 | 15464.7±836.24 | 13456.45±15.63 | 11615.82±159.25 | 12055.69±92.11 | 13888.04±114.74 |
| Benzyl alcohol | 1032.66±67.74 | 405.88±17.45 | 3784.52±327.86 | 633.69±60.78 | 263.79±4.79 | 843.58±23.15 | 600.97±28.98 | 677.68±14.85 | 1161.32±48.55 | 591.17±37.73 |
| 2-Phenethyl alcohol | 10353.76±625.95 | 11099.57±430.1 | 11114.69±538.39 | 11255.42±565.75 | 13136.92±498.88 | 12887.25±1163.52 | 9468.07±226.06 | 9313.1±232.68 | 8343.95±267.29 | 10393.25±215.77 |
| Formic acid^a^ | 127.49±4.14 | 108.69±1.13 | 108.34±1.05 | 117.76±0.95 | 114.21±0.48 | 113.4±0.67 | 124.19±0.85 | 126.86±1.42 | 118.71±1.04 | 124.96±0.44 |
| Acetic acid^a^ | 1834.53±5.92 | 2182.96±4.59 | 1963.23±1.89 | 2047.73±2.75 | 1983.34±2.8 | 1927.1±1.92 | 1676.99±0.6 | 1812.1±16.59 | 2072.08±2.69 | 1678.36±0.95 |
| Propionic acid | 49703.31±915.1 | 58407.59±992.95 | 63256.91±1091.5 | 71040.71±1833.33 | 78826.45±2177.46 | 82549.6±2851.61 | 74392.06±6678.8 | 89629.07±7370.97 | 88483.67±5836.23 | 72331.68±1223.71 |
| Butanoic acid | 29172.29±46.06 | 39802.83±59.4 | 38577.74±99.53 | 32768.89±117.15 | 32279.6±63.92 | 34146.25±46.21 | 26133.88±91.75 | 28003.05±909.26 | 24047.55±77.63 | 29421.25±61.46 |
| 2-Methylpropanoic acid | 34341.25±105.72 | 28616.04±136.09 | 23470.2±49.03 | 27382.24±158.62 | 23883.76±140.52 | 26377.13±65.45 | 54643.2±10.59 | 43523.78±624.06 | 58042.27±118.15 | 65179.7±6.91 |
| Pentanoic acid | 92068.57±253.09 | 143127.5±1017 | 135650.84±737.61 | 112693.81±307.49 | 113504.79±475.98 | 120584.21±324.47 | 76332.95±33.3 | 84062.98±6719.72 | 72947.92±88.28 | 82323.32±270.72 |
| 3-Methylbutanoic acid | 18165.32±85.99 | 17455.44±122.51 | 16771.74±49.46 | 18107.97±52.31 | 17397.13±66.04 | 17182.6±38.71 | 26520.68±19.61 | 20589.07±2059.47 | 26352.88±37.84 | 20807.06±32.63 |
| Hexanoic acid | 16899.92±51.94 | 14906.1±32.55 | 13606.42±85.96 | 16247.21±66.08 | 13559.67±35.49 | 13581.41±59.15 | 44624.62±104.49 | 25879±1523.37 | 39043.07±300.82 | 20541.5±59.83 |
| Octanoic acid | 19321.18±144.96 | 19556.14±155.05 | 19408.22±163.66 | 19326.92±94.39 | 19280.58±125.56 | 19379.85±167.97 | 20472.35±37.79 | 19855.85±478.28 | 20384.69±2.35 | 19675.06±134.29 |
| Decanoic acid | 9043.75±101.69 | 9305.88±46.71 | 9280.31±142.43 | 9289.49±4.14 | 9259.66±99.28 | 9391.19±89 | 9065.31±45.54 | 9282.8±79.66 | 9262.99±54.41 | 9165.19±23.33 |
| Dodecanoic acid | 1537.7±71.6 | 3081.19±56.95 | 3041.98±81.18 | 2792.54±51.76 | 2044.77±37.73 | 2527.25±35.29 | 1555.8±28.57 | 2800.53±53.31 | 2565.46±28.23 | 1136.44±36.95 |
| Benzoic acid | 33999.97±3858.41 | 33065.37±2211.35 | 19818.03±1168.04 | 23631.76±1400.66 | 19734.58±177.88 | 36831.21±2630.2 | 42956.54±1280.85 | 51614.94±4414.94 | 44174.5±1485.68 | 29800.51±2572.22 |
| L-Lactic acid^a^ | 1736.32±4.32 | 1412.52±3.28 | 1676.85±3.98 | 1665.73±2.16 | 1526.11±0.31 | 1593.7±2.52 | 1329.15±2.19 | 1354.09±2.15 | 1183.94±1.56 | 1582.21±0.7 |
| Citric acid | 29761.23±83.23 | 24341±76.7 | 16530.63±105.42 | 24331.43±46.49 | 23069.7±54.16 | 27386.07±58.14 | 28303.73±80.53 | 31378.2±83.31 | 35385.27±11.35 | 25595.27±46.55 |
| Palmitic acid | 10775±36.74 | 8941.5±88.59 | 5610±160.85 | 10458.5±216.78 | 8427.5±220.05 | 10067.5±104.1 | 4383.5±58.38 | 17232.5±72.26 | 11509.5±53.48 | 9792.5±578.49 |
| Oleic acid | 8613±75.12 | 12532.5±146.56 | 13707.5±5.31 | 12160.5±80.42 | 10194.5±64.09 | 12313.5±93.49 | 6892.5±44.5 | 12583±119.21 | 8647.5±6.12 | 5626.5±11.84 |
| Linoleic acid | 3335±47.36 | 5146.5±40.42 | 5022±23.68 | 5333±80.83 | 3973.5±86.96 | 4929±162.48 | 3170.5±20 | 6082±39.19 | 4702.5±3.67 | 2381.5±25.72 |
| Acetaldehyde^a^ | 305.81±1.08 | 272.57±0.6 | 237.76±0.64 | 278.83±1.17 | 326.61±12.5 | 144.48±3.89 | 420.52±0.16 | 496.65±0.1 | 460.51±0.97 | 393.98±0.07 |
| 2-Methylpropanal | 5700.92±249.22 | 6773.44±74.33 | 8255.04±248.07 | 6837.28±147.54 | 10721.78±181.15 | 3480.09±67.65 | 5162.58±16.75 | 4737.52±144.01 | 4108.84±16.02 | 6853.38±173.41 |
| 2-Methylbutanal | 7077.55±83.1 | 8252.21±113.99 | 9707.62±146.74 | 8343.27±118.39 | 8412.74±297.41 | 3811.11±132.51 | 6493.53±105.48 | 5308.12±57.05 | 4447.91±125.51 | 8206.05±134.34 |
| 3-Methylbutanal | 37991.04±339.54 | 39876.64±517.2 | 45349.6±188.77 | 40895.85±474.09 | 40950.56±1045.62 | 28091.11±795.52 | 50757.09±200.16 | 46547.32±202.78 | 32735.1±129.91 | 50602.48±536.87 |
| Nonanal | 587.59±31.29 | 21.43±0.91 | 59.57±7.05 | 43.18±0.87 | 76.43±2.62 | 62.21±0.95 | 84.32±3.29 | 119.04±9.02 | 96.34±5.36 | 35.15±0.17 |
| Benzaldehyde | 3608.94±158.62 | 2136.49±128.57 | 2011.54±53.46 | 2064.41±104.64 | 2073.92±84.78 | 1910.76±134.41 | 3813.15±29.48 | 3942.58±100.17 | 2812.61±232.35 | 3348.94±11.35 |
| Benzeneacetaldehyde | 15033.22±1294.52 | 20083.88±464.46 | 17382.98±665.99 | 19885.28±1457.11 | 868.35±37.78 | 43149.1±1548.38 | 23922.22±1368.82 | 16209.15±1472.6 | 13599.09±962.61 | 10747.87±1215.18 |
| Diethoxymethane | 3941.94±115.12 | 2890.64±140.34 | 1453.07±62.58 | 2694.59±97.82 | 3655.91±97.53 | 721.28±19.1 | 5722.61±235.47 | 5783.42±83.38 | 7032.19±142.71 | 3377.52±149.67 |
| 1,1-Diethoxyethane | 236321.6±4660.68 | 209960.02±2834.29 | 166853.58±2166.87 | 216106.51±2736.03 | 226911.88±7519.47 | 91717.17±4419.03 | 333549.57±2767.11 | 396737.27±2727.39 | 361280.33±1723.27 | 308040.63±1698.76 |
| 1,1-Diethoxy-3-methylbutane | 25306.3±1287.33 | 26478.54±987.45 | 27523.45±1118.28 | 27135.63±1018.22 | 24762.39±1030.66 | 14578.43±950.23 | 33441.77±2274.55 | 31199.9±1290.54 | 20528.78±1249.67 | 33790.19±1735.43 |
| (2,2-Diethoxyethyl)-benzene | 55.42±1 | 258.76±8.56 | 58.48±0.93 | 218.06±14.75 | 21.71±0.76 | 35.35±2.43 | 66.25±1.39 | 26.42±0.52 | 18.69±0.39 | 119.13±10.2 |
| 1,1,3,3-Tetraethoxy-propane | 25.33±0.23 | 27.35±0.76 | 8.8±0.5 | 17.1±1.02 | 7.56±0.21 | 14.16±1.11 | 35.43±2.56 | 53.8±1.81 | 57.3±2.82 | 25.5±1.63 |
| Acetone | 25527.03±491.94 | 24173.43±156.65 | 23041.23±467.89 | 24858.22±288.02 | 21265.06±2133.73 | 10637.7±844.58 | 27809.44±108.42 | 27449.25±112.82 | 20610.13±18.83 | 30232.6±295.94 |
| 2-Pentanone | 6168.75±556.39 | 10718.56±153.6 | 7058.27±194.83 | 7336.05±219.76 | 7210.1±636.07 | 2827.34±82.68 | 13153.05±115.41 | 6253.1±294.27 | 6523.68±77.94 | 7842.55±485.94 |
| 3-Hydroxyl-2-butanone | 30122.82±1826.68 | 14127.75±696.69 | 10937.72±604.29 | 14598.19±776.42 | 19663.23±801.96 | 15801.89±1407.31 | 51273.74±1301.99 | 62275.33±1447.05 | 42021.48±1191.19 | 54433.83±1297.87 |
| 2-Heptanone | 40.93±1.8 | 49.32±3.32 | 50.6±2.13 | 34.94±1.56 | 838.49±15.15 | 1536.13±166.57 | 12.57±0.98 | 18.62±1.47 | 19.01±1.15 | 1374.32±18.04 |
| 2-Octanone | 2.37±0.27 | 2.88±0.32 | 0.43±0.02 | 1.95±0.14 | 0.31±0.02 | 1.08±0.07 | 3.37±0.07 | 0.82±0.02 | 0.7±0.07 | 0.1±0 |
| 3-Octanone | 37.92±1.61 | 74.35±5.33 | 56.39±0.39 | 26.31±2.51 | 36.17±2.86 | 72.16±1.71 | 184.14±5.86 | 43.31±2.94 | 95.05±3.71 | 42.33±1.62 |
| 2-Nonanone | 180.73±18.83 | 197.57±7.05 | 95.97±1.83 | 170.33±8.83 | 92.27±3.06 | 0±0 | 99.14±1.47 | 352.54±24.23 | 22.83±1.54 | 117.11±10.5 |
| 4-Nonanone | 128.55±13.32 | 93.11±2.5 | 51.1±6.05 | 48.95±2 | 47.01±3.18 | 64.71±2.23 | 226.02±7.59 | 54.64±0.76 | 42.69±1.04 | 82.31±5.94 |
| 2-Undecanone | 85.57±4.56 | 65.14±7.99 | 36.9±2.1 | 52.76±5.46 | 21.54±1.75 | 48.83±3.97 | 44.7±5.1 | 155.38±3.37 | 18.79±1.4 | 47.41±5.28 |
| 4-Undecanone | 218.51±12.23 | 1185.45±15.66 | 1227.14±61.26 | 429.92±14.09 | 325.44±10.31 | 435.96±21.22 | 644.95±27.24 | 2179.42±16.55 | 33.44±1.94 | 1041.56±31.69 |
| 2-Tridecanone | 52.03±4 | 56.49±6.87 | 30.67±2.56 | 41.45±1.64 | 13.62±1.15 | 0±0 | 22.47±0.76 | 65.71±2.26 | 1.65±0.1 | 17.57±1.04 |
| 2-Pentadecanone | 105.63±7.84 | 79.21±4.6 | 45.05±2.66 | 63.92±2.7 | 66.71±1.82 | 71.68±3.38 | 61.4±1.62 | 186.69±14.99 | 27.2±1.54 | 52.46±4.76 |
| Furfuryl alcohol | 25727.16±513.06 | 23702.53±277.06 | 23130.89±1860.35 | 22998.41±2530.57 | 21955.55±2359.6 | 25315.59±700.32 | 12759.91±223.86 | 10061.57±168.8 | 10813.73±219.41 | 19016.02±322.12 |
| Furfural^a^ | 227.94±8.02 | 199.97±8.61 | 210.22±6.28 | 184.45±6.92 | 198.02±3.12 | 207.09±14.56 | 254.3±3.55 | 226.58±4.94 | 182.69±1.59 | 266.75±2.87 |
| 5-Methylfurfural | 760.55±12.93 | 513.53±16.82 | 536.88±9.88 | 599.37±25.28 | 597.38±35.57 | 628.87±23.17 | 1935.11±98.11 | 947.18±72.66 | 771.01±66.07 | 1093.7±18.81 |
| Furfuryl ethyl ether | 11896.15±289.97 | 10597.67±173.78 | 11577.13±191.2 | 9871.03±243.32 | 10163.18±279.06 | 10191.99±264.98 | 8247.95±118.77 | 5589.79±140.84 | 5430.12±57.29 | 11107.34±171.6 |
| 2-Acetylfuran | 1739.17±110.52 | 1598.03±80.28 | 1164.2±61.57 | 1410.54±111.06 | 1686.83±45.79 | 1684.11±86.37 | 3380.98±135.81 | 1874.58±78.83 | 1191.67±122.09 | 2054.3±32.9 |
| HDMF | 33±0.69 | 28.92±1.29 | 47.81±1.28 | 36.65±1.14 | 59.76±2.18 | 60.07±1.08 | 52.21±0.38 | 50.79±0.36 | 67±1.33 | 60.91±2.71 |
| HEMF | 6.98±0.74 | 2.91±0.12 | 0.72±0.1 | 0±0 | 0±0 | 1.43±0.15 | 0±0 | 0±0 | 0±0 | 12.83±0.57 |
| Sotolon | 27.94±0.82 | 16.92±0.64 | 18.25±0.21 | 21.62±1.61 | 19.42±0.82 | 14.2±0.58 | 43.68±1.12 | 38.14±0.81 | 30.03±0.46 | 27.34±1.66 |
| Pyrazine | 62.6±1.73 | 53.78±1.33 | 23.5±0.49 | 19.47±0.9 | 45.05±0.9 | 26.38±0.54 | 60.38±1.45 | 67.83±0.46 | 0.45±0 | 60.06±0.74 |
| 2-Methylpyrazine | 515.9±17.04 | 530.89±3.75 | 228.35±3.52 | 171.88±2.09 | 644.27±4.11 | 256.8±2.04 | 956.2±7.79 | 1012.15±3.82 | 211.16±3.61 | 731.42±6.4 |
| 2,3-Dimethylpyrazine | 344.99±1.21 | 454.99±15.37 | 223.66±2 | 200.93±1.67 | 939.52±24.1 | 494.39±4.68 | 558.03±5 | 619.33±0.86 | 141.38±1.61 | 644.22±2.54 |
| 2,5-Dimethylpyrazine | 2569.94±83.45 | 2979.47±53.18 | 1126.3±5.05 | 1075.5±4.83 | 2802.07±37.23 | 1860.65±4.34 | 5300.02±41.11 | 5308.45±121.8 | 1274.13±2.79 | 4723.85±101.55 |
| 2,6-Dimethylpyrazine | 534.83±6.89 | 537.87±10.97 | 261.02±12.61 | 174.34±8.97 | 616.58±5.38 | 394.13±5.24 | 957.34±6.03 | 1296.21±9.25 | 216.33±0.85 | 963.47±3.88 |
| 2-Ethylpyrazine | 99.4±1.4 | 101.04±0.49 | 0±0 | 0±0 | 47.03±1.87 | 0±0 | 276.04±2.59 | 275.83±2.85 | 0±0 | 247.31±3.25 |
| 2-Ethyl-3-methylpyrazine | 92.51±0.99 | 90.42±2.87 | 8.15±0.98 | 0±0 | 78.09±0.5 | 28.45±1.08 | 144.49±1.1 | 167.99±1.39 | 25.23±0.34 | 127.02±1.49 |
| 2-Ethyl-6-methylpyrazine | 1802.58±36.77 | 1727.47±14.74 | 214.38±4.76 | 250.54±5.01 | 1385.03±59.49 | 489.67±1.43 | 4391.93±81.29 | 3605.27±93.86 | 710.38±5.79 | 3700.47±120.17 |
| 2,3,5-Trimethylpyrazine | 5140.43±98.9 | 7049.49±64.27 | 3753.4±8.19 | 3526.31±16.77 | 8238.23±82.73 | 5523.77±35.22 | 7518.07±40.36 | 7514.04±75.85 | 1858.48±19.11 | 9604.45±105 |
| 2-Ethyl-3,5-dimethylpyrazine | 2411.04±54.12 | 3061.56±55.73 | 1064.79±18.34 | 1040.5±9.95 | 3519.18±34.04 | 1839.63±15.62 | 7653.14±316.08 | 6193.97±335.12 | 1998.24±15.68 | 6475.45±290.67 |
| 2-Ethyl-3,6-dimethylpyrazine | 271.32±0.48 | 279.16±0.56 | 105.31±1.35 | 101.57±1.06 | 263.3±4.14 | 222.22±1.97 | 425.63±3.22 | 418.11±2.86 | 171.19±2.69 | 430.57±3.25 |
| 2,3,5,6-Tetramethylpyrazine | 14170.94±60.79 | 24242.76±54.93 | 13412.01±55.22 | 13516.25±55.03 | 36930.33±813.72 | 19275.26±108.69 | 11536.56±141.9 | 13934.27±166.75 | 3139.51±45.5 | 24999.12±254.83 |
| 2,3-Diethylpyrazine | 27.94±0.89 | 6.68±0.19 | 0±0 | 0±0 | 29.45±8.46 | 0±0 | 56.16±4.18 | 66.94±8.92 | 62.54±1.84 | 59.48±0.85 |
| 2,3-Diethyl-5-methylpyrazine | 116.98±8.79 | 56.79±3.59 | 22.06±2.08 | 24.63±2.38 | 73.47±2.24 | 49.51±2.21 | 106.77±2.94 | 126.21±0.14 | 26.2±1.89 | 126.79±3.78 |
| 2-Isobutyl-3-methylpyrazine | 156.89±10.32 | 146.16±1.49 | 49.53±2.7 | 41.22±0.98 | 153.44±4.77 | 103.02±2.12 | 199.01±1.59 | 193.02±2.51 | 62.99±2.55 | 187±2.5 |
| (Trimethylpyrazin-2-yl)methanol | 51.04±0.26 | 125.91±0.72 | 53.19±0.33 | 67.62±0.16 | 238.36±7.71 | 106.75±1.82 | 37.03±0.07 | 43.9±0.29 | 4.64±0.14 | 83.54±0.15 |
| Dimethyl trisulfide | 230.78±16.09 | 253.74±25.01 | 320.19±14.86 | 288.75±20.93 | 231.33±1.36 | 364.45±26.77 | 628.7±19.13 | 298.61±11.2 | 266.96±27.98 | 246.42±29 |
| Phenol | 1183.31±88.34 | 1159.78±105.78 | 880.81±53.8 | 938.88±91.01 | 803.4±63.91 | 976.15±10.21 | 3401.81±17.45 | 2299.64±79.55 | 1333.25±95.63 | 1430.32±2.16 |
| p-Cresol | 280.53±12.02 | 265.61±13.57 | 185.21±13.38 | 214.63±5.27 | 207.25±7.32 | 48.76±5.08 | 1299.94±14.31 | 552.43±18.46 | 469.47±37.8 | 429.3±12.11 |
| Guaiacol | 682.99±43.67 | 369.58±23.86 | 219.75±2.33 | 633.68±39.42 | 161.21±11.56 | 285.15±34.58 | 4738.87±338.05 | 1033.54±12.68 | 1124.77±58.38 | 579.87±6.07 |
| 4-Methylguaiacol | 112.6±8.44 | 107.59±8.55 | 87.95±10.17 | 56.44±1.4 | 41.98±0.49 | 82.17±9.16 | 81.26±6.02 | 40.07±2.59 | 42.27±0.81 | 30.68±1.76 |
| 4-Ethylguaiacol | 681.43±7.71 | 700.58±9.81 | 780.7±66.43 | 325.54±12.73 | 375.99±17.99 | 815.62±68.95 | 1647.13±22.51 | 723.7±36.48 | 223.57±9.79 | 572.06±20.36 |
| 4-Vinylguaiacol | 10.11±0.6 | 9.85±0.84 | 20.43±1.02 | 4.82±0.18 | 4.58±0.36 | 5.6±0.61 | 49.71±1.79 | 5.05±0.13 | 3.34±0.11 | 3.07±0.24 |
| gama-Nonalactone | 417.62±27.44 | 411.44±22.56 | 267.74±14.66 | 265.65±11.26 | 195.59±11.3 | 458.03±29.49 | 760.89±3.34 | 346.89±1.85 | 253.17±20.04 | 295.42±2.31 |
| gama-Dodecalactone | 511.74±27.95 | 456.42±18.73 | 392.28±15.8 | 423.32±21.19 | 357.77±15.26 | 542.07±57.6 | 532.61±19.75 | 805.01±5.26 | 625.32±39.06 | 511.62±1.89 |
| Phytone | 74.53±6.69 | 51.17±4.25 | 30.08±0.45 | 49.07±3 | 71.27±1.9 | 70.26±7.93 | 94.73±3.96 | 231.69±21.68 | 22.8±1.82 | 56.98±5.34 |
| beta-Damascone | 18.5±0.59 | 21.69±1.77 | 21.41±2.46 | 13.76±0.52 | 9.28±1.02 | 10.81±0.66 | 25.81±1.51 | 12.89±1.03 | 9.31±0.04 | 11.59±0.02 |
| Geranylacetone | 50.92±5.54 | 66.59±2.3 | 36.55±2.34 | 64.92±0.95 | 49.62±1.1 | 47.87±1.78 | 41.86±3.94 | 83.19±1.84 | 6.67±0.21 | 35.85±2.68 |

Data represent mean ± SD (n = 3).

^a^The unit of compound is mg/L.

Continue Table S3 The average content of 154 flavor compounds in MT towns samples (MT-11 to MT-20) (μg/L)

| Name | MT-10 | MT-11 | MT-12 | MT-13 | MT-14 | MT-15 | MT-16 | MT-17 | MT-18 | MT-19 | MT-20 |
| --- | --- | --- | --- | --- | --- | --- | --- | --- | --- | --- | --- |
| Ethyl formate | 38830.78±893.31 | 34818.51±288.76 | 52372.47±481.78 | 79371.48±1447.64 | 21937.72±185.96 | 34517.92±636.72 | 30203.91±225.28 | 43430.32±414.42 | 86283.46±1290.21 | 112683.41±1527.78 | 148060.22±1021.72 |
| Ethyl acetate^a^ | 2478.53±48.99 | 2199.64±21.34 | 2681.61±25.8 | 2354.95±50.9 | 2143.93±46.15 | 2313.69±52.42 | 2307.53±82.61 | 2057.34±96.13 | 2359.94±26.24 | 2591.81±78.37 | 2782.38±85.65 |
| Propyl acetate | 12660.15±861.65 | 11541.12±710.75 | 10138.88±285.58 | 2086.48±50.76 | 2915.06±144.89 | 3495.97±283.88 | 6393.63±501.75 | 5912.87±209.62 | 8225.8±105.56 | 9610.98±295.52 | 1617.67±194.56 |
| 3-Methylbutyl acetate | 3732.26±82.63 | 3588.91±22.73 | 3693.75±52.04 | 3590.72±42.92 | 4304.55±100.17 | 3438.01±82.16 | 3715.58±28.81 | 2682.12±25.36 | 3618.72±84.5 | 3880.46±91.35 | 3775.37±106.5 |
| Hexyl acetate | 297.34±8.54 | 308.46±31.36 | 303.86±15.09 | 177.11±4.84 | 165.31±12.2 | 338.82±39.6 | 336.32±31.88 | 186.78±3.48 | 335.65±4.99 | 183.38±17.65 | 330.73±12.56 |
| 2-Phenylethyl acetate | 108.16±9.69 | 80.41±8.09 | 55.72±0.7 | 29.17±0.73 | 145.25±5.37 | 58.73±5.09 | 154.2±7.65 | 0±0 | 12.49±1.09 | 0±0 | 17.63±0.83 |
| Ethyl propanoate | 62280.59±996.65 | 56933.7±713.8 | 51886.32±2410.38 | 49813.88±62.11 | 18940.94±562.47 | 39101.67±1452.97 | 39058.78±1044.74 | 122993.95±1298.52 | 78839.98±1442.39 | 101449.86±1506.45 | 48493.28±299.26 |
| Ethyl 2-methylpropanoate | 15581.21±479.59 | 14761.27±250.9 | 16598.02±191.85 | 17565.53±723.18 | 2839.41±91.72 | 12665.47±173.45 | 13180.48±294.61 | 14129.33±460.31 | 15889.86±608.77 | 19590.24±379.39 | 20469.47±274.83 |
| Ethyl butanoate | 89888.63±1674.83 | 83134.38±1543.06 | 79877.91±1392.11 | 81920.15±1831.82 | 25499.02±456.39 | 99880.39±1931.51 | 94807.7±1795.56 | 53162.92±1041.6 | 86706.49±1792.49 | 85931.36±1532.78 | 76876.4±1083.69 |
| Butyl butanoate | 148.42±9.5 | 240.86±22.77 | 59.98±1.77 | 74.09±0.61 | 299.84±24.22 | 29.83±2.1 | 133.24±11.18 | 8.45±0.34 | 31.74±2.37 | 17.36±1.5 | 30.35±3.41 |
| 3-Methylbutyl butanoate | 537.67±8.57 | 531.54±63.89 | 526.03±31.25 | 589.57±61.87 | 217.23±23.51 | 616.32±59.71 | 520.29±64.82 | 197.94±9.81 | 468.68±28.07 | 23.85±0.46 | 47.16±2.57 |
| 2-Phenethyl buanoate | 802.61±6.54 | 516.45±16.99 | 512.06±52.15 | 329.89±18.02 | 1161.55±109.45 | 820.65±1.98 | 666.64±19.18 | 142.75±3.92 | 421.49±33.6 | 228.01±23.6 | 209.48±8.53 |
| Ethyl 2-methylbutanoate | 2389.63±80.64 | 2891.87±72.66 | 3301.23±242.56 | 5054.93±221.07 | 0±0 | 2304.45±129.5 | 2074.35±118.05 | 3755.81±316.72 | 4427.03±325.92 | 7068.65±396.87 | 8202.43±158.32 |
| Ethyl 3-methylbutanoate | 8503.35±243.67 | 8516.05±216.35 | 10673.83±249.08 | 15757.96±301.79 | 1636.56±117.99 | 7894.79±134.62 | 7586.33±156.49 | 11306.19±183.46 | 13918.98±449.54 | 20259.72±340.05 | 24229.77±408.45 |
| Ethyl pentanoate | 5345.71±441.17 | 7865.23±204.79 | 11554.9±611.64 | 14018.78±1080.26 | 3821.73±32.93 | 6776.69±682.46 | 14782±837.16 | 7600.75±159.05 | 6772.33±291.26 | 4779.37±46.03 | 7318.85±349.46 |
| 3-Methylbutyl pentanoate | 232.63±11.72 | 152.22±17.84 | 212±2.31 | 155.99±13.2 | 1210.34±65.56 | 207.75±8.98 | 312.73±3.56 | 175.71±2.07 | 169.35±16.06 | 108.64±3.01 | 234.81±3.9 |
| Ethyl 4-methylpentanoate | 345.76±13.49 | 314.26±14.37 | 447.6±35.97 | 446.31±39.5 | 179.28±16.97 | 91.02±2.07 | 243.48±14.23 | 248.09±11.68 | 256.87±22.46 | 89.58±1.72 | 145±1.5 |
| Ethyl hexanoate | 36063.89±64.44 | 35605.5±79.43 | 35613.07±37.35 | 31457.95±33.72 | 32662.03±12.26 | 59479.38±198.19 | 51603.37±23.66 | 45994.19±123.46 | 45092.38±92.67 | 39326.36±32.1 | 35910.74±34.85 |
| Propyl hexanoate | 471.48±26.42 | 557.66±27.09 | 480.68±31.76 | 171.98±13.76 | 508.42±38.3 | 832.9±30.19 | 1083.39±100.11 | 863.23±46.16 | 643.57±11.7 | 401.08±30.99 | 171.41±19.03 |
| Butyl hexanoate | 49.26±1.26 | 23.84±1.18 | 2.63±0.02 | 21.91±0.21 | 71.9±0.87 | 75.65±6.45 | 2.78±0.02 | 27.74±0.09 | 51.46±4.01 | 34.32±2.69 | 2.8±0.17 |
| 2-Methylpropyl hexanoate | 10.07±0.95 | 9.1±0.01 | 9.54±0.1 | 9.45±0.06 | 53.7±5.12 | 8.9±0 | 9.38±0.04 | 14.53±1.43 | 10.48±0.65 | 25.11±2.18 | 9.17±0.07 |
| 3-Methylbutyl hexanoate | 4822.17±394.37 | 1838.09±146.52 | 5853.41±517.58 | 2000.83±175.31 | 947.83±103.51 | 2253.54±63.09 | 1351.92±65.13 | 300.11±19.63 | 650.78±31.43 | 563.17±23.38 | 1163.54±127.7 |
| Hexyl hexanoate | 622.79±36.79 | 515.87±34.1 | 448.59±33.91 | 210.5±19.38 | 8796.23±573.16 | 1854.07±160.57 | 778.15±29.44 | 152.43±1.57 | 769.53±12.62 | 244.22±26.87 | 164.91±19.96 |
| 2-Phenethyl hexanoate | 3139.89±69.03 | 2022.1±68.31 | 2549.48±276.89 | 1201.13±47.37 | 7517.33±49.37 | 6517.32±79.4 | 5071.54±201.49 | 1294.9±43.44 | 4018.53±168.32 | 1668.94±130.15 | 1181.96±29.13 |
| Ethyl 5-methylhexanoate | 9.07±0.96 | 9.55±0.22 | 12.43±0.54 | 18.16±0.29 | 6.79±0.48 | 12.71±1 | 11.61±1 | 13.46±0.88 | 9.58±0.95 | 6.32±0.49 | 8.57±0.42 |
| Ethyl heptanoate | 758.34±27.26 | 961.72±89.27 | 1258.58±133.4 | 1096.38±73.39 | 904.77±52.64 | 1997.53±153.43 | 2226.77±251.76 | 1112.15±82.82 | 1123.67±112.73 | 841.58±33.57 | 1003.17±15.73 |
| Ethyl octanoate | 1758.11±211.28 | 1739.35±131.88 | 2419.92±165.36 | 2021.27±210.55 | 2044.05±245.93 | 3837.73±161.9 | 2674.91±214.62 | 1625.26±72.2 | 1937.07±42.55 | 623.56±31.09 | 2591.62±67.6 |
| Ethyl nonanoate | 467.3±45.08 | 531.83±26.52 | 557.18±62.14 | 529.3±12.78 | 914.47±36.76 | 625.3±9.91 | 569.11±51.85 | 445.87±14.58 | 542.96±31.34 | 31.14±0.2 | 847.26±25.64 |
| Ethyl decanoate | 268.55±26.77 | 419.8±31.69 | 823.77±80.04 | 596.34±24.25 | 268.77±33.51 | 407.93±41.7 | 385.02±14.65 | 347.03±5.69 | 289.71±4.69 | 93.69±4.14 | 178.42±12.37 |
| (E)-Ethyl 4-decenoate | 49.83±4.02 | 70.13±5.23 | 30.07±1.21 | 210.61±25.15 | 164.93±16.92 | 63.32±6.18 | 150.21±14.57 | 58.55±7.17 | 40.49±2.96 | 9.46±0.93 | 39.07±2.4 |
| Ethyl undecanoate | 3.11±0.07 | 1.99±0.18 | 5.81±0.24 | 12.35±0.82 | 18.76±0.58 | 3.5±0.19 | 4.27±0.15 | 2.41±0.26 | 3.24±0.32 | 1.44±0.12 | 9.42±0.89 |
| Ethyl dodecanoate | 240.37±20.56 | 284.34±24.17 | 386.26±25.16 | 377.48±31.39 | 669.87±32.04 | 626.77±56.16 | 356.26±7.6 | 228.98±4.19 | 315.16±22.29 | 226.96±13.61 | 505.24±32.4 |
| Ethyl tridecanoate | 38.87±3.86 | 45.04±1.2 | 56.44±3.22 | 55.09±2.71 | 55.37±0.92 | 48.13±3.99 | 50.92±1.43 | 37.39±1.37 | 42.84±3.35 | 30.46±0.53 | 52.85±0.29 |
| Ethyl tetradecanoate | 1169.81±94.85 | 1413.8±110.27 | 1542.77±126.5 | 1817.39±56.4 | 1552.45±131.44 | 1311.28±66.85 | 1273.02±72.32 | 1166.04±54.95 | 1240.09±125.96 | 860.64±65.27 | 2225.35±150.95 |
| Ethyl pentadecanoate | 236.26±19.17 | 273.58±20.37 | 264.89±27.12 | 358.84±2.64 | 195.2±16.83 | 259.25±8.58 | 276.66±18.46 | 285.6±17.66 | 282.79±26.06 | 158.22±12.56 | 438.28±34.95 |
| Ethyl hexadecanoate | 23314.27±151.29 | 22094.79±227.54 | 28024.6±307.44 | 27357.36±363.59 | 30178.32±389.32 | 22474.45±249.46 | 29834.26±387.47 | 22408.28±142.97 | 42885.41±715.51 | 30582.57±402.23 | 39075.3±410.53 |
| Ethyl 9-hexadecenoate | 1237.29±47.11 | 1336.72±51.89 | 1897.67±6.13 | 1646.17±42.62 | 1915.38±109.32 | 1293.15±88.17 | 1590.24±52.53 | 653.31±47.84 | 2199.11±40.21 | 1697.1±105.75 | 1769.39±2.48 |
| Ethyl heptadecanoate | 74.76±1.56 | 61.9±0.05 | 26.55±2.07 | 29.87±0.35 | 205.64±10.06 | 73.91±0.14 | 97.14±0.05 | 87.67±0.81 | 151.33±1.19 | 72.73±1.16 | 144.89±9.58 |
| Ethyl octadecanoate | 91.28±0.51 | 79.07±0.8 | 78.54±2.65 | 78.29±0.49 | 541.54±6.28 | 84.8±1.11 | 112.39±3.36 | 100.25±1 | 150.47±1.95 | 87.6±1.01 | 145.22±9.52 |
| Ethyl oleate | 7213.99±180.15 | 6743.08±141.7 | 9570.73±135.44 | 9925.42±182.17 | 12981.05±204.06 | 8048.97±175.21 | 11084.66±127.05 | 6202.99±65.61 | 13599.25±152.53 | 11102.28±19.43 | 12506.99±244.04 |
| Ethyl linoleate | 11400.45±249.86 | 11428.01±444.37 | 16015.19±403.75 | 15100.66±399.03 | 20306.65±387.8 | 13377.76±318.73 | 16922.27±422.53 | 8249.11±188.33 | 19209.39±383.26 | 13060.04±43.45 | 9916.76±290.93 |
| Ethyl linolenate | 481±28.3 | 429.96±6.25 | 443.73±44.36 | 303.64±2.59 | 1078.74±93.47 | 860.94±2.21 | 581.51±5.66 | 286.35±7.2 | 415.94±0.51 | 196.74±18.08 | 95.76±10.93 |
| Diethyl butanedioate | 2707.23±151.86 | 2358.72±71.83 | 1978.91±206.28 | 2533±236.55 | 1119.45±64.53 | 1610.73±156.22 | 1378.63±50.86 | 1583.01±61.83 | 2959.1±94.6 | 1094.08±24.11 | 2589.99±169.82 |
| Diethyl azeleate | 441.34±13.75 | 522.57±31.82 | 535.78±44.46 | 1013.45±111.28 | 243.69±22.61 | 407.57±11.43 | 304.2±20.6 | 785.75±36.41 | 852.67±67.11 | 697.99±14.57 | 1960.58±237.54 |
| Ethyl lactate^a^ | 1713.17±38.78 | 1718.23±41.9 | 1577.95±40.04 | 1443.83±39.47 | 1147.37±25.76 | 1300.7±27.85 | 1258.04±27.02 | 957.65±19.02 | 1149.55±25.09 | 1436.28±31.23 | 1599.39±27.11 |
| 3-Methylbutyl lactate | 1999.39±70.25 | 2060.26±186.57 | 1660.62±186.06 | 1299.24±121.04 | 1569.58±43.1 | 595.22±52.82 | 1080.46±68.49 | 359.86±22.33 | 1640.61±63.31 | 1450.89±122.7 | 2492.46±94.01 |
| Ethyl 2-furancarboxylate | 193.08±7.8 | 193.25±12.54 | 271.56±13.88 | 887.17±32.15 | 71.87±3.84 | 146.49±17.91 | 170.11±7.92 | 365.86±15.67 | 1027.5±15.95 | 1480.29±79.58 | 5495.28±270.7 |
| Ethyl 2-hydroxy-4-methylpentanoate | 12627.37±37.42 | 12833.79±35.77 | 11078.18±181.27 | 11102.76±43.97 | 4960.73±32.26 | 6777.75±72.5 | 6443±260.54 | 7285.37±298.21 | 9663.94±249.54 | 13179.39±115.32 | 9811.14±370.07 |
| Ethyl 2-hydroxy-hexanoate | 28525.1±2507.59 | 22147.87±1998.65 | 19234.65±1063.59 | 16006.09±1073.85 | 8714.13±240.66 | 15002.62±1379.57 | 10656.83±814.56 | 11003.54±334.85 | 16761.88±1203.13 | 12252.37±407.02 | 27617.76±527.88 |
| Ethyl benzoate | 14374.27±919.38 | 9311.08±1003.34 | 15720.38±1764.11 | 14265.88±326.06 | 5597.08±280.85 | 14305.06±78.16 | 9809.84±525.42 | 8290.64±76.43 | 20493.56±1825.62 | 17218.32±1368.87 | 22227.45±1460.24 |
| Ethyl phenylacetate | 2641.24±154.51 | 2513.26±242.76 | 3045.35±151.97 | 3183.82±70.11 | 915.06±35.63 | 2233.06±184.51 | 3322.8±78.55 | 1560±44.86 | 2806.63±91.58 | 2187.52±31.93 | 2350.45±33.83 |
| Ethyl 3-phenylpropionate | 82.1±7.28 | 109.01±5.07 | 89.43±2.15 | 124.11±9.02 | 71.84±2.23 | 111.64±13.34 | 121.82±5.17 | 85.95±2.04 | 57.31±5.29 | 31.12±1.27 | 56.67±2.97 |
| Methanol^a^ | 183.39±0.42 | 173.61±0.2 | 184.58±0.4 | 183.73±1.15 | 108.33±0.09 | 137.16±0.03 | 144.27±0.46 | 136.32±0.09 | 196±0.2 | 203.63±0.09 | 200.18±0.53 |
| 1-Propanol^a^ | 1862.24±2.81 | 1835.88±4.69 | 1420.12±5.24 | 688.18±0.77 | 806.79±0.16 | 967.11±0.91 | 1339.51±2.45 | 1441.29±1.73 | 1514.37±0.04 | 1944.81±1.89 | 594.47±0.18 |
| 1-Butanol | 135214.79±182.59 | 130534.72±139.51 | 122574.58±172.32 | 89474.86±109.15 | 57078.51±36.26 | 123020.59±110.09 | 125187.73±31.64 | 69999.05±10.37 | 92619.46±150.66 | 92727.01±53.22 | 93501.33±183.63 |
| 2-Butanol | 78502.89±138.07 | 75864.84±104.82 | 51370.98±64.77 | 27167.96±26.41 | 51276.29±93.22 | 30249.85±86.19 | 83653.24±3.21 | 56048.59±98.76 | 47363.8±111.62 | 60526.1±150.29 | 27511.87±24.77 |
| 2-Methylpropanol^a^ | 181.95±0.04 | 177.22±0.05 | 185.42±0.2 | 181.47±0.18 | 92.95±0 | 172.52±0.03 | 162.54±0.18 | 138.19±0.09 | 197.22±0.38 | 186.54±0.07 | 195.92±0.07 |
| 1-Pentanol | 11748.8±172.36 | 11795.3±133.72 | 11246.86±272.48 | 11551.41±268.25 | 7925.44±196.46 | 9194.55±159.46 | 9381.05±220.59 | 9148.64±141.78 | 12812.94±239.95 | 11760.84±224.86 | 11714.83±243.26 |
| 2-Pentanol | 2204.95±5.67 | 2105.38±142.75 | 2222.27±8.94 | 1261.28±23.19 | 372.96±5.15 | 1498.29±58.43 | 1582.83±61.43 | 1862.42±82.23 | 2604.49±61.41 | 1462.21±21.27 | 1103.14±6.62 |
| 2-Methylbutanol | 54542.76±158.42 | 53714.58±60.11 | 52580.64±2862.3 | 59769.62±415.1 | 20632.79±486.07 | 50961.85±1071.32 | 33926.77±461.75 | 30913.13±1248.02 | 63284.63±1189.05 | 49820.74±2433.8 | 55446.66±3435.05 |
| 3-Methylbutanol^a^ | 341.88±8.92 | 335.84±8.61 | 337.94±6.37 | 381.24±10.73 | 229.37±5.12 | 359.32±10.38 | 318.02±7.62 | 239.82±4.03 | 375.92±8.2 | 361.33±6.14 | 382.12±4.07 |
| 1-Hexanol | 8589±453.78 | 8210.29±150 | 8006.94±45.48 | 5223.91±45.5 | 2726.78±144.24 | 8261.55±28.25 | 8286.36±27.13 | 6496.24±147.35 | 14005.43±293.72 | 8361.67±413.82 | 8767.94±230.27 |
| 1-Heptanol | 423.68±2.74 | 246.52±6.08 | 254.45±8.57 | 224.78±9.9 | 440.48±28.21 | 196.45±10.72 | 146.1±2.72 | 218.31±7.12 | 331.54±3.89 | 155.77±12.4 | 253.17±22.4 |
| 2-Heptanol | 5404.09±48.15 | 1707.31±127.03 | 5216.13±432.44 | 3044.55±119.01 | 4336.25±243.11 | 2521.17±244.33 | 1541.25±133.84 | 2648.71±101.14 | 3164.11±147.01 | 2414.93±2.34 | 2248.16±30.3 |
| 1-Octanol | 655.72±69.82 | 602.68±41.27 | 587.69±62.66 | 507.06±45.53 | 276.81±4.55 | 331.04±24.7 | 318.93±9.83 | 401.25±12.35 | 716.57±22.52 | 437.37±16.51 | 644.81±9.21 |
| 1-Octen-3-ol | 319.47±3.46 | 272.17±25.5 | 234.99±18.14 | 228.13±6.18 | 975.36±68.12 | 254.6±11.63 | 180.87±10.26 | 152.63±0.66 | 179.19±9.04 | 240.76±15.02 | 293.14±16.15 |
| 3-Octanol | 83.73±4.88 | 101.13±8.75 | 111.77±12.49 | 73.81±2.66 | 37.02±1.14 | 62±1.99 | 64.3±3.45 | 54.2±2.1 | 60.67±2.12 | 67.21±3.92 | 147.01±12.49 |
| 1-Nonanol | 115.57±4.82 | 131.07±14.96 | 139.63±4.51 | 131.31±13.53 | 96.47±5.97 | 94.08±0.77 | 79.33±3.07 | 89.03±0.56 | 104.49±3.69 | 4.73±0.2 | 138.17±9.91 |
| 2-Nonanol | 14.3±0.96 | 22.7±2.53 | 19.77±1.66 | 11.54±0.84 | 3.34±0.34 | 5.43±0.17 | 5.93±0.25 | 3.66±0.31 | 15.3±0.61 | 14.64±1.62 | 22.45±2.4 |
| (E)-2-Nonen-1-ol | 840.09±77.31 | 635.45±4.12 | 482.21±34.56 | 665.14±14.56 | 16895.31±1961.97 | 637.51±9.06 | 126.62±2.17 | 151.01±13.77 | 343.27±5.15 | 459.86±30.31 | 444.78±52.26 |
| 1-Decanol | 0±0 | 0±0 | 1.18±0.14 | 0±0 | 2.29±0.19 | 0.41±0.03 | 1.39±0.04 | 0±0 | 0±0 | 0±0 | 0±0 |
| 2-Undecanol | 45.62±2.18 | 50.98±2.44 | 49.08±4.01 | 25.27±0.89 | 28.07±2.22 | 30.92±3.49 | 41.25±0.34 | 26.64±0.74 | 17.69±0.39 | 13.24±0.96 | 26.61±3.32 |
| 1,2-Propanediol | 30464.58±246.98 | 30544.88±163.52 | 30641.16±299.37 | 35491.83±1163.89 | 19760.62±115.96 | 25772.48±107.83 | 24936.33±234.71 | 31158.95±79.28 | 32885.87±1087.3 | 26636.5±385.49 | 25429.1±123.35 |
| (R,R)-2,3-Butanediol | 63987.95±1078.7 | 65124.51±538.75 | 60548.96±549.11 | 72798.95±707.12 | 57855.84±397.29 | 55207.52±445.84 | 47339.44±917.94 | 53374.29±348.64 | 70127.86±409.29 | 72848.45±913.39 | 71096.99±506.28 |
| Meso-2,3-Butanediol | 13888.04±114.74 | 13805.25±173.9 | 14269.34±92.64 | 13977.41±127.49 | 13315.48±393.4 | 10441.93±186.86 | 10781.39±208.96 | 8762.44±195.64 | 13137.87±241.99 | 12470.16±37.75 | 13092.52±95.32 |
| Benzyl alcohol | 591.17±37.73 | 643.74±20.96 | 1868.34±168.99 | 729.67±37.92 | 586.67±33.76 | 351.05±4.7 | 559.74±14.15 | 124.83±1.78 | 2007.31±95.54 | 1113.61±105.39 | 1037.45±113.28 |
| 2-Phenethyl alcohol | 10393.25±215.77 | 10480.59±211.75 | 10381.65±262.58 | 10768.52±292.7 | 9270.19±220.33 | 9939.41±235.92 | 9429.71±242.37 | 6924.5±123.49 | 10025.4±199.84 | 11313.2±264.03 | 11279.77±179.95 |
| Formic acid^a^ | 124.96±0.44 | 137.15±1.58 | 124.7±1.11 | 159.21±0.99 | 67.08±0.4 | 100.38±0.46 | 89.58±1.97 | 95.64±0.33 | 152.33±0.56 | 185.67±1.08 | 245.63±0.82 |
| Acetic acid^a^ | 1678.36±0.95 | 1690.55±0.71 | 1762.75±1.36 | 1661.17±0.28 | 1474.15±1.71 | 1584.82±0.62 | 1575.59±0.04 | 1428.92±1.14 | 1567.06±2.49 | 1789.55±0.56 | 1886.5±2.71 |
| Propionic acid | 72331.68±1223.71 | 80535.98±7249.61 | 68704.63±5474.4 | 66797.36±4406.83 | 40987.01±2118.97 | 47450.15±1135.33 | 47543.49±173.65 | 107815.76±3655.95 | 82765.28±5992.18 | 99950.48±6180.63 | 64223.54±6501.37 |
| Butanoic acid | 29421.25±61.46 | 28731.09±106.58 | 28010.31±111.26 | 28544.43±57.65 | 18915.89±103.59 | 25047.75±17.05 | 27790.84±126.79 | 24084.14±9.37 | 26560.55±25.94 | 29034.7±25.13 | 29774.01±127.31 |
| 2-Methylpropanoic acid | 65179.7±6.91 | 64339.14±76.91 | 56708.15±39.54 | 60243.08±95.9 | 29190.94±2.54 | 70080.95±457.45 | 65039.72±89.27 | 41581.65±126.4 | 61187.09±293.41 | 63671.55±112.01 | 60736.84±80.6 |
| Pentanoic acid | 82323.32±270.72 | 80562.01±67.96 | 80717.69±18.74 | 88521.92±181.84 | 56697.48±26.1 | 75107.45±193.96 | 84305.98±538.25 | 69261.06±34.84 | 79812.76±85.68 | 88435.71±49.4 | 92708.31±111.08 |
| 3-Methylbutanoic acid | 20807.06±32.63 | 20818.42±13.86 | 20735.46±64.85 | 21670.25±70.83 | 18327.72±6.88 | 24421.59±95.98 | 23793.92±38.54 | 19280.66±8.84 | 20833.36±40.1 | 21679.47±9.54 | 20280.73±14.19 |
| Hexanoic acid | 20541.5±59.83 | 20578.49±52.28 | 20199.94±3.73 | 17175.34±3.36 | 18189.91±25.61 | 37873.27±98.39 | 35920±15.5 | 27841.66±25.05 | 26688.5±35.76 | 23242.83±40.87 | 20496.22±152.69 |
| Octanoic acid | 19675.06±134.29 | 19708.6±132.73 | 20000.14±142.6 | 19747.51±141.46 | 20286.96±11.39 | 20636.27±32.28 | 20257±20.63 | 19587.79±27.76 | 19833.08±123.56 | 19795.82±115.8 | 19746.03±157.03 |
| Decanoic acid | 9165.19±23.33 | 9007.67±60.36 | 9543.4±99.46 | 9374.82±95.39 | 9838.5±30.26 | 9013.71±117.6 | 9108.97±34.26 | 8967.14±22.62 | 9667.96±2.77 | 9488.26±9.41 | 9615.87±11.02 |
| Dodecanoic acid | 1136.44±36.95 | 1087.19±34.81 | 1742.29±36.93 | 1835.2±44.28 | 2666.51±55.36 | 1314.8±59.12 | 2052.89±10.97 | 928.55±10.07 | 2715.96±43.03 | 2131.76±26.62 | 2509.94±55.29 |
| Benzoic acid | 29800.51±2572.22 | 24124.01±2536.97 | 33075.41±1513.05 | 47242.79±2032.01 | 1171.04±4.4 | 39912.28±1601.95 | 23325.32±745.92 | 21048.7±1431.74 | 44733.87±2003.81 | 46749.5±4354.17 | 61922.55±2328.58 |
| L-Lactic acid^a^ | 1582.21±0.7 | 1483.55±3.49 | 1590.72±2.06 | 1290.31±2.16 | 971.64±0.41 | 1225.25±0.79 | 1143.97±3.56 | 924.73±1.35 | 1070.54±1.88 | 1313.36±2.39 | 1448.95±3.29 |
| Citric acid | 25595.27±46.55 | 28133.13±67.06 | 24905.17±31.06 | 39594.53±92.21 | 4669.77±99.84 | 27483.03±68.08 | 20747.97±47.72 | 45828.87±315.86 | 61447.23±126.43 | 44497.83±41.02 | 4689.07±7.5 |
| Palmitic acid | 9792.5±578.49 | 13015±311.9 | 9654±153.5 | 13678.5±163.71 | 12241±97.98 | 10520±7.35 | 10010±10.61 | 5871±6.53 | 18370.5±660.14 | 22905.5±416.82 | 11237.5±46.13 |
| Oleic acid | 5626.5±11.84 | 8164.5±1.22 | 5834±5.72 | 7715.5±52.66 | 7884.5±38.78 | 6866±8.16 | 7071±8.16 | 3924.5±9.39 | 9099.5±6.94 | 4683±26.94 | 6741.5±19.19 |
| Linoleic acid | 2381.5±25.72 | 3907.5±37.97 | 2517.5±23.27 | 4625±23.68 | 4457±89.81 | 3192.5±9.39 | 3451±9.8 | 1803±0.82 | 5414.5±114.72 | 5249±47.36 | 5064±14.7 |
| Acetaldehyde^a^ | 393.98±0.07 | 359.24±0.4 | 479.04±1.36 | 733.38±3.2 | 199.38±0.68 | 367.55±1.15 | 330.07±0.45 | 560.77±0.36 | 772.92±1.04 | 930.57±0.07 | 955.81±4.29 |
| 2-Methylpropanal | 6853.38±173.41 | 6390.51±66.71 | 5933.29±57.03 | 5796.56±307.27 | 2565.16±53.46 | 4907.81±81.98 | 5091.79±26.07 | 4528.22±2.51 | 6527.1±64.7 | 4858.24±390.6 | 4360.41±85.81 |
| 2-Methylbutanal | 8206.05±134.34 | 7191.78±141.48 | 6925.38±118.17 | 6125.26±28.91 | 1697.68±12.46 | 5637.19±116.53 | 6368.96±32.22 | 4484.06±16.76 | 6081.13±75.68 | 1869.39±2.2 | 1622.34±6.24 |
| 3-Methylbutanal | 50602.48±536.87 | 47458.81±365.06 | 49871.33±623.67 | 50462.19±519.1 | 14048.28±192.22 | 38463.84±499.13 | 38677.27±218.39 | 38536.2±222.42 | 51313.04±362.21 | 35371.44±299.89 | 24125.21±68.72 |
| Nonanal | 35.15±0.17 | 51.99±4.8 | 42.93±1.53 | 98.81±3.43 | 217.1±19.01 | 0±0 | 51.31±1.55 | 60.97±2.11 | 25.97±1.04 | 74.67±1.24 | 29.25±2.21 |
| Benzaldehyde | 3348.94±11.35 | 3405.28±60.68 | 3935.05±29.21 | 4162.35±32.89 | 986.09±60.61 | 2768.3±186.48 | 1943.85±135.96 | 1850.62±3.99 | 5128.74±31.94 | 3824.15±77.76 | 1836.54±19.11 |
| Benzeneacetaldehyde | 10747.87±1215.18 | 9826.73±769.97 | 1951.4±149.37 | 5271.21±46.65 | 4806.87±306.41 | 18316.87±1451.95 | 19674.74±868.64 | 2586.7±244.53 | 8944.86±697.92 | 3609.29±226.38 | 25114.36±2811.53 |
| Diethoxymethane | 3377.52±149.67 | 3590.68±50.66 | 8516.71±87.54 | 18164.37±273.4 | 987.61±51.85 | 4865.42±10.65 | 2806.85±58.31 | 12109.24±252.24 | 20532.2±334.76 | 27434.8±177.43 | 26560.59±241.58 |
| 1,1-Diethoxyethane | 308040.63±1698.76 | 261449.07±68.99 | 376366.36±150.32 | 579603.52±562.52 | 157242.16±138.44 | 277628.97±360.88 | 251018.52±150.08 | 428405.27±792.57 | 598376.58±2430.12 | 691313.45±3276.66 | 685560.57±1965.93 |
| 1,1-Diethoxy-3-methylbutane | 33790.19±1735.43 | 28886.16±1488.74 | 33054.84±964.64 | 33118.55±1207.61 | 8131.89±475.64 | 23743.48±1814.74 | 24727.35±950.45 | 24319.76±772.37 | 33489.43±337.24 | 21018.61±726.6 | 13566.4±906.75 |
| (2,2-Diethoxyethyl)-benzene | 119.13±10.2 | 117.05±7.09 | 72.97±1.94 | 64.86±2.97 | 163.95±2.99 | 107.11±2.68 | 164.12±5.6 | 33.26±0.27 | 27±0.85 | 13.98±0.02 | 109.91±3.76 |
| 1,1,3,3-Tetraethoxy-propane | 25.5±1.63 | 31±0.71 | 43.2±0.75 | 162.85±10.12 | 7.24±0.08 | 38.27±2.3 | 27.33±1.26 | 84.05±2.72 | 110.92±5.29 | 30.45±1.9 | 76.98±1.68 |
| Acetone | 30232.6±295.94 | 26727.23±19.87 | 32134.7±253.61 | 34819.92±14.74 | 8958.49±61.93 | 20537.88±25.82 | 19658.29±79.97 | 23888.32±22.64 | 37408.28±218.43 | 31371.59±774.54 | 26730.95±208.06 |
| 2-Pentanone | 7842.55±485.94 | 5349.27±303.43 | 9087.94±233.85 | 6284.16±181.89 | 3258.74±250.43 | 4797.88±39.5 | 5138.88±487.45 | 6283.81±338.52 | 15118.62±247.77 | 6100.92±360.89 | 6493.74±295.83 |
| 3-Hydroxyl-2-butanone | 54433.83±1297.87 | 55653.54±1343.57 | 52934.34±1344.93 | 69578.76±1803.5 | 10947.54±171.22 | 39733.6±855.05 | 31571.8±863.13 | 69263.01±1399.32 | 79428.44±1772.45 | 37083.91±795.65 | 5060.52±46.11 |
| 2-Heptanone | 1374.32±18.04 | 19.34±0.78 | 12.22±0.56 | 21.56±2.16 | 318.46±17.05 | 561.98±15.21 | 614.03±20.26 | 2.98±0.31 | 1579.46±123.34 | 1229.51±149.41 | 1787.28±21.29 |
| 2-Octanone | 0.1±0 | 0.28±0.02 | 1.05±0.08 | 0.57±0.07 | 3.02±0.32 | 1.09±0.07 | 0.49±0.01 | 0.75±0.05 | 0.52±0.07 | 0.99±0.06 | 0.84±0.04 |
| 3-Octanone | 42.33±1.62 | 55.43±5.43 | 44.07±4.52 | 67.46±1.27 | 127.53±6.83 | 33.12±0.9 | 33.36±2.25 | 28.24±3.05 | 37.11±4.31 | 30.6±2.91 | 85.59±7.76 |
| 2-Nonanone | 117.11±10.5 | 123.44±1.85 | 147.53±8.62 | 154.37±12.7 | 68.18±3.46 | 92.72±2.2 | 84.77±5.88 | 73.68±2.08 | 158.46±16.43 | 51.44±2.79 | 133.06±14.22 |
| 4-Nonanone | 82.31±5.94 | 32.06±3.69 | 74.93±7.53 | 46.28±0.77 | 0±0 | 78.89±0.09 | 38.16±3.87 | 1.43±0.03 | 32.8±3.1 | 34.76±1.6 | 48.22±1.52 |
| 2-Undecanone | 47.41±5.28 | 47.54±0.84 | 61.55±5.33 | 57.87±5.94 | 46.81±4.45 | 25.4±1.76 | 54.79±3.55 | 19.58±0.71 | 41.7±0.68 | 20.61±0.75 | 44.4±3.59 |
| 4-Undecanone | 1041.56±31.69 | 939.9±27.99 | 975.83±76.47 | 378.16±25.39 | 837.87±20.93 | 305.95±12.22 | 1091.58±115.46 | 0±0 | 0±0 | 0±0 | 0±0 |
| 2-Tridecanone | 17.57±1.04 | 20.85±1.86 | 40.02±2.83 | 37.47±3.43 | 45.57±3.54 | 22.85±2.57 | 37.16±1.33 | 9.1±0.47 | 18.45±0.8 | 9.73±0.39 | 28±3.03 |
| 2-Pentadecanone | 52.46±4.76 | 64.98±4.96 | 81.05±7.64 | 72.97±1.8 | 85.57±5.96 | 62.8±0.56 | 71.82±3.83 | 30.88±1.33 | 43.73±4.57 | 29.19±1.98 | 63.25±1.61 |
| Furfuryl alcohol | 19016.02±322.12 | 16689.43±366.34 | 14175.61±201.89 | 7952.94±138.01 | 14212.86±1021.44 | 11354.2±67.94 | 14373.49±1344.69 | 5199.25±30.09 | 6078.51±114.54 | 4244.19±59.46 | 3764.04±86.37 |
| Furfural^a^ | 266.75±2.87 | 259.79±7.43 | 254.86±5.58 | 282.83±6.13 | 27.29±0.45 | 195.97±7.51 | 161.05±2.67 | 236.51±2.58 | 318.54±5.92 | 210.56±3.38 | 183.99±4.51 |
| 5-Methylfurfural | 1093.7±18.81 | 675.99±39.08 | 739.91±23.89 | 584.74±30.18 | 325.77±9.54 | 1255.04±42.51 | 358.74±27.42 | 540.48±38.27 | 985.4±70.41 | 517.73±5.02 | 691.93±41.42 |
| Furfuryl ethyl ether | 11107.34±171.6 | 10738.68±162.84 | 10770.75±99.89 | 7271.18±93.8 | 3063.64±38.54 | 6455.08±138.16 | 6202.35±22.36 | 4033.77±15.74 | 4144.58±66.1 | 3746.32±33.01 | 3128.43±13.67 |
| 2-Acetylfuran | 2054.3±32.9 | 1350.98±109.39 | 1101.4±28.62 | 1342.83±6.78 | 1521.66±87.02 | 2109.52±17.31 | 938.98±41.21 | 1001.56±32.58 | 1041.26±25.93 | 1097.07±24.4 | 1249.74±82.02 |
| HDMF | 60.91±2.71 | 56.81±2.54 | 66.06±1.35 | 79.79±2.46 | 23±0.38 | 53.5±0.96 | 43.7±1.67 | 77.49±0.77 | 83.56±2.73 | 101.55±2.84 | 76.75±3.23 |
| HEMF | 12.83±0.57 | 2.49±0.09 | 1.77±0.18 | 3.62±0.07 | 0±0 | 4.12±0.13 | 0±0 | 9.54±0.39 | 1.61±0.2 | 10.19±0.61 | 13.16±0.76 |
| Sotolon | 27.34±1.66 | 36.07±0.51 | 39.08±1.11 | 57.04±1.16 | 9.36±0.18 | 27.68±1.19 | 17.25±0.25 | 33.96±1.02 | 48.39±1.05 | 86.14±1.67 | 148.75±2.84 |
| Pyrazine | 60.06±0.74 | 29.8±0.82 | 31.08±0.59 | 54.99±1.02 | 21.04±2.55 | 34.93±0.12 | 45.8±0.76 | 18.28±0.19 | 30.77±0.26 | 36.33±0.36 | 59.91±0.2 |
| 2-Methylpyrazine | 731.42±6.4 | 99.53±2.52 | 625.1±0.81 | 809.93±8.17 | 181.71±2.69 | 245.22±4.33 | 277.24±5.5 | 162.13±4.18 | 380.69±3.47 | 558.01±5.62 | 505.61±5.98 |
| 2,3-Dimethylpyrazine | 644.22±2.54 | 267.61±1.87 | 560.97±4.45 | 586.33±2.98 | 30.91±1.46 | 426.68±3.14 | 727.97±0.84 | 190.52±0.22 | 239.95±0.72 | 277.18±1.12 | 256.79±1.06 |
| 2,5-Dimethylpyrazine | 4723.85±101.55 | 1784.28±6.18 | 4109.76±66.67 | 5650.24±105 | 859.15±8.64 | 2168.06±97.99 | 3584.48±66.22 | 1573.24±34.42 | 2306.1±99.64 | 3860.01±162.89 | 3377.05±67.03 |
| 2,6-Dimethylpyrazine | 963.47±3.88 | 301±9.72 | 843.22±3.68 | 1306.88±5.91 | 159.44±3.04 | 422.98±7.46 | 992.55±14.9 | 341.82±10.87 | 331.07±1.25 | 816.25±4.22 | 858.68±5.12 |
| 2-Ethylpyrazine | 247.31±3.25 | 3.05±0.61 | 135.99±2.51 | 260±5.45 | 0±0 | 83.71±2.48 | 181.12±0.47 | 7.42±1.51 | 110.11±2.78 | 185.21±2.8 | 197.73±2.65 |
| 2-Ethyl-3-methylpyrazine | 127.02±1.49 | 16.19±0.52 | 96.67±1.71 | 128.04±1.33 | 29.37±2.22 | 56.11±1.67 | 137.22±2.26 | 0±0 | 46.37±0.21 | 68.31±1.16 | 73.44±1.29 |
| 2-Ethyl-6-methylpyrazine | 3700.47±120.17 | 907.5±1.92 | 2557.02±23.97 | 5511.39±100.35 | 642.49±4.8 | 1555.53±21.18 | 2197.45±69.61 | 709.17±3.88 | 2249.08±52.81 | 3510.48±65.36 | 3929.31±39.69 |
| 2,3,5-Trimethylpyrazine | 9604.45±105 | 3732.29±33.09 | 7694.62±67.01 | 8612.65±120.17 | 976.37±8.41 | 4693.73±138.76 | 7790.95±66.18 | 2881.84±25.47 | 4207.07±36.07 | 5921.97±31.92 | 5152.79±25.11 |
| 2-Ethyl-3,5-dimethylpyrazine | 6475.45±290.67 | 2658.1±17.45 | 5792.79±101.11 | 12005.81±176.22 | 783.26±6.07 | 4910.99±107.84 | 4683.96±118.55 | 1971.43±4.19 | 5833.44±140.63 | 5955.72±158.34 | 7071.87±167.65 |
| 2-Ethyl-3,6-dimethylpyrazine | 430.57±3.25 | 150.3±0.92 | 387.46±0.42 | 345.15±0.94 | 125.34±1.5 | 229.83±1.41 | 282.56±1.11 | 119.68±1.75 | 162.24±1.96 | 276.6±2.96 | 291.15±3.26 |
| 2,3,5,6-Tetramethylpyrazine | 24999.12±254.83 | 10575.05±19.67 | 20628.51±331.31 | 10273.71±213.1 | 3689.65±35.39 | 11083.36±178.63 | 18975.21±109.33 | 3246.07±6.49 | 3485.88±7.47 | 6442.77±173.56 | 6811.51±67.94 |
| 2,3-Diethylpyrazine | 59.48±0.85 | 0±0 | 60.42±2.21 | 69.43±1.39 | 57.22±1.03 | 43.71±2.95 | 60.04±2.85 | 0±0 | 9.36±1.45 | 27.95±1.68 | 35.26±2.8 |
| 2,3-Diethyl-5-methylpyrazine | 126.79±3.78 | 40.93±1.06 | 62.42±0.09 | 63.65±0.18 | 90.21±0.61 | 58.32±0.49 | 50.72±1.19 | 32.87±0.44 | 31.29±0.76 | 67.17±0.16 | 64.06±0.85 |
| 2-Isobutyl-3-methylpyrazine | 187±2.5 | 70.28±0.64 | 174.76±1.13 | 133.67±0.87 | 159.14±1.29 | 124.47±1.72 | 149.33±1.21 | 67.95±1.1 | 68.46±1.88 | 138.73±1.23 | 115.24±1.86 |
| (Trimethylpyrazin-2-yl)methanol | 83.54±0.15 | 24.74±0.08 | 72.9±0.06 | 25.14±0.1 | 0.92±0.02 | 15.24±0.13 | 47.65±0.21 | 0±0 | 1.67±0.06 | 16.12±0.09 | 31.02±0.13 |
| Dimethyl trisulfide | 246.42±29 | 336.15±21.39 | 188.9±4.86 | 42.61±4.32 | 671.68±36.01 | 165.94±1.38 | 163.67±13.65 | 59.29±4.84 | 71.38±2.01 | 29.09±2.91 | 36.04±0.35 |
| Phenol | 1430.32±2.16 | 1169.61±9.35 | 763.5±16.65 | 1080.42±24.74 | 2692.28±27.5 | 1022.31±1.76 | 420.13±2.14 | 1353.97±97.19 | 2294.43±11.47 | 753.25±16.98 | 860.2±39.62 |
| p-Cresol | 429.3±12.11 | 360.9±13.27 | 370.18±32.28 | 577.34±26.98 | 1067.82±11.96 | 380.04±8.19 | 222.43±5.22 | 425.31±45.87 | 770.77±75.68 | 650.38±28.44 | 1238.36±32.79 |
| Guaiacol | 579.87±6.07 | 421.27±1.94 | 362.61±27.51 | 300.68±14.21 | 278.54±5.52 | 435.77±4.03 | 315.08±5.62 | 333.35±24.66 | 531.04±9.88 | 257.89±21 | 242.25±26.34 |
| 4-Methylguaiacol | 30.68±1.76 | 29.49±0.51 | 39.62±4.68 | 25.05±0.45 | 388.17±9.39 | 20.68±1.06 | 63.39±2.43 | 18.71±0.45 | 17.38±0.38 | 17.18±0.65 | 25.81±2.58 |
| 4-Ethylguaiacol | 572.06±20.36 | 478.9±12.32 | 506.19±41.99 | 379.21±5.66 | 2236.84±134.96 | 232.32±17.53 | 439.69±7.04 | 201.59±10.65 | 728.72±28.26 | 284.2±3.37 | 486.44±39.33 |
| 4-Vinylguaiacol | 3.07±0.24 | 2.46±0.25 | 4.87±0.48 | 7.95±0.45 | 619.45±5.12 | 4.8±0 | 1.09±0.03 | 6.02±0.31 | 1.28±0.02 | 0.81±0.05 | 1.08±0.04 |
| gama-Nonalactone | 295.42±2.31 | 242.68±6.66 | 195.91±16.85 | 245.26±20.83 | 149.28±8.19 | 287.45±16.24 | 197.36±0.4 | 77.51±6.23 | 175.99±9.69 | 222.71±5.23 | 255.14±7.54 |
| gama-Dodecalactone | 511.62±1.89 | 432.15±16.67 | 482.74±45.89 | 620.43±23.63 | 1084.34±64 | 484.8±6.44 | 336.87±1.74 | 323.73±14.42 | 588.66±30 | 615.02±30.53 | 676.3±2.69 |
| Phytone | 56.98±5.34 | 69.84±4.89 | 69.25±7.17 | 135.29±7.06 | 59.39±5.41 | 121.66±9.78 | 75.26±5.52 | 54.39±3.46 | 55.16±6.05 | 42.73±3.12 | 92.51±5.64 |
| beta-Damascone | 11.59±0.02 | 10±0.87 | 4.54±0.29 | 3±0.3 | 35.2±0.71 | 7.29±0.69 | 3.14±0.33 | 2.55±0.26 | 3.49±0.23 | 1.95±0.05 | 1.11±0.05 |
| Geranylacetone | 35.85±2.68 | 45.01±1.32 | 48.85±1.04 | 44.8±2.21 | 46.08±3.4 | 48.89±3.58 | 54.51±0.87 | 29.25±1.09 | 21.75±0.42 | 4.93±0.32 | 18.31±1.19 |

Data represent mean ± SD (n = 3).

^a^The unit of compound is mg/L.

Table S4 The average content of 154 flavor compounds in XJ town samples (μg/L)

| Name | XJ-1 | XJ-2 | XJ-3 | XJ-4 | XJ-5 |
| --- | --- | --- | --- | --- | --- |
| Ethyl formate | 51169.68±3349.06 | 68004.93±1924.88 | 91185.48±3068.84 | 75379.7±812.92 | 76228.71±489.88 |
| Ethyl acetate^a^ | 2892.9±95.38 | 3121.8±69.89 | 3599.04±54.22 | 2975.7±70.55 | 3093.91±96.54 |
| Propyl acetate | 11513.87±752.96 | 16551.25±99.45 | 29079.06±345.09 | 12697.92±685.34 | 12237.95±617.86 |
| 3-Methylbutyl acetate | 8175.78±93.07 | 9170.74±46.99 | 9742.8±158.89 | 8540.25±634.13 | 9048.43±773.12 |
| Hexyl acetate | 293.24±31.85 | 48.23±5.63 | 289.57±19.84 | 53.72±3.37 | 295.15±9.81 |
| 2-Phenylethyl acetate | 53.13±3.19 | 37.48±1.97 | 16.61±1.31 | 116.79±0.95 | 122.64±12.8 |
| Ethyl propanoate | 33347.43±1490.46 | 30924.49±295.39 | 50387.74±3065.92 | 28331.39±1736.07 | 26553.66±1821.21 |
| Ethyl 2-methylpropanoate | 14305.05±779.47 | 13510.53±736.28 | 13534.71±417.97 | 12932.52±801.71 | 13821.99±871.78 |
| Ethyl butanoate | 31941.19±966.68 | 23117.46±505.9 | 21781.37±738.56 | 25312.43±948.83 | 28615.35±1027.18 |
| Butyl butanoate | 52.56±2.06 | 45.06±0.52 | 40.4±2.91 | 74.16±2.27 | 64.57±6.35 |
| 3-Methylbutyl butanoate | 214.69±1.24 | 8.96±0.06 | 444.33±52.46 | 0±0 | 159.45±16.6 |
| 2-Phenethyl buanoate | 273.53±3.27 | 261.53±27.84 | 252.09±10.55 | 181.67±16.64 | 339.31±33.53 |
| Ethyl 2-methylbutanoate | 2780.94±205.56 | 2759.42±68.23 | 3702.28±201.77 | 2884.51±286.71 | 3266.44±245.6 |
| Ethyl 3-methylbutanoate | 10357.26±286.44 | 10692.17±321.7 | 12645.17±437.96 | 11159.98±471.02 | 12139.89±358.19 |
| Ethyl pentanoate | 2870.17±26.4 | 1341.45±109.31 | 2664.6±112.51 | 4379.57±91.41 | 4507.03±145.55 |
| 3-Methylbutyl pentanoate | 115.41±6.94 | 108.79±5.31 | 181.64±16.6 | 131.18±9.21 | 230.88±17.34 |
| Ethyl 4-methylpentanoate | 204.68±19.18 | 117.96±14.07 | 393.6±42.78 | 162.36±3.87 | 254.49±9.84 |
| Ethyl hexanoate | 37349.55±91.02 | 30224.22±59.69 | 32509.02±134.24 | 35299.27±86.58 | 36749±210.2 |
| Propyl hexanoate | 675.07±25.65 | 298.62±8.52 | 873.12±54.96 | 583.96±13.75 | 651.38±44.43 |
| Butyl hexanoate | 19.07±0.16 | 27.51±2.46 | 23.75±2.91 | 17.52±0.41 | 48.52±4.69 |
| 2-Methylpropyl hexanoate | 10.32±0.07 | 9.07±0.04 | 9.28±0.02 | 20.95±1.21 | 10.9±1.34 |
| 3-Methylbutyl hexanoate | 5291.21±75.25 | 3472.76±248.34 | 2695.54±314.37 | 2035.14±71.13 | 1624.77±18.89 |
| Hexyl hexanoate | 653.98±18.14 | 439.78±30.39 | 345.48±5.31 | 725.82±43.39 | 484.45±14.6 |
| 2-Phenethyl hexanoate | 2473.51±65.62 | 2484.08±137.38 | 2584.13±17.44 | 2106.54±130.72 | 4239.09±213.39 |
| Ethyl 5-methylhexanoate | 24.13±1.41 | 4.45±0.12 | 8.79±1.06 | 5.87±0.04 | 17.8±1.29 |
| Ethyl heptanoate | 631.49±24.89 | 308.47±10.14 | 1466.36±150.33 | 708.31±12.54 | 792.47±52.19 |
| Ethyl octanoate | 1926.94±213.5 | 1701.22±101.53 | 1932.1±21.19 | 2201.93±22.85 | 2003.53±59.57 |
| Ethyl nonanoate | 674.02±67.19 | 537.76±57.61 | 615.61±67.54 | 787.51±2.57 | 781.86±23.45 |
| Ethyl decanoate | 271.87±14.39 | 595.33±52.65 | 1266.12±86.87 | 26.95±2.56 | 708.38±18.18 |
| (E)-Ethyl 4-decenoate | 100.16±4.08 | 214.83±12.4 | 136.04±8.83 | 90.83±4.61 | 154.48±5.61 |
| Ethyl undecanoate | 5.5±0.1 | 6.87±0.72 | 6.16±0.43 | 6.73±0.09 | 8.71±0.6 |
| Ethyl dodecanoate | 295.75±6.52 | 336.27±20.58 | 232.63±6.46 | 296.47±14.81 | 360.86±25.53 |
| Ethyl tridecanoate | 42.43±1.23 | 43.43±4.25 | 32.47±0.84 | 30.42±1.89 | 39.03±2.19 |
| Ethyl tetradecanoate | 1105.78±33.53 | 1171.44±105.12 | 1030.4±66.18 | 959.59±83.59 | 1271.61±71.57 |
| Ethyl pentadecanoate | 175.5±10.89 | 187.47±5.21 | 226.98±27.1 | 126.24±9.66 | 164.73±6.73 |
| Ethyl hexadecanoate | 22501.06±242.96 | 26576.41±360.35 | 23248.87±460.5 | 19037.87±233.97 | 21609.18±303.72 |
| Ethyl 9-hexadecenoate | 1835.26±139.79 | 2636.06±280.37 | 2026.53±45.68 | 1529.95±88.9 | 1822.42±17.98 |
| Ethyl heptadecanoate | 28.66±0.08 | 45.25±2.41 | 65.87±6.45 | 39.75±0.67 | 48.64±4.2 |
| Ethyl octadecanoate | 24.75±0.75 | 73.21±1.22 | 77.2±3.06 | 67.22±0.79 | 72.54±0.36 |
| Ethyl oleate | 7485.51±135.04 | 9816.89±58.3 | 8223.2±164.84 | 5404.75±169.67 | 6659.17±151.83 |
| Ethyl linoleate | 12962.65±181.93 | 17229.52±329.33 | 13491.38±298.74 | 8384.42±249.52 | 10419.42±431.42 |
| Ethyl linolenate | 508.43±8.76 | 405.93±22.61 | 344.16±23.05 | 152.36±7.47 | 228.62±2.86 |
| Diethyl butanedioate | 2735.82±67.23 | 3014±103.75 | 1517.68±88.03 | 3075.57±46.46 | 2638.13±304.85 |
| Diethyl azeleate | 490.1±36.95 | 492.61±5.69 | 1052.14±25.24 | 539.81±40.77 | 657.18±18.75 |
| Ethyl lactate^a^ | 2216.37±85.39 | 2197.22±69.37 | 2089.23±120.83 | 2187.69±101.8 | 2336.69±82.96 |
| 3-Methylbutyl lactate | 2933.65±167.74 | 2069.48±33.33 | 474.82±42.65 | 3118.7±31.89 | 3068.41±194.89 |
| Ethyl 2-furancarboxylate | 107.62±6.79 | 36.53±2.93 | 1379.86±86.14 | 36.81±0.27 | 904.56±12.93 |
| Ethyl 2-hydroxy-4-methylpentanoate | 11959.64±353.11 | 11986.54±285.96 | 12033.75±528.6 | 12149.25±476.68 | 13269.31±681.1 |
| Ethyl 2-hydroxy-hexanoate | 26139.03±1266.49 | 33181.51±1237.43 | 13147.25±246.89 | 31314.42±1075.82 | 22149.1±1633.38 |
| Ethyl benzoate | 7537.75±512.94 | 9715.45±276.56 | 8602.5±812.67 | 3565.81±125.06 | 7607.77±296.98 |
| Ethyl phenylacetate | 3522.96±237.49 | 3295.85±96.43 | 3225.84±235.08 | 3374.64±296.33 | 3827.89±309.46 |
| Ethyl 3-phenylpropionate | 36.82±1.97 | 31.84±3.5 | 132.96±11.01 | 26.33±2.25 | 64.26±1.09 |
| Methanol^a^ | 189.47±1.9 | 202.88±1.54 | 208.66±3.32 | 189.31±1.18 | 201.15±0.87 |
| 1-Propanol^a^ | 2003.58±4.66 | 2657.54±4.73 | 2081.75±21.66 | 2310.26±15.26 | 2328.14±19.5 |
| 1-Butanol | 78790.37±117 | 74057.68±241.04 | 68115.06±713.2 | 80875.6±809.61 | 96960.82±506.01 |
| 2-Butanol | 72355.64±458.54 | 61770.26±516.48 | 84032.72±1539.99 | 49714.16±41.71 | 53940.44±786.03 |
| 2-Methylpropanol^a^ | 155.74±0.43 | 165.82±0.68 | 190.49±3.06 | 170.79±0.6 | 180.45±1.69 |
| 1-Pentanol | 9456.65±202.88 | 8861.05±220.02 | 9018.2±380.38 | 9189.81±386.48 | 9834.35±397.52 |
| 2-Pentanol | 2092.44±46.02 | 1560.36±49.95 | 1583.25±67.6 | 1221.74±53.07 | 1378.82±31.1 |
| 2-Methylbutanol | 43448.46±1727.34 | 45550.07±2283.76 | 60798.02±2654.91 | 58046.31±2894.31 | 60670.32±3429.35 |
| 3-Methylbutanol^a^ | 275.7±3.4 | 291.08±3.39 | 311.96±10.91 | 297.75±11.37 | 319.6±10.58 |
| 1-Hexanol | 8605.54±302.29 | 5012.94±7.79 | 4851.88±311.22 | 5926.53±284.77 | 8012.88±276.88 |
| 1-Heptanol | 117.12±4.48 | 620.65±38.85 | 245.39±17.85 | 245.39±17.85 | 285.78±26.17 |
| 2-Heptanol | 2082.19±109.47 | 2691.91±185.35 | 3759.43±270.86 | 3759.43±270.86 | 585.33±15.16 |
| 1-Octanol | 642.68±27.96 | 563.5±12.66 | 372.6±8.71 | 372.6±8.71 | 405.62±20.27 |
| 1-Octen-3-ol | 152.36±8.76 | 203.66±2.2 | 2.89±0.3 | 2.89±0.3 | 269.13±8.18 |
| 3-Octanol | 109.18±11.87 | 78.48±1.78 | 58.94±6.62 | 58.94±6.62 | 87.01±2.82 |
| 1-Nonanol | 156.04±2.42 | 38.16±0.81 | 116.09±11.24 | 116.09±11.24 | 131.69±12.67 |
| 2-Nonanol | 10.13±1.03 | 8.87±0.81 | 17.51±2.02 | 17.51±2.02 | 21.23±2.09 |
| (E)-2-Nonen-1-ol | 641.26±7.98 | 412.82±8.57 | 790.16±59.48 | 723.5±25.82 | 372.03±19.78 |
| 1-Decanol | 1.1±0.11 | 0.84±0.04 | 0±0 | 0±0 | 0.96±0.1 |
| 2-Undecanol | 80.26±3.46 | 44.44±3.89 | 33.29±2.63 | 33.29±2.63 | 75.97±3.63 |
| 1,2-Propanediol | 23924.41±1967.4 | 23267.8±2276.14 | 23951.18±2741.98 | 24045.96±1966.81 | 24210.62±2374.56 |
| (R,R)-2,3-Butanediol | 49677.46±3685.89 | 49450.05±3615.83 | 57850.03±4220.85 | 53454.68±4457.99 | 57592.41±5005.65 |
| Meso-2,3-Butanediol | 13155.32±332.3 | 13702.57±376.15 | 16166.33±403.37 | 14407.48±416.43 | 15958.7±670.12 |
| Benzyl alcohol | 1607.18±45.76 | 308.72±27.51 | 333.28±4.47 | 346.44±37.9 | 374.66±10.61 |
| 2-Phenethyl alcohol | 11177.01±480.87 | 10771.77±573.24 | 11165.21±849.01 | 11964.92±633.54 | 12759.38±666.34 |
| Formic acid^a^ | 146.32±0.99 | 164.53±1.04 | 162.47±0.15 | 163.25±1.43 | 151.73±1.37 |
| Acetic acid^a^ | 1956.89±2.86 | 2022.43±4.13 | 2262.6±2.9 | 1943.4±1.37 | 1884.18±1.53 |
| Propionic acid | 53066.53±375.52 | 49894.47±431.37 | 60449.46±405.32 | 47261.37±296.27 | 46816.65±384.54 |
| Butanoic acid | 29775.64±88.9 | 28773.56±69.45 | 26867.52±35.43 | 27342.53±58.68 | 27062.88±74.64 |
| 2-Methylpropanoic acid | 31409.47±26.06 | 26090±79.31 | 25394.12±167.93 | 27686.39±158.81 | 28351.09±244.42 |
| Pentanoic acid | 97768.81±862.9 | 97924.5±233.63 | 94429.02±357.76 | 93673.75±351.82 | 93371.53±681.21 |
| 3-Methylbutanoic acid | 18223.25±101.17 | 17349.08±58.53 | 17874.03±41.18 | 19045.32±31.26 | 18970.66±53.83 |
| Hexanoic acid | 21231.19±9.98 | 16130.95±39.5 | 17406.67±31.56 | 19365.12±103.96 | 19710.54±5.35 |
| Octanoic acid | 20046.98±4.02 | 19716.49±89.13 | 19773.4±17.29 | 19901.85±49.22 | 19879.75±66.81 |
| Decanoic acid | 9234.77±128.29 | 9239.47±84.28 | 9154.08±91.98 | 9087.89±78.04 | 9055.11±43.12 |
| Dodecanoic acid | 1242.06±31.06 | 1744.49±25.81 | 1292.03±37.25 | 630.26±14.22 | 889.47±45.56 |
| Benzoic acid | 24970.34±758.88 | 22645.22±873.87 | 55459.91±5460.41 | 20560.95±1138.69 | 58289.71±4946.06 |
| L-Lactic acid^a^ | 1750.81±1.65 | 1761.04±3.31 | 1568.25±1.74 | 1731.94±2.9 | 1736.75±4.18 |
| Citric acid | 31919.83±24.3 | 41468.2±44.35 | 56531.93±624.36 | 62965.6±13.25 | 37387.67±268.88 |
| Palmitic acid | 5534±39.19 | 10257±57.15 | 5919±115.13 | 3133±8.16 | 4325±50.62 |
| Oleic acid | 6564±43.27 | 8350.5±23.27 | 5730±22.05 | 3836±16.33 | 4453±22.86 |
| Linoleic acid | 2456.5±53.48 | 3600.5±24.09 | 2487.5±33.88 | 1402.5±22.45 | 1825±0.82 |
| Acetaldehyde^a^ | 368.89±4.52 | 456.64±8.46 | 626.09±36.82 | 510.12±18.85 | 513.27±14.8 |
| 2-Methylpropanal | 9353.23±187.14 | 10995.65±214.83 | 9631.92±439.87 | 8710.27±448.04 | 9337.44±419.85 |
| 2-Methylbutanal | 10942.8±371.19 | 11701.19±299.32 | 9328.16±413.48 | 7904.39±364.11 | 9186.18±288.74 |
| 3-Methylbutanal | 53609.21±955.28 | 55547.68±540.6 | 44520.24±1221.23 | 37127.3±1029.22 | 42898.09±954.15 |
| Nonanal | 31±0.43 | 56.16±4.98 | 24.6±0.29 | 39.46±2.83 | 22.76±0.87 |
| Benzaldehyde | 2388.75±95.54 | 1997.89±206.76 | 1517.44±68.78 | 1516.69±58.9 | 1630.06±95.33 |
| Benzeneacetaldehyde | 49452.26±1678.77 | 11829.93±893.23 | 12573.16±1123.28 | 29443.11±964.92 | 14342.99±1171.02 |
| Diethoxymethane | 5664.59±216.38 | 6472.19±153.04 | 7794.78±165.8 | 6858.64±132.05 | 6459.06±278.31 |
| 1,1-Diethoxyethane | 282683.48±10545.57 | 349367.19±8512.85 | 440085.96±5800.5 | 365008.16±13323.33 | 365045.32±15676.53 |
| 1,1-Diethoxy-3-methylbutane | 36564.04±2967.47 | 37336.76±2448.59 | 28105.08±2782.23 | 23617.64±2923.67 | 27554.6±2757.5 |
| (2,2-Diethoxyethyl)-benzene | 38.36±3.19 | 66.22±1.99 | 76.71±7.09 | 30.84±0.3 | 253.18±3.24 |
| 1,1,3,3-Tetraethoxy-propane | 34.97±2.15 | 24.07±0.71 | 140.52±11.39 | 18.56±0.63 | 66.79±0.82 |
| Acetone | 24188.81±2618.79 | 25656.74±2209.84 | 25012.44±1181.39 | 19984.33±1306.21 | 21851.17±1800.91 |
| 2-Pentanone | 14174.41±843.42 | 14211.52±731.1 | 10726.55±1100.03 | 10982.47±202.72 | 10570.79±1170.36 |
| 3-Hydroxyl-2-butanone | 21412.64±991.35 | 23627.55±988.89 | 28038.95±1960.63 | 17274.26±968.01 | 21224.66±1063.14 |
| 2-Heptanone | 1098.38±45.37 | 177.78±5.28 | 15.59±1.01 | 629.24±38.75 | 579.99±65.44 |
| 2-Octanone | 0.35±0.02 | 0.58±0.04 | 0.54±0.04 | 0.4±0.04 | 0.31±0.03 |
| 3-Octanone | 36.79±2.9 | 11.07±0.16 | 115.15±8.47 | 32.48±3.95 | 59.01±5.96 |
| 2-Nonanone | 155.09±6.45 | 0±0 | 123.1±13.08 | 0±0 | 133.41±10.37 |
| 4-Nonanone | 42.56±0.75 | 59.42±3.44 | 79.52±3.19 | 30.56±2.42 | 92.73±4.7 |
| 2-Undecanone | 77.48±4.31 | 77.3±7.06 | 40.12±1 | 64.33±1.06 | 79.9±2.63 |
| 4-Undecanone | 2094.84±66.8 | 1709.9±86.56 | 1936.95±49.59 | 2426.35±224.62 | 1993.8±165.79 |
| 2-Tridecanone | 35.47±1.98 | 34.29±2.64 | 13.58±0.8 | 27.43±1.25 | 41.39±4.19 |
| 2-Pentadecanone | 76.72±2.97 | 83.32±2.64 | 47.96±2.75 | 52.21±5.32 | 72.04±5.41 |
| Furfuryl alcohol | 22527.14±1580.82 | 20321.44±1798.23 | 15814.48±618.93 | 16864.3±722.76 | 18534.34±551.34 |
| Furfural^a^ | 290.71±21.44 | 308.3±19.91 | 304.8±14.99 | 239.84±25.09 | 258.91±19.06 |
| 5-Methylfurfural | 509.59±15.13 | 608.16±73.27 | 1035.84±105.16 | 355.37±31.78 | 769.51±24.53 |
| Furfuryl ethyl ether | 13742±99.89 | 13243.78±111.79 | 10513.34±259.04 | 11831.11±298.08 | 13331.28±273.41 |
| 2-Acetylfuran | 686.11±7.17 | 1229.53±11.6 | 2160.26±59.73 | 594.24±23.49 | 1592.08±143.11 |
| HDMF | 72.41±1.66 | 58.73±1.06 | 52.94±1.06 | 57.94±0.9 | 58.47±1.26 |
| HEMF | 0±0 | 1.31±0.09 | 1.63±0.12 | 0.32±0.27 | 0±0 |
| Sotolon | 17.97±0.6 | 13.94±0.29 | 25.79±0.24 | 18.24±0.35 | 25.57±0.4 |
| Pyrazine | 54.34±0.52 | 45.13±0.53 | 38.22±0.3 | 26.41±0.28 | 26.05±0.16 |
| 2-Methylpyrazine | 300.1±1.28 | 300.78±0.13 | 231.55±0.74 | 138.65±0.84 | 190.89±2.68 |
| 2,3-Dimethylpyrazine | 130.81±0.74 | 132.18±0.09 | 628.71±0.92 | 414.24±0.9 | 410.82±1.04 |
| 2,5-Dimethylpyrazine | 2210.3±16.82 | 2503.15±27.51 | 2167.08±1.09 | 1540.17±2.92 | 1298.55±2.69 |
| 2,6-Dimethylpyrazine | 442.59±1.02 | 537.57±4.94 | 306.8±2.42 | 157.67±1.97 | 244.89±1.46 |
| 2-Ethylpyrazine | 85.65±0.6 | 98.62±1.31 | 86.61±1.28 | 0±0 | 0±0 |
| 2-Ethyl-3-methylpyrazine | 39.75±0.24 | 38.26±0.66 | 38.64±0.09 | 10.86±0.32 | 26.64±0.81 |
| 2-Ethyl-6-methylpyrazine | 1368.55±55.22 | 1711.42±35.26 | 2734.37±27.51 | 1294.64±19.4 | 1380.34±61.41 |
| 2,3,5-Trimethylpyrazine | 2555.04±16.27 | 2929.31±21.7 | 2524.06±44.11 | 1688.93±5.91 | 1822.31±10.46 |
| 2-Ethyl-3,5-dimethylpyrazine | 1889.6±13.44 | 1733.39±1.18 | 1824.46±1.17 | 895.77±3.43 | 1093.82±3.47 |
| 2-Ethyl-3,6-dimethylpyrazine | 281.97±2.69 | 229.98±1.47 | 124.35±0.69 | 71.19±1.08 | 96.72±1.73 |
| 2,3,5,6-Tetramethylpyrazine | 5688.39±14.22 | 6702.12±36.4 | 4324.43±32.19 | 3030.94±14.29 | 4083.99±20.77 |
| 2,3-Diethylpyrazine | 14.11±0.57 | 33.13±1.17 | 38.92±1.24 | 38.64±2.11 | 46.43±1.78 |
| 2,3-Diethyl-5-methylpyrazine | 106±1.71 | 79.97±1.8 | 84.98±1.45 | 98.81±0.9 | 105.46±1.21 |
| 2-Isobutyl-3-methylpyrazine | 127.78±0.84 | 112.19±0.34 | 80.41±0.73 | 78.05±1.53 | 149.92±1.32 |
| (Trimethylpyrazin-2-yl)methanol | 8.99±0.09 | 10.02±0.08 | 2.85±0.09 | 1.46±0.03 | 2.64±0.03 |
| Dimethyl trisulfide | 205.93±8.07 | 147.14±4.98 | 11.61±1.27 | 15.31±0.37 | 16±0.7 |
| Phenol | 693.54±34.5 | 707.35±10.63 | 1633.32±35.76 | 373.17±6.22 | 857.76±92.14 |
| p-Cresol | 293.66±27.33 | 313.77±14.86 | 446.19±51.36 | 301.32±26.56 | 414.24±3.09 |
| Guaiacol | 261.22±19.58 | 325.98±38.44 | 195.25±5.41 | 200.58±9.05 | 391.41±44.16 |
| 4-Methylguaiacol | 52.25±6.23 | 62.9±1.66 | 83.27±0.47 | 51.24±3.17 | 72.11±6.77 |
| 4-Ethylguaiacol | 159.73±1.8 | 155.27±3.78 | 467.83±46.26 | 184.53±5.81 | 157.52±11.68 |
| 4-Vinylguaiacol | 1.48±0.18 | 3.08±0.12 | 2.74±0.04 | 12.72±0.22 | 2.53±0.07 |
| gama-Nonalactone | 227.09±22.81 | 255.67±8.91 | 333.86±40.73 | 249.44±27.17 | 360.64±24.77 |
| gama-Dodecalactone | 323.68±22.88 | 453.15±9.42 | 559.79±28.94 | 364.05±17.28 | 525.63±35.4 |
| Phytone | 87.37±5.22 | 86.41±0.67 | 61.32±6.81 | 61.49±7.16 | 81.87±5.31 |
| beta-Damascone | 12.32±0.9 | 14.86±0.2 | 5.29±0.43 | 4.61±0.19 | 3.72±0.42 |
| Geranylacetone | 44.46±2.44 | 45.57±3.71 | 19.13±1.03 | 39.9±2.04 | 43.65±5.34 |

Data represent mean ± SD (n = 3).

^a^The unit of compound is mg/L.

Table S5 The average content of 154 flavor compounds in EL town samples (μg/L)

| Name | EL-1 | EL-2 | EL-3 | EL-4 | EL-5 | EL-6 | EL-7 | EL-8 | EL-9 |
| --- | --- | --- | --- | --- | --- | --- | --- | --- | --- |
| Ethyl formate | 129888.21±1774.28 | 104339.22±1683.19 | 100536.05±3430.73 | 76430.03±821.02 | 134230.77±1010.59 | 149890.75±2062.73 | 163620.1±7474.51 | 89885.38±9026.25 | 106456.63±3000.71 |
| Ethyl acetate^a^ | 2352.52±74.92 | 2344.75±35.57 | 2547.08±147.99 | 2529.58±40.16 | 2780.25±7.83 | 2623.67±66.04 | 2774.44±70.65 | 2534.85±96.01 | 2380.07±118.54 |
| Propyl acetate | 5646.32±590.89 | 4354.29±260.44 | 5083.7±126.19 | 28383.65±844.82 | 3277.38±39.64 | 1234.68±22.63 | 1963.45±76.35 | 2743.19±15.23 | 704.65±29.64 |
| 3-Methylbutyl acetate | 6901.76±30.79 | 7627.87±34.41 | 8269.28±143 | 8649.29±298.61 | 7334.39±108.98 | 3367.39±65.09 | 3725.05±76.19 | 3136.63±75.61 | 3382.61±45.08 |
| Hexyl acetate | 442.33±18.35 | 358.82±18.13 | 232.9±19.06 | 777.47±38.59 | 336.72±9.16 | 194.48±17.05 | 44.26±2.39 | 274.42±24.26 | 173.8±2.07 |
| 2-Phenylethyl acetate | 20.62±1.13 | 17.77±0.3 | 28.87±2.34 | 19.16±1.13 | 31.33±1.67 | 0±0 | 0±0 | 0±0 | 0±0 |
| Ethyl propanoate | 58782.56±5703.04 | 35397.56±2227.7 | 36869.87±4480.32 | 78572.65±1175.72 | 44451.67±1117.62 | 62690.45±3979.39 | 63816.69±2685.49 | 54446.46±1992.49 | 64171.39±3180.63 |
| Ethyl 2-methylpropanoate | 15013.93±591.08 | 10939.54±72.87 | 11062.76±884.49 | 14531.5±517.11 | 13718.74±579.12 | 16177.16±685.02 | 17759.11±1159.45 | 9897.83±369.45 | 13244.77±995.54 |
| Ethyl butanoate | 48385.34±1813.1 | 39967.71±735.91 | 40198.78±2544.96 | 60465.75±1562.6 | 67208.72±772.83 | 67784.08±3122.3 | 65033.07±2746.9 | 101407.13±4835.26 | 76372.8±4880.71 |
| Butyl butanoate | 3.1±0.17 | 16.45±0.77 | 16.72±1.19 | 59.62±1.68 | 806.58±83.16 | 24.6±1.68 | 17.98±0.67 | 10.02±0.25 | 10.64±0.1 |
| 3-Methylbutyl butanoate | 461.65±27.61 | 460.15±27.01 | 9.52±0.18 | 445.67±9.06 | 49.64±0.57 | 7.76±0.66 | 12.7±0.44 | 314.41±11.82 | 245.18±25.11 |
| 2-Phenethyl buanoate | 118.99±5.49 | 156.14±18.52 | 184.93±7.87 | 356.92±14.45 | 354.28±21.52 | 110.09±1.47 | 153.15±0.84 | 408.42±9.95 | 118.79±4.16 |
| Ethyl 2-methylbutanoate | 7470.19±236.5 | 5032.95±356.08 | 4273.44±312.58 | 6863.42±190.11 | 5391.23±221.66 | 7928.08±304.12 | 8749.96±349.43 | 4539.92±63.46 | 6359.1±391.77 |
| Ethyl 3-methylbutanoate | 21439.67±497.44 | 14158.13±329.88 | 13693.67±612.64 | 18685.8±488.75 | 17957.65±105.93 | 23870.92±878.78 | 28321.76±1139.66 | 13172.93±606.9 | 17782.44±1211.07 |
| Ethyl pentanoate | 12623.64±1141.07 | 14887.85±390.23 | 7598.11±521.41 | 4856.22±179.18 | 10049.76±177.74 | 6814.37±772.77 | 2664.85±266.02 | 3475.54±29.37 | 7457.11±363.61 |
| 3-Methylbutyl pentanoate | 443.04±34.58 | 1.39±0.09 | 540.31±6.6 | 406.1±12.06 | 78.71±9.6 | 337.3±29.04 | 341.03±18 | 617.96±19.21 | 139.83±14.66 |
| Ethyl 4-methylpentanoate | 191.21±7.91 | 464.25±28.71 | 115±13.1 | 75.21±2.56 | 100.99±8.16 | 95.61±1.95 | 65.88±6.01 | 71.29±1.57 | 129.55±15.78 |
| Ethyl hexanoate | 92079.05±1221.87 | 77710.27±295.68 | 76040.59±1695.6 | 123955.09±780.99 | 49128.7±101.35 | 48860.13±35.2 | 37933.7±349.69 | 86560.11±1769.94 | 63374.96±2081.38 |
| Propyl hexanoate | 1458.86±153.82 | 1037.48±118.12 | 1022.63±49.44 | 380.33±7.09 | 679.64±51.78 | 116.32±6.84 | 0±0 | 1304.12±88.86 | 366.35±31.72 |
| Butyl hexanoate | 120.27±2.21 | 90.46±5.25 | 56.86±3.52 | 2.8±0.11 | 134.34±4.66 | 47.56±4.76 | 50.58±0.89 | 20.43±0.14 | 62±5.74 |
| 2-Methylpropyl hexanoate | 9.13±0.11 | 9.45±0.39 | 9.41±0.38 | 9.24±0.16 | 169.87±5.71 | 9.29±0.04 | 10.93±0.14 | 76.28±5.1 | 52.97±1.31 |
| 3-Methylbutyl hexanoate | 714.34±52.18 | 60220.54±7517.72 | 477.92±15.32 | 1101.59±82.2 | 238793.67±15060.78 | 347.12±16.86 | 234.38±1.26 | 4550.39±303.82 | 653.82±38.79 |
| Hexyl hexanoate | 569.51±3.46 | 743.94±8.75 | 858.66±99.38 | 1915.58±140.04 | 11836.56±851.76 | 230.18±7.12 | 236.26±11.5 | 2857.94±94.8 | 559.71±29.13 |
| 2-Phenethyl hexanoate | 5163.11±355.04 | 5696.71±422.18 | 9889.62±931.37 | 3319.59±112.03 | 4845.78±347.83 | 1038.98±10.09 | 971.89±9.02 | 8410.55±253.04 | 2460.59±93.37 |
| Ethyl 5-methylhexanoate | 7.54±0.41 | 8.88±0.56 | 29.07±2.08 | 15.25±0.63 | 55.65±1.88 | 15.47±1.42 | 28.64±0.68 | 6.42±0.29 | 9.15±0.7 |
| Ethyl heptanoate | 1405.39±105.69 | 1726.46±95.32 | 1118.54±57.03 | 764.97±27 | 231.14±10.75 | 418.35±29.39 | 86.03±8.29 | 1541.53±99.87 | 1922.33±16.25 |
| Ethyl octanoate | 1615.05±25.98 | 1975.79±124.84 | 1975.78±90.29 | 441.51±4.95 | 448.02±35.09 | 841±70.17 | 1002.16±38.04 | 1626.48±146.48 | 2499.97±49.62 |
| Ethyl nonanoate | 623.15±70.39 | 660.61±34.17 | 536.54±22.08 | 933.03±22.51 | 526.16±12.12 | 269.62±25.93 | 342.62±8.36 | 641.18±23.75 | 658.01±29.87 |
| Ethyl decanoate | 1618.94±26.48 | 501.43±23.59 | 149.81±11.68 | 324.25±18.26 | 389.44±5.53 | 510.75±63.56 | 3.04±0.02 | 76±3.28 | 592.16±5.8 |
| (E)-Ethyl 4-decenoate | 36.17±1.88 | 74.16±5.76 | 30.02±2.87 | 125.46±2.69 | 44.38±2.9 | 0±0 | 7.24±0.49 | 24.02±1.76 | 25.41±1.93 |
| Ethyl undecanoate | 3.43±0.2 | 10.55±0.4 | 3.33±0.11 | 3.88±0.11 | 4.64±0.09 | 4.44±0.34 | 3.16±0.26 | 3.61±0.28 | 6.33±0.14 |
| Ethyl dodecanoate | 239.69±5.89 | 234.59±8.39 | 229.14±9.82 | 243.71±13.36 | 247.64±16.29 | 317.15±26.72 | 299.65±3.77 | 280.84±23.99 | 301.21±4.63 |
| Ethyl tridecanoate | 32.31±1.89 | 30.88±1.21 | 35.95±1.41 | 36.9±1.07 | 38.22±2.82 | 24.11±0.71 | 27.41±2.26 | 28.86±0.97 | 31.99±1.42 |
| Ethyl tetradecanoate | 1045.81±83.01 | 1135.44±9.55 | 944.23±67.99 | 1428.14±54.41 | 1149.1±59.22 | 1155.99±88.24 | 993.81±41.23 | 1274.12±125.71 | 62.26±1.44 |
| Ethyl pentadecanoate | 243.19±29.1 | 288.31±5.64 | 185.93±15.2 | 246.64±3.24 | 230.55±10.56 | 234.42±22.89 | 199.25±16.65 | 209.41±3.38 | 5.07±0.04 |
| Ethyl hexadecanoate | 37663.87±492.14 | 31784.1±624.46 | 36846.06±526.5 | 34303.18±738.48 | 37487.31±904.4 | 38078.38±1095.29 | 42052.82±1208.34 | 26056.01±657.55 | 33174.78±1164.1 |
| Ethyl 9-hexadecenoate | 1544.28±32.43 | 1537.04±26.32 | 1858.16±7.63 | 1910.28±125.68 | 1742.03±31.37 | 1951.1±54.72 | 2376.79±35.67 | 1161.61±1.65 | 1437.62±29.81 |
| Ethyl heptadecanoate | 153.38±17.8 | 143.26±8.82 | 129.02±7.05 | 117.1±9.82 | 1045.94±42.97 | 127.62±7.84 | 173.3±7.83 | 121.4±1.65 | 163.6±2.07 |
| Ethyl octadecanoate | 170.56±17.53 | 165.44±6.32 | 149.39±7.04 | 133.97±0.17 | 372.39±21.44 | 116.55±6.99 | 153.79±4.18 | 119.06±2.56 | 141.6±11.04 |
| Ethyl oleate | 12757.28±316.37 | 10996.95±276.53 | 12871.9±391.28 | 12244.79±512.37 | 12572.11±233.15 | 12478.22±469.17 | 14315.58±492.17 | 7936.54±301.13 | 10956.02±715.64 |
| Ethyl linoleate | 11946.17±401.83 | 13260.52±304.57 | 15995.61±290.8 | 14780.37±686.04 | 12333.31±285.79 | 9293.21±601.63 | 9822.98±868.65 | 7096.02±94.37 | 9905.48±569.56 |
| Ethyl linolenate | 146.13±5.76 | 235.96±15.94 | 230.53±27.86 | 152.11±1.51 | 1298.83±13.67 | 45.86±4.01 | 48.69±3.54 | 92.94±0.64 | 142.69±11.71 |
| Diethyl butanedioate | 2022±207.1 | 1993.17±59.45 | 1899.57±211.18 | 1987.49±51.61 | 1198.23±6.29 | 1271.76±118.38 | 147.66±13.14 | 1234.71±63.9 | 1545.62±73.59 |
| Diethyl azeleate | 825.34±13.57 | 1202.11±10.78 | 357.72±9.35 | 898.7±53.38 | 565.78±19.46 | 1607.4±88.83 | 1724.41±19.75 | 1188.51±4.23 | 116.43±1.28 |
| Ethyl lactate^a^ | 2223.81±40.72 | 1867.23±85.22 | 2037.41±36.42 | 1447.06±53.95 | 2124.14±16.24 | 2122.57±97.37 | 1814.25±70 | 1265.56±84.58 | 2292.31±111.46 |
| 3-Methylbutyl lactate | 993.75±11.22 | 1613.79±142.29 | 1350.14±72.56 | 3223.49±36.47 | 1164.65±87.13 | 2100.55±191.72 | 2381.26±31.02 | 1482.4±117.96 | 1008.23±94.94 |
| Ethyl 2-furancarboxylate | 1465.69±24.72 | 2507.3±105.91 | 550.15±44.89 | 3745.88±47.11 | 406.77±19.07 | 6439.14±393.66 | 7213.91±403.56 | 3465.93±217.23 | 6094.65±185.11 |
| Ethyl 2-hydroxy-4-methylpentanoate | 11598.72±346.15 | 8806.37±359.78 | 9494.01±77.36 | 9021.23±700.51 | 12357.43±348.17 | 10243.85±596.34 | 10379.41±777.67 | 5159.04±385.38 | 8211.2±648.51 |
| Ethyl 2-hydroxy-hexanoate | 15703.27±807.12 | 15608.05±143.03 | 16059.93±1933.83 | 27330.08±658.89 | 14361.29±99.75 | 22339.04±833.47 | 23008.6±2746.73 | 13686.18±1660.88 | 14511.28±1066.18 |
| Ethyl benzoate | 14341.18±653.53 | 6126.21±141.83 | 9423.94±410.72 | 11527.02±1182.55 | 7652.34±220 | 15537.06±1722.12 | 27184.46±1000.97 | 13558.54±253.85 | 14031.7±199.41 |
| Ethyl phenylacetate | 1839.22±20.51 | 1336.05±87.95 | 2764.41±140.06 | 2914.92±88.62 | 3569.68±180.14 | 1647.31±46.89 | 1617.38±61.38 | 1160.58±113.17 | 1289.29±128.66 |
| Ethyl 3-phenylpropionate | 35.67±2.33 | 155.33±5.05 | 21.81±1.56 | 44.77±3.31 | 53.83±1.72 | 31.4±2.23 | 18.01±1.18 | 53.42±4.82 | 184.44±13.66 |
| Methanol^a^ | 168.35±2.31 | 170.33±1.53 | 180.5±6.28 | 173.55±1.44 | 205.49±1.26 | 162.3±1.77 | 180.69±2.02 | 115.5±5.42 | 139.08±4.12 |
| 1-Propanol^a^ | 1614.64±26.72 | 1216.06±7.42 | 1365.9±45.52 | 5576.18±58.01 | 846.69±5.46 | 584.69±0.43 | 720.71±10.33 | 824.27±34.83 | 500.9±20.39 |
| 1-Butanol | 76710.66±992.62 | 91574.63±387.89 | 93817.27±2569.85 | 125303.75±1102.88 | 117051.76±213.4 | 80995±277.24 | 89684.08±1236.59 | 76328.12±2175.67 | 73244.16±2444.86 |
| 2-Butanol | 35790.86±913.18 | 23363.6±122.51 | 24648.35±1461.3 | 110127.94±914.22 | 21661.25±216.89 | 23340.14±97.46 | 22008.32±598.36 | 40406.6±1836 | 26835.86±1939.57 |
| 2-Methylpropanol^a^ | 218.82±3.79 | 199.48±0.48 | 215.88±7.86 | 227.83±1.8 | 158.99±0.38 | 195.17±0.11 | 211.38±3.65 | 111.5±3.2 | 173.31±7.51 |
| 1-Pentanol | 13631.85±275.99 | 12832.49±428.08 | 12689.51±460.23 | 17271.23±474.45 | 14211.83±137.79 | 13204.1±794.83 | 13132.91±744.62 | 11401.45±581.28 | 12757.28±821.67 |
| 2-Pentanol | 7668.31±219.69 | 4459.96±79.37 | 4109.51±212.54 | 6283.31±47.29 | 2278.32±58.63 | 2067.3±25.52 | 1566.01±77.16 | 1648.81±110.26 | 2471.78±98.46 |
| 2-Methylbutanol | 80285.57±2704.21 | 63280.32±2650.09 | 68468.87±1958.17 | 81230.82±4422.05 | 48346.52±1328.52 | 70806.76±6459.14 | 74972.97±5014.57 | 36953.9±778.18 | 59896.11±6290.7 |
| 3-Methylbutanol^a^ | 467.96±9.94 | 390.47±6.96 | 417.64±12.39 | 478.88±17.83 | 301.49±10.63 | 450.35±17.51 | 469.66±19.71 | 285.71±14.26 | 401.74±24.75 |
| 1-Hexanol | 27479.92±476.36 | 21928.52±540 | 20460.71±480.41 | 32987.37±1700.22 | 16773.77±98.26 | 12591.56±635.01 | 12792.63±806.69 | 9755.23±621.29 | 12152.52±1076.56 |
| 1-Heptanol | 430.7±4.79 | 370.36±13.82 | 669.84±13.74 | 392.77±30.79 | 1189.75±92.83 | 259.29±15.81 | 267.81±20.98 | 189.55±5.97 | 163.52±10.84 |
| 2-Heptanol | 3467.84±287.95 | 24208.66±1483.64 | 8449.28±408.04 | 3471.64±385.65 | 19989.1±722.69 | 5474.08±226.53 | 4530.16±234.76 | 4813.79±511.4 | 7858.63±436.09 |
| 1-Octanol | 4.37±0.34 | 418.41±19.36 | 612.22±48.8 | 1693.31±86.11 | 496.79±11.87 | 646.69±46.64 | 620.38±16.48 | 632.64±26.22 | 644.62±21.05 |
| 1-Octen-3-ol | 183.75±2.9 | 178.16±7.15 | 204±12.04 | 301.69±25.2 | 1080.01±33.66 | 143.95±10.17 | 246.61±8.79 | 147.82±1.84 | 129.08±10.63 |
| 3-Octanol | 63.67±3.88 | 108.93±3.74 | 73.55±3.14 | 65.36±2.1 | 64.92±2.27 | 51.27±5.8 | 73.6±4.25 | 39.88±4.04 | 64.75±4.32 |
| 1-Nonanol | 17.68±0.81 | 127.46±11.7 | 7.96±0.6 | 101.48±8.48 | 26.1±0.89 | 7.25±0.11 | 44.68±3.58 | 59.78±2.99 | 91.71±4.47 |
| 2-Nonanol | 16.45±1.31 | 21.82±0.63 | 2.39±0.24 | 50.97±1.8 | 15.38±0.92 | 29.61±2.57 | 32.32±0.5 | 26.26±2.46 | 15.4±0.37 |
| (E)-2-Nonen-1-ol | 393.85±27.39 | 271.76±21.12 | 330.76±28.52 | 848.2±34.35 | 21379.48±889.01 | 236.46±24.16 | 186.3±16.72 | 356.52±2.28 | 446.42±30.53 |
| 1-Decanol | 1.7±0.09 | 3.53±0.1 | 1.28±0.09 | 4.88±0.47 | 1.91±0.11 | 0±0 | 0.55±0.06 | 0±0 | 0±0 |
| 2-Undecanol | 41.64±1.73 | 37.31±2 | 10.18±0.54 | 19.36±1.16 | 29±1.07 | 7.43±0.29 | 15.46±0.24 | 7.34±0.51 | 13.69±0.67 |
| 1,2-Propanediol | 31829.64±423.82 | 22629.11±398.42 | 24112.32±155.5 | 22044.21±1832.74 | 27067.67±892.95 | 20683.06±158.65 | 21638.51±476.69 | 27865.66±640.98 | 20012.4±2194.15 |
| (R,R)-2,3-Butanediol | 44306.07±3367.14 | 41229.89±2471.21 | 44649.71±3574.79 | 39016.41±3234.35 | 45737.18±2430.68 | 52775.15±424.52 | 48040.49±4343.79 | 54163.45±3017.33 | 65203.17±3351.27 |
| Meso-2,3-Butanediol | 7667.8±684.79 | 9225.4±365.68 | 10128.43±571.38 | 8706.14±232.06 | 11140.11±236.22 | 8613.28±128.83 | 7646.03±535.78 | 10807.18±312.65 | 10574.04±1125.72 |
| Benzyl alcohol | 445.02±29.25 | 1264.25±6.84 | 516.89±55.72 | 713.19±71.7 | 87.82±4.7 | 479.24±27.83 | 1355.44±155.57 | 5539.77±199.1 | 639.31±36.3 |
| 2-Phenethyl alcohol | 8754.36±191.91 | 7844.94±367.02 | 8372.24±118.48 | 8238.55±295.64 | 8165.68±331.07 | 7541.91±361.62 | 8020.77±294.91 | 8761.28±549.77 | 7491.26±392.05 |
| Formic acid^a^ | 229.43±0.96 | 191.02±1.24 | 176.99±0.26 | 135.06±0.49 | 213.61±0.83 | 214.26±2.23 | 218.39±2.03 | 150.66±1.4 | 189.63±1.24 |
| Acetic acid^a^ | 1604.65±1.06 | 1741.77±1.41 | 1726.47±6.44 | 1603.83±2.03 | 1821.37±1.22 | 1756.14±3.65 | 1881.19±5.22 | 1928.67±1.9 | 1607.67±2.35 |
| Propionic acid | 68720.65±488.6 | 53516.14±431.66 | 52982.07±488.19 | 76931.42±39.36 | 60153.05±34.08 | 69910.57±144.31 | 72030.25±173.47 | 63732.21±488.28 | 67582.68±321.63 |
| Butanoic acid | 26657.51±21.24 | 25243.26±76.1 | 24733.27±250.49 | 25690.45±78.55 | 25814.03±84.92 | 26830.24±87.86 | 28296.12±29.35 | 23444.3±229.26 | 24823.92±87.07 |
| 2-Methylpropanoic acid | 45329.12±226.9 | 40969.86±111.61 | 38120.05±117.29 | 48597.68±79.43 | 55758.99±64.31 | 61795.7±61.71 | 63921.79±133.27 | 66103.91±192.82 | 62104.32±43.82 |
| Pentanoic acid | 90569.49±364.71 | 85973.34±242.15 | 82113.03±666.35 | 91219.3±464.03 | 93749.92±122.36 | 90186.08±191.96 | 99505.92±209.05 | 83314.52±387.91 | 81355.08±173.81 |
| 3-Methylbutanoic acid | 25303.39±104.27 | 23775.98±41.8 | 22931.79±16.25 | 28397.48±41.53 | 22490.46±133.39 | 20959.86±23.62 | 19109.38±79.91 | 30004.33±32.97 | 23719.97±22.04 |
| Hexanoic acid | 57737.93±202.53 | 51983.65±453.04 | 46906.1±148.12 | 76151.21±146.54 | 29307.5±90.34 | 28731.85±60.12 | 21914.71±173.09 | 59594.71±389.12 | 37709.13±327.66 |
| Octanoic acid | 22392.77±92.67 | 21447.22±101.45 | 21270.5±282.4 | 23107.79±28.76 | 20456.13±51.59 | 20236.9±19.95 | 19881.32±12.87 | 20583.7±21.77 | 21075.35±19.06 |
| Decanoic acid | 9393.37±152.26 | 9281.3±194.4 | 9281.91±154.03 | 9275.78±193.31 | 9399.56±112.61 | 9361.27±51.46 | 9580.5±45.05 | 9295.72±44.78 | 9367.89±87.61 |
| Dodecanoic acid | 2467.24±58.56 | 2296.95±61.82 | 2552.13±69.13 | 2279.43±10.85 | 2335.3±73.54 | 2291.49±35.76 | 2771.19±84.38 | 1467.19±53.02 | 1953.26±43.02 |
| Benzoic acid | 48578.47±3164.01 | 44888.48±4781.96 | 30245.23±1434.43 | 54024.72±6207.31 | 3651.77±259.34 | 42405.82±595.85 | 78772.17±6711.38 | 57528.69±48.74 | 65086.38±75.63 |
| L-Lactic acid^a^ | 1731.61±1.21 | 1596.36±0.98 | 1610.6±2.52 | 1254.22±1.79 | 1611.45±1.93 | 1620.09±1.12 | 1405.91±0.6 | 1102.72±1.01 | 1758.29±1.89 |
| Citric acid | 10312.73±51 | 57911.63±224.8 | 49230.07±203.35 | 10474.13±50.03 | 22935.8±270.48 | 76257.13±2324.89 | 165498.67±3871.37 | 31627.43±357.57 | 31297.03±845.18 |
| Palmitic acid | 16296.5±36.33 | 14002±75.93 | 15755±280.06 | 15223±141.25 | 16341±34.29 | 17696±19.6 | 19912.5±51.85 | 8708±22.05 | 13590.5±112.27 |
| Oleic acid | 5264±3.27 | 6165±45.72 | 6917.5±11.02 | 6903.5±28.17 | 5265.5±41.23 | 4189±1.63 | 4331.5±28.99 | 3465±35.11 | 4332±21.23 |
| Linoleic acid | 4381.5±13.47 | 4152±35.11 | 4498.5±29.8 | 4687.5±60.01 | 4269±17.15 | 4297.5±11.02 | 5166±0.82 | 2578.5±26.54 | 3605±13.88 |
| Acetaldehyde^a^ | 810.89±15.2 | 635.56±29.21 | 634.7±1.31 | 552.98±11.77 | 779.85±33.82 | 1049.03±8.09 | 1105.21±69.74 | 756.53±76.54 | 886.65±6.86 |
| 2-Methylpropanal | 4656.92±258.53 | 7405.94±610.69 | 8472.94±480.06 | 6662.64±602.38 | 8528.11±117.76 | 3992.87±106.82 | 4284.29±308.95 | 2922.42±273.09 | 3779.8±119.88 |
| 2-Methylbutanal | 2469.16±87.03 | 4781.57±115.03 | 6004.68±371.14 | 5649.95±216.48 | 8077.98±115.33 | 1774.67±157.74 | 2212.43±13.8 | 745.5±26.78 | 1756.66±30.48 |
| 3-Methylbutanal | 23605.13±595.39 | 29312±270.6 | 33608.51±1282.74 | 37820.01±943.76 | 43567.45±292.18 | 22985.36±835.96 | 26088.42±219.88 | 14388.06±1087.98 | 20552.29±754.89 |
| Nonanal | 26.24±2.04 | 44.69±3.53 | 43.22±3.84 | 93.54±3.2 | 337.58±26.79 | 76.71±0.99 | 108.86±0.76 | 39.29±1.96 | 74.14±8.72 |
| Benzaldehyde | 1322.17±143.57 | 1890±197.24 | 1933.51±160.99 | 1632.57±144.83 | 2282.86±70.87 | 1466.56±71.4 | 1458.19±34.49 | 807.31±77.01 | 1013.43±36.12 |
| Benzeneacetaldehyde | 15862.2±123.22 | 39339.76±4269.8 | 57648.65±5963.56 | 48625.19±2094.33 | 16337.16±445.1 | 17128.63±873.88 | 16988.27±223.81 | 9565.53±154.22 | 24624.94±1051.91 |
| Diethoxymethane | 22396.04±871.3 | 13047.86±227.56 | 13095.84±771.07 | 8460.07±225.91 | 15664.38±255.57 | 11783.55±375.28 | 14441.16±542.06 | 11124.6±502.83 | 8995.1±558.46 |
| 1,1-Diethoxyethane | 590570.84±22425.26 | 454826.65±11499.74 | 449242.28±35889.38 | 382673.26±8970.1 | 566153.13±14701.3 | 702394.06±6243.2 | 763190.36±32215.65 | 520569.03±15946.35 | 615411.54±35152.87 |
| 1,1-Diethoxy-3-methylbutane | 14819.51±610.47 | 18533.72±1422.7 | 21297.04±1841.19 | 23190.08±1523.01 | 27714.86±2962.05 | 13410.04±338.38 | 15075.2±1247.09 | 7384.76±287.88 | 12440.41±261.25 |
| (2,2-Diethoxyethyl)-benzene | 19.97±1.2 | 56.72±0.7 | 159.66±4.76 | 343.61±19.63 | 55.69±2.34 | 21.31±0.69 | 22.74±0.87 | 60.59±1.68 | 79.18±5.13 |
| 1,1,3,3-Tetraethoxy-propane | 72.96±5.17 | 153.77±15.56 | 34.36±0.62 | 57.19±3.38 | 43.25±4.37 | 28.61±0.63 | 16.5±0.64 | 37.85±2.78 | 39.54±2.46 |
| Acetone | 21449.85±1132.72 | 21040.9±954.8 | 22326.57±1855.87 | 23818.89±669.43 | 27924.51±311.92 | 21926.35±234.94 | 24109.94±1376.46 | 12963.27±205.23 | 17650.02±1671.77 |
| 2-Pentanone | 24116.8±1121 | 14744.3±565.62 | 14162.22±704.51 | 18543.28±655.99 | 14604.19±308.48 | 9266.17±26.14 | 6153.7±29.2 | 5698.97±33.87 | 10659.88±42.15 |
| 3-Hydroxyl-2-butanone | 986.15±48.67 | 6284.96±357.55 | 8380.98±262.77 | 405.37±10.69 | 11372.63±601.9 | 0±0 | 0±0 | 0±0 | 0±0 |
| 2-Heptanone | 79.67±5.42 | 448.2±31.63 | 650.35±8.94 | 710.28±28.77 | 4814.07±403.84 | 7.01±0.54 | 50.95±0.32 | 2006.92±16.48 | 2126.34±72.64 |
| 2-Octanone | 0.56±0.02 | 0.59±0.04 | 0.61±0.01 | 0.83±0.02 | 0.55±0.05 | 0.89±0.04 | 0.97±0.08 | 0.9±0.05 | 0.61±0.04 |
| 3-Octanone | 24.07±0.95 | 49.47±5.94 | 69.4±2.42 | 50.29±1.08 | 268.49±6.29 | 39.62±2.61 | 69.23±5.24 | 37.09±2.06 | 39.14±2.97 |
| 2-Nonanone | 107.27±12.24 | 125.2±4.27 | 56.63±3.01 | 195.31±6.45 | 67.36±0.97 | 83.2±2.01 | 59.18±6.67 | 134.13±10.11 | 143.28±2.57 |
| 4-Nonanone | 84.47±5.13 | 10.72±0.62 | 34.47±0.62 | 59.75±5.67 | 107.1±9.13 | 13.53±1.6 | 46.62±1.29 | 0±0 | 0±0 |
| 2-Undecanone | 37.45±3.87 | 40.38±2.92 | 28.19±2.01 | 49.69±2.15 | 29.96±0.44 | 13.02±1.3 | 6.41±0.13 | 21.48±1.6 | 23.75±2.55 |
| 4-Undecanone | 159.98±16.67 | 0±0 | 0±0 | 349.89±38.75 | 267.48±12.73 | 0±0 | 0±0 | 0±0 | 662.93±14.08 |
| 2-Tridecanone | 6.28±0.43 | 12.6±0.52 | 11.84±1.46 | 16.54±0.48 | 19.65±0.38 | 11.03±0.47 | 5.61±0.27 | 12.78±1.17 | 17.77±1.33 |
| 2-Pentadecanone | 47.78±4.75 | 44.05±1.81 | 48.61±3.59 | 76.53±4.24 | 49.64±2.29 | 38.51±3.01 | 30.98±1.17 | 50.58±4.41 | 6.58±0.09 |
| Furfuryl alcohol | 4128.44±93.23 | 7546.47±92.2 | 15351.6±300.02 | 10485.06±360.5 | 10115.56±133.13 | 3326.73±14.37 | 3243.99±0 | 5161.68±156.86 | 3602.97±36.16 |
| Furfural^a^ | 193.54±4.78 | 203.3±16.7 | 218.91±14.24 | 179.91±13.94 | 253.07±16.71 | 220.92±8.36 | 269.5±17.47 | 156.68±16.89 | 204.1±15.76 |
| 5-Methylfurfural | 584.05±67.27 | 453.26±11.09 | 403.71±48.73 | 798.97±98.19 | 2100.56±165.75 | 410.35±12.18 | 749.28±50.55 | 552.19±6.65 | 314.16±24.45 |
| Furfuryl ethyl ether | 3528.47±295.92 | 5763.44±42.94 | 6600.12±250.59 | 6204.51±127.17 | 8378.74±108.29 | 2008.12±104.93 | 1617.02±47.21 | 2889.15±136.87 | 2561.53±91.73 |
| 2-Acetylfuran | 846.28±6.89 | 974.51±43.87 | 837.95±43.05 | 1439.58±126.17 | 3589.9±117.08 | 776.62±35.55 | 1207.9±52.89 | 831.95±22.06 | 940.13±20.86 |
| HDMF | 88.23±0.65 | 35.08±2.31 | 47.83±1.74 | 45.58±1.17 | 73.48±3.28 | 60.99±2.7 | 42.14±1.22 | 69.44±0.5 | 54.68±0.59 |
| HEMF | 10.95±0.65 | 32.68±1.06 | 1.45±0.12 | 0±0 | 5.04±0.15 | 12.02±1.37 | 9.98±0.15 | 0.61±0.11 | 1.24±0.13 |
| Sotolon | 79.36±1.82 | 43.24±0.79 | 21.41±0.49 | 19.35±0.88 | 71.12±0.53 | 55.42±0.73 | 67.68±1.99 | 64.58±3 | 77.47±1.5 |
| Pyrazine | 120.42±2.95 | 140.85±2.97 | 124.59±4.13 | 98.15±4.49 | 154.81±2.97 | 152.52±1.81 | 130.09±2.59 | 114.97±3.11 | 157.06±3.91 |
| 2-Methylpyrazine | 585.68±9.01 | 867.07±33.96 | 751.04±24.24 | 680.7±22.06 | 857.01±0.81 | 565.84±9.53 | 594.42±24.27 | 463.34±12.13 | 586.56±13.51 |
| 2,3-Dimethylpyrazine | 102.03±3.45 | 123.27±4.1 | 110.66±2.85 | 77.27±4.24 | 147.88±4.52 | 68.25±7.76 | 76.39±6.16 | 66.23±4.5 | 86.41±5.64 |
| 2,5-Dimethylpyrazine | 2097.11±156.9 | 3142.53±118.83 | 2874.02±157.85 | 3007.67±164.5 | 5578.93±39.61 | 1424.79±82.21 | 1783.38±65.43 | 1506.04±48.69 | 1517.55±110.08 |
| 2,6-Dimethylpyrazine | 377.69±11.35 | 480.3±7.4 | 376.92±2.89 | 426.91±10.63 | 547.86±6.38 | 297.07±2.01 | 469.54±2.98 | 300.65±9.44 | 336.55±4.94 |
| 2-Ethylpyrazine | 215.97±13.37 | 278.53±14.22 | 220.37±13.11 | 260.3±6.48 | 290.98±7.66 | 179.8±8.75 | 251.37±15.93 | 125.33±5.06 | 230.32±13.07 |
| 2-Ethyl-3-methylpyrazine | 58.16±1.51 | 62.81±0.86 | 52.63±0.75 | 39.58±2.76 | 84.04±0.22 | 60.62±1.05 | 73.58±4.62 | 50.83±1.06 | 60.17±1.81 |
| 2-Ethyl-6-methylpyrazine | 2827.28±195.09 | 4071.21±87.75 | 3812.65±250.28 | 4896.97±270.94 | 4515.93±21.83 | 2552.71±150.71 | 2800.67±74.37 | 1777.88±140.09 | 2944.4±128.68 |
| 2,3,5-Trimethylpyrazine | 1696.06±123.99 | 2286.87±123.08 | 2041.28±130.36 | 1837.2±72.75 | 3671.91±44.14 | 1170.61±67.26 | 1367.5±102.06 | 1479.61±150.36 | 1490.68±115.87 |
| 2-Ethyl-3,5-dimethylpyrazine | 1814.24±121.01 | 2230.51±120.27 | 2211.3±146.17 | 2133.24±141.34 | 4105.78±151.55 | 2445.15±180.22 | 2188.32±189.95 | 1719.52±44.63 | 3302.59±229.93 |
| 2-Ethyl-3,6-dimethylpyrazine | 263.04±2.27 | 417.26±3.13 | 351.64±7.22 | 382.24±12.35 | 582.95±8.96 | 255.64±11.32 | 277.96±5.88 | 204.55±4.23 | 310.8±9.67 |
| 2,3,5,6-Tetramethylpyrazine | 1964.25±145.37 | 2517.09±170.41 | 2471.54±164.44 | 982.72±71 | 4353.69±95.9 | 1041.79±85.05 | 1136.33±65.59 | 2253.99±180.05 | 1710.02±87.99 |
| 2,3-Diethylpyrazine | 27.13±0.45 | 78.02±4.43 | 48.73±0.94 | 39.49±1.23 | 28.9±2.06 | 58.97±1.26 | 70.46±2.19 | 37.83±0.82 | 34.4±2.36 |
| 2,3-Diethyl-5-methylpyrazine | 185.96±3.75 | 271.72±3.77 | 238.13±4.88 | 207.27±6.55 | 251.16±6.83 | 184.22±9.06 | 270.54±11.1 | 202.92±5.11 | 282.24±6.7 |
| 2-Isobutyl-3-methylpyrazine | 127.19±5.66 | 170.76±11.63 | 149.93±14.85 | 121.82±8.6 | 138.46±7.28 | 126.86±14.05 | 191.82±10.84 | 124.95±9.84 | 165.27±14.63 |
| (Trimethylpyrazin-2-yl)methanol | 0±0 | 3.72±0.13 | 5.53±0.09 | 0±0 | 11.89±0.21 | 0±0 | 0±0 | 9.79±0.81 | 13.99±1.09 |
| Dimethyl trisulfide | 27.69±0.33 | 27.82±0.56 | 27.35±0.15 | 19.06±0.29 | 28.28±0.3 | 28.62±0.85 | 30.62±0.57 | 28.22±0.29 | 27.47±0.25 |
| Phenol | 482.88±15.2 | 442.76±30.23 | 621.92±4.3 | 598.9±18.35 | 867.41±29.2 | 402.03±13.51 | 654.37±30.68 | 739.02±40.02 | 534.29±35.51 |
| p-Cresol | 811.56±27.12 | 553.09±17.97 | 531.17±29.05 | 704.09±7.75 | 1175.27±27.22 | 1259.9±2.43 | 1971.4±45.48 | 1045.49±14.05 | 844.17±34.94 |
| Guaiacol | 787.44±15.41 | 947.26±2.47 | 2238.63±84.47 | 722.96±1.82 | 620.69±9.22 | 207.63±3.23 | 104.57±11.21 | 955.22±5.37 | 605.31±6.97 |
| 4-Methylguaiacol | 17.71±0.42 | 20.48±1.04 | 18.38±0.46 | 60.47±1.55 | 54.91±1.25 | 17.92±0.5 | 19.97±0.24 | 26.07±0.29 | 18.12±2.17 |
| 4-Ethylguaiacol | 216.69±12.64 | 187.08±4.63 | 178.19±11.72 | 342.58±24.33 | 821.25±6.77 | 677.76±6.16 | 810.07±25.71 | 352.15±12.43 | 376.73±1.67 |
| 4-Vinylguaiacol | 8.39±0.22 | 6.86±0.59 | 8.95±0.01 | 1.31±0 | 9.07±0.28 | 1.03±0.03 | 1.44±0.01 | 0.95±0.03 | 0.78±0.03 |
| gama-Nonalactone | 84.77±0.89 | 140.36±15.38 | 127.12±10.68 | 246.77±6.19 | 76.3±5.15 | 179.63±0.6 | 214.09±1.37 | 266.22±12.66 | 136.71±12.34 |
| gama-Dodecalactone | 373.38±21.44 | 334.43±14.57 | 353.43±16.74 | 423.28±3.63 | 566.54±28.79 | 494.57±0.53 | 628.7±1.78 | 523.41±2.69 | 473.93±13.2 |
| Phytone | 53.91±0.9 | 67.19±2.37 | 54.12±4.8 | 55.84±1.75 | 65.99±3.39 | 47.86±5.11 | 51.6±4.38 | 65.01±7.82 | 243.64±9.44 |
| beta-Damascone | 1±0.05 | 2.97±0.3 | 2.35±0.24 | 3.28±0.35 | 448.2±15.56 | 0.75±0 | 0.5±0 | 0.89±0.01 | 0.85±0.02 |
| Geranylacetone | 7.53±0.89 | 18.24±2.18 | 11.34±1.04 | 10.64±0.5 | 25.15±0.37 | 0±0 | 0±0 | 5.44±0.1 | 9.82±1.15 |

Data represent mean ± SD (n = 3).

^a^The unit of compound is mg/L.

Table S6 The average content of 154 flavor compounds in TP town samples (μg/L)

| Name | TP-1 | TP-2 | TP-3 | TP-4 | TP-5 | TP-6 |
| --- | --- | --- | --- | --- | --- | --- |
| Ethyl formate | 53027.89±2270.76 | 71850.22±1914.84 | 224811.33±2121.09 | 61297.09±2280.28 | 85715.39±2168.49 | 63539.1±3072.49 |
| Ethyl acetate^a^ | 2208.01±82.91 | 2195.83±42.06 | 2604.19±42.77 | 2324.8±49.66 | 2338.79±45.69 | 2233.99±89.07 |
| Propyl acetate | 6229.93±590.68 | 7160.74±185.95 | 7748.52±308.96 | 10881.6±509.16 | 13204.35±218.43 | 9388.21±694.89 |
| 3-Methylbutyl acetate | 6101.14±258.56 | 8102.36±227.38 | 3536.27±65.7 | 3466.67±71.19 | 3551.23±69.09 | 7124.32±181.83 |
| Hexyl acetate | 409.63±31.95 | 691.95±21.98 | 779.12±34.54 | 296.1±31.36 | 311.95±12.96 | 72.18±5.91 |
| 2-Phenylethyl acetate | 80.64±8.81 | 155.57±4.01 | 10.36±0.82 | 76.01±3.49 | 19.61±1.12 | 151.39±9.93 |
| Ethyl propanoate | 44693.31±4201.88 | 43369.94±1742.81 | 66498.57±2451.45 | 56018.87±3414.05 | 84622.36±1549.73 | 51658.21±1678.98 |
| Ethyl 2-methylpropanoate | 9935.8±767.28 | 10978.63±831.4 | 18853.26±353.36 | 14920.05±916.83 | 12802.82±662.52 | 13119.06±918.71 |
| Ethyl butanoate | 33554.83±1623.26 | 40959.27±1371.36 | 43819.05±1009.23 | 44810.35±1893.51 | 57100.26±2021.41 | 34815.32±1364.95 |
| Butyl butanoate | 32.52±2.42 | 124.98±11.7 | 13.21±1.19 | 68.14±3.5 | 18.29±0.53 | 54.39±4.55 |
| 3-Methylbutyl butanoate | 234.63±14.95 | 412.61±48.86 | 245.74±10.56 | 270.61±13.85 | 292.01±11.21 | 10.53±0.6 |
| 2-Phenethyl buanoate | 495.7±4.78 | 622.35±9.32 | 177.32±3.43 | 376.51±6.94 | 345.15±10.07 | 462.47±35.11 |
| Ethyl 2-methylbutanoate | 1371.93±50.37 | 2706.37±178.48 | 9902.37±231.17 | 3053.87±68.17 | 3870.99±144.02 | 2212.67±130.75 |
| Ethyl 3-methylbutanoate | 7148.82±404.39 | 9732.27±398.11 | 30968.07±793.71 | 10965.66±591.26 | 12542.84±295.12 | 9313.87±329.47 |
| Ethyl pentanoate | 9644.31±317.01 | 10278.43±768.64 | 14393.56±445.98 | 8679.16±261.68 | 4731.45±188.52 | 4864.17±473.34 |
| 3-Methylbutyl pentanoate | 99.63±1.27 | 530.48±9.77 | 710.44±51.03 | 371.31±8.97 | 421.68±25.39 | 229.19±9.03 |
| Ethyl 4-methylpentanoate | 145.01±12.6 | 270.43±17.05 | 159.22±7.98 | 188.61±7.48 | 46.28±1.51 | 135.64±8.92 |
| Ethyl hexanoate | 51678.67±1144.02 | 57566.72±91.7 | 84521.9±631.6 | 53192.39±176.73 | 76277.41±847.59 | 44072.7±328.54 |
| Propyl hexanoate | 880.37±11.88 | 2693.74±32.11 | 3019.51±238.29 | 1439.02±46.37 | 1721.06±139.4 | 1301.39±75.19 |
| Butyl hexanoate | 53.07±6.5 | 165.71±0.04 | 113.15±9.87 | 84.65±10.02 | 94.3±2.71 | 79.63±6.22 |
| 2-Methylpropyl hexanoate | 12.48±0.95 | 0.29±0.01 | 115.36±9.47 | 40.15±0.91 | 99.62±6.96 | 45.25±1.73 |
| 3-Methylbutyl hexanoate | 1706.2±104.05 | 1761.01±68.08 | 1190.83±70.36 | 863.59±33.06 | 1259.79±102.33 | 30238.93±1777.66 |
| Hexyl hexanoate | 6836.93±707.51 | 7327.5±152.5 | 2572.43±285.87 | 3391.84±73.46 | 4592.12±205.29 | 4440.32±184.72 |
| 2-Phenethyl hexanoate | 19832.55±758.39 | 18315.52±638.41 | 8046.06±325.1 | 9035.18±208.74 | 12584.2±49.97 | 11538.3±687.44 |
| Ethyl 5-methylhexanoate | 8.96±0.37 | 7.92±0.66 | 14.27±0.4 | 12.3±0.74 | 6.22±0.31 | 3.13±0.36 |
| Ethyl heptanoate | 881.05±76.59 | 1698.86±132.06 | 1890.63±55.81 | 1720.11±100.46 | 1376.64±87.07 | 1179.49±25.61 |
| Ethyl octanoate | 1248.85±26.96 | 822.86±66.09 | 2614.94±202.95 | 2513.45±298.25 | 2751.19±123.14 | 1903.83±105.7 |
| Ethyl nonanoate | 292.77±23.54 | 804.41±7.55 | 1374.54±110.93 | 143.76±10.56 | 542.2±10.26 | 444.58±23.69 |
| Ethyl decanoate | 279.91±12.68 | 627.2±12.7 | 536.71±4.38 | 352.94±25.47 | 86.1±2.09 | 221.83±18 |
| (E)-Ethyl 4-decenoate | 51.88±4.96 | 84.85±2.94 | 73.83±3.22 | 85.16±6.38 | 8.4±0.69 | 57.88±1.8 |
| Ethyl undecanoate | 2.87±0.09 | 8.62±0.4 | 9.05±0.43 | 2.57±0.29 | 0±0 | 6.32±0.2 |
| Ethyl dodecanoate | 240.51±5.74 | 329.56±11 | 292.15±2.33 | 255.88±25.94 | 215.19±23.25 | 332.35±22.21 |
| Ethyl tridecanoate | 40.47±1.04 | 47.87±1.8 | 62.59±2.18 | 43.02±3.66 | 38.98±3.39 | 42.11±2.25 |
| Ethyl tetradecanoate | 1047.26±4.48 | 1339.22±51.95 | 1762.27±81.16 | 1189.02±115.28 | 860.99±58.42 | 1391.61±73.56 |
| Ethyl pentadecanoate | 181.4±4.56 | 257.02±10.52 | 363.04±11.64 | 219.14±17.67 | 172.43±21.41 | 244.15±10.4 |
| Ethyl hexadecanoate | 26366.03±1025.53 | 32869.65±860.76 | 27340.82±409.09 | 22081.52±362.94 | 23457.97±414.72 | 25998.84±413.84 |
| Ethyl 9-hexadecenoate | 1677.53±34.17 | 2390.01±14.84 | 1802.62±26.09 | 1421.67±14.15 | 1664.07±17.3 | 1726.96±19.28 |
| Ethyl heptadecanoate | 89.74±0.04 | 62.4±0.17 | 100.59±3.43 | 74.65±3.63 | 73.01±0.02 | 88.5±4.65 |
| Ethyl octadecanoate | 100.21±1.99 | 39.88±1.85 | 95.93±0.08 | 86.65±0.83 | 83.39±0.31 | 94.07±0.42 |
| Ethyl oleate | 11153.45±742.97 | 13914.57±445.78 | 9732.35±191.19 | 7542.33±259.32 | 8941.64±115.73 | 10496.77±126.95 |
| Ethyl linoleate | 11529.56±284.05 | 11213.68±856.37 | 8470.75±242.31 | 12856.91±167.01 | 13520.54±412.17 | 11306.95±307.59 |
| Ethyl linolenate | 914.51±51.4 | 553.9±9.07 | 92.03±4.26 | 693.28±48.04 | 582.84±2.54 | 999.07±5.36 |
| Diethyl butanedioate | 2360.14±16.63 | 2066.84±34.76 | 2349.89±89.11 | 1359.36±105.99 | 252.81±27.51 | 253.8±10.97 |
| Diethyl azeleate | 346.6±14.94 | 1288.42±145.21 | 2650.77±54.4 | 713.67±26.38 | 545.45±25.62 | 544.22±25.59 |
| Ethyl lactate^a^ | 1160.64±98.81 | 886.76±43.68 | 811.26±20.79 | 1338.84±58.8 | 952.58±48.25 | 1335.75±42.94 |
| 3-Methylbutyl lactate | 1761.03±27.94 | 1520.51±56.29 | 1592.13±14.89 | 2145.84±85.34 | 877.51±43.78 | 2475.63±108.88 |
| Ethyl 2-furancarboxylate | 489.38±33.78 | 571.36±13.38 | 4332.02±174.39 | 829.1±38.08 | 971.46±35.72 | 677.6±81.43 |
| Ethyl 2-hydroxy-4-methylpentanoate | 6065.86±420.83 | 7837.39±392.94 | 9429.54±430.09 | 9076.62±750.97 | 6665.98±244.5 | 8305.29±145.4 |
| Ethyl 2-hydroxy-hexanoate | 11548.48±228.24 | 11338.66±373.45 | 11396.24±56.75 | 20876.2±439.66 | 13280.88±1131.97 | 22387.48±824.9 |
| Ethyl benzoate | 12082.77±241.72 | 15633.44±1006.58 | 19987.37±1164.75 | 11562.21±262.9 | 14336.32±1683.19 | 8406.32±223.38 |
| Ethyl phenylacetate | 3174.92±287.73 | 2314.4±131.74 | 3578.3±54.9 | 3350.35±126.54 | 2283.39±114.35 | 4026.79±189.87 |
| Ethyl 3-phenylpropionate | 83.79±3.17 | 89.73±1.36 | 116.83±2.43 | 53.02±4.04 | 24.67±2.02 | 33.32±2.5 |
| Methanol^a^ | 140.61±5.1 | 188.55±1.1 | 238.78±0.79 | 185.21±1.42 | 178.12±3.24 | 171.06±2.69 |
| 1-Propanol^a^ | 1257.71±49.85 | 1728.65±16.64 | 1783.64±6.36 | 1855.13±9.2 | 2851.98±64.61 | 1859.13±29.01 |
| 1-Butanol | 67054.44±2079.12 | 87519.67±683.96 | 85301.36±291.16 | 87073.63±411.67 | 91797.86±2001.28 | 71388.19±696.59 |
| 2-Butanol | 25500.6±1460.29 | 43891.91±458.14 | 40028.87±168.53 | 56428.42±39.88 | 75842.84±1487.18 | 37621.84±1107.3 |
| 2-Methylpropanol^a^ | 157.9±5.43 | 201.98±1.21 | 253.29±0.65 | 191.84±0.84 | 188.36±2.98 | 168.34±2.83 |
| 1-Pentanol | 9870.15±415.94 | 14925.78±680.88 | 14696.91±665.5 | 12603.65±450.29 | 13368.47±395.12 | 11346.02±338.53 |
| 2-Pentanol | 1578±24.15 | 3937.15±94.16 | 5930.32±79.38 | 3192.55±45.1 | 3869.41±161.44 | 1880.43±123.72 |
| 2-Methylbutanol | 50281.34±5751.51 | 82927.73±3399.88 | 85587.07±1364.09 | 68335.69±2730.85 | 66195.53±2811.54 | 54839.04±4832.02 |
| 3-Methylbutanol^a^ | 285.67±23.89 | 406.19±16.75 | 401.76±14.35 | 388.02±16.37 | 361.43±14.14 | 322.85±10.63 |
| 1-Hexanol | 9104.88±906.24 | 17970.98±908.64 | 25350.55±875.6 | 14136.51±1004.28 | 18858.29±696.99 | 9170.23±421.35 |
| 1-Heptanol | 130.89±1.49 | 325.52±25.54 | 676.4±75.32 | 284.36±35.53 | 367.34±3.59 | 253.29±21.86 |
| 2-Heptanol | 2241.79±41.97 | 10184.42±335.17 | 11740.32±584.37 | 3447.72±166.36 | 6159.94±465.48 | 2287.67±198.1 |
| 1-Octanol | 413.83±22.07 | 647.94±29.29 | 1021.95±22.94 | 661.99±24.1 | 670.23±56.63 | 468.28±43.3 |
| 1-Octen-3-ol | 217.37±3.88 | 259.41±0.45 | 227.57±19.37 | 162.82±5.91 | 173.86±18.6 | 152.54±11.34 |
| 3-Octanol | 42.87±1.23 | 91.52±6.67 | 252.7±26.06 | 93.73±4.31 | 94.79±8.37 | 47.59±1.39 |
| 1-Nonanol | 35.96±2.36 | 62.6±2.67 | 101.48±1.54 | 81.89±4.26 | 51.12±1.61 | 4.57±0.39 |
| 2-Nonanol | 11.21±0.33 | 24.27±1.31 | 25.44±1.97 | 32.47±1.42 | 11.9±0.13 | 1.69±0.1 |
| (E)-2-Nonen-1-ol | 440.39±26.61 | 589.43±25.1 | 437.67±44.3 | 446.16±13.22 | 473.1±26.07 | 561.08±53.33 |
| 1-Decanol | 3.45±0.07 | 5.39±0.32 | 3.36±0.1 | 2.62±0.07 | 2.04±0.1 | 2.57±0.15 |
| 2-Undecanol | 27.43±2.04 | 47.96±1.15 | 60.68±0.29 | 36.61±3.63 | 11.88±0.42 | 20.41±1.61 |
| 1,2-Propanediol | 25024.93±2811.87 | 40162.13±2835.06 | 26811.71±660.6 | 30928.96±962.24 | 34520.84±791.97 | 27105.67±2050.86 |
| (R,R)-2,3-Butanediol | 49932.43±5853.19 | 48304.89±2924.91 | 43819.53±3504.22 | 47086.75±358.96 | 48832.98±2617.52 | 44798.22±3788.95 |
| Meso-2,3-Butanediol | 10976.49±355.72 | 11094.79±565.45 | 9238.16±393.56 | 9762.53±112.04 | 9813.02±254.64 | 10102.46±690.62 |
| Benzyl alcohol | 1172.46±20.49 | 977.02±45.03 | 896.99±68.38 | 555.56±26.64 | 1870.03±105.4 | 355.76±13.41 |
| 2-Phenethyl alcohol | 10033.45±866.44 | 10805.88±657.39 | 7496.09±245.57 | 10291.95±471 | 9271.16±419.19 | 10200.65±341.69 |
| Formic acid^a^ | 110.72±0.41 | 131.16±0.5 | 234.55±0.97 | 131.34±0.3 | 141.44±1.05 | 131.83±0.5 |
| Acetic acid^a^ | 1496.96±3.78 | 1497.37±3.75 | 1679.04±2.8 | 1539.77±6.76 | 1590.25±2.43 | 1429.31±0.87 |
| Propionic acid | 54039.06±670.73 | 52921.4±282.04 | 65211±378.37 | 62698.15±385.05 | 78970.08±447.45 | 56596.46±9.77 |
| Butanoic acid | 28200.51±30.22 | 25174.11±21.69 | 27722.65±74.02 | 29685.87±119.37 | 25217.6±143.76 | 29189.19±161.98 |
| 2-Methylpropanoic acid | 35537.41±169.45 | 38287.44±1054.82 | 43022.2±68.83 | 40305.9±152.16 | 49158.32±70.22 | 34457.1±567.65 |
| Pentanoic acid | 95975.26±756.77 | 83359.46±756.81 | 117136.29±983.05 | 90897.66±479.79 | 84247.98±486.28 | 94709.3±311.53 |
| 3-Methylbutanoic acid | 24957.41±37.95 | 24869.8±52.12 | 24034.24±41.1 | 22975.8±30.12 | 26543.63±107.63 | 23201.55±15.7 |
| Hexanoic acid | 32844.66±168.46 | 34635.55±261.12 | 52342.7±47.46 | 31765.9±43.81 | 47991.12±102.08 | 25778.33±60.63 |
| Octanoic acid | 19887.39±73.65 | 20526.58±118.39 | 21687.01±79.38 | 20350.2±145.05 | 20887.93±144.6 | 19643.15±139.56 |
| Decanoic acid | 9104.15±48.89 | 9214.45±96.14 | 9140.4±25.95 | 8958.73±31.21 | 9137.57±33.56 | 9059.88±88.78 |
| Dodecanoic acid | 1926.87±55.07 | 2664.22±13.88 | 1747.3±24.08 | 1108.59±15.36 | 1568.37±30.38 | 1737.82±53.36 |
| Benzoic acid | 18946.15±1661.74 | 43232.26±3002.23 | 83311.08±2157.62 | 34761.67±364.12 | 44184.81±3497.63 | 33332.8±1012.59 |
| L-Lactic acid^a^ | 855.8±0.9 | 763.21±0.95 | 727.35±1.19 | 1068±1.22 | 860.93±1.29 | 991.33±2.28 |
| Citric acid | 67420.53±101.28 | 60207.3±111.51 | 61099.3±152.43 | 45783±50.66 | 80664.7±165.82 | 70478.57±165.47 |
| Palmitic acid | 7092.5±51.03 | 13698.5±113.08 | 9082±103.7 | 5603.5±18.37 | 8647±10.61 | 7223±23.68 |
| Oleic acid | 6528.5±25.72 | 8708.5±10.21 | 3743.5±25.72 | 5335.5±34.7 | 6409±22.86 | 6654.5±29.8 |
| Linoleic acid | 2955.5±42.05 | 4982.5±91.86 | 3001.5±30.62 | 2072±14.7 | 3194±16.33 | 2841±22.86 |
| Acetaldehyde^a^ | 497.78±8.51 | 601.63±25.68 | 1213.29±45.41 | 549.5±3.09 | 697.85±44.12 | 550.63±13.33 |
| 2-Methylpropanal | 8881.5±367.18 | 8056.84±533.25 | 13933.98±858.86 | 9123.12±204.66 | 6998.02±496.89 | 8928.86±121.76 |
| 2-Methylbutanal | 9776.67±269.79 | 9012.7±263.18 | 11353.85±293.58 | 9866.96±282.27 | 6927.3±304.08 | 9756.02±103.2 |
| 3-Methylbutanal | 54710.07±1518.81 | 67477.89±1596.54 | 69276.24±753.01 | 66820.7±1328.93 | 51514.4±1582.64 | 57766.52±444.41 |
| Nonanal | 88.9±6.69 | 22.89±0.86 | 40.12±3.29 | 65.86±7.24 | 84.12±3.74 | 56.3±6.79 |
| Benzaldehyde | 2811.08±189.76 | 4699.68±182.13 | 2870.28±199.61 | 4001.47±251.16 | 3461.1±124.72 | 3601.37±270.61 |
| Benzeneacetaldehyde | 19951±520.97 | 10876.1±354.53 | 24047.5±2361.35 | 16438.85±1320.4 | 11702.28±452.2 | 5861.98±163.65 |
| Diethoxymethane | 4292.38±380.92 | 14216.54±347.55 | 32567.27±484.38 | 9316.94±217.47 | 16523.46±350.14 | 7919.1±430.7 |
| 1,1-Diethoxyethane | 384331.81±15960.52 | 461474.82±4046.81 | 895722.71±16901.71 | 420124.43±1623.05 | 522934.83±7541.89 | 407525.36±26861.03 |
| 1,1-Diethoxy-3-methylbutane | 36487.81±986.91 | 47000.27±3370.72 | 46012.04±1699.83 | 45431.7±957.93 | 34141.44±4140.32 | 37514.4±3361.37 |
| (2,2-Diethoxyethyl)-benzene | 91.62±8.51 | 81.31±1.29 | 194.36±4.61 | 25.81±1.66 | 50.03±5.33 | 20.39±0.71 |
| 1,1,3,3-Tetraethoxy-propane | 23.38±0.14 | 107.81±1.35 | 342.02±20.28 | 38.42±1.53 | 45.13±3.5 | 15.7±0.74 |
| Acetone | 23835.37±1984.75 | 35237.79±2077.31 | 45417.69±2599.39 | 32692.32±467.38 | 28764.39±1731.78 | 26758.51±2752.9 |
| 2-Pentanone | 12796.16±950.32 | 16517.48±321.35 | 27328.62±999.49 | 15518.04±208.58 | 14386.53±73.93 | 13234.19±1120.9 |
| 3-Hydroxyl-2-butanone | 30369.13±2467.47 | 16010.71±888.52 | 15368.58±574.46 | 36030.29±1446.02 | 24353.72±1212.81 | 28361.68±1144.18 |
| 2-Heptanone | 646.65±10.32 | 369.66±24.83 | 703.26±66.93 | 541.29±38.56 | 374.52±28.13 | 692±10.8 |
| 2-Octanone | 0.51±0.03 | 1.21±0.01 | 0.39±0.02 | 0.45±0.01 | 0.9±0.1 | 0.61±0.02 |
| 3-Octanone | 25.33±2.72 | 49.81±5.34 | 65.03±0.14 | 39.49±1.55 | 35.99±0.98 | 33.44±1.42 |
| 2-Nonanone | 42.87±5.25 | 129.25±15.72 | 129.29±0.84 | 105.68±7.94 | 44.99±4.56 | 43.41±1.69 |
| 4-Nonanone | 9.03±0.24 | 36.68±4.54 | 44.92±3.05 | 29.37±2.91 | 53.86±1.3 | 48.76±4.54 |
| 2-Undecanone | 12.54±0.78 | 49.55±0.76 | 61.65±4.49 | 31.83±1.73 | 16.66±0.14 | 0±0 |
| 4-Undecanone | 272.39±18.34 | 313.35±9.2 | 793.22±53.08 | 510.84±19.15 | 0±0 | 0±0 |
| 2-Tridecanone | 14.92±1.09 | 25.83±1.28 | 32±1.05 | 17.78±1.45 | 9.37±0.98 | 0±0 |
| 2-Pentadecanone | 47.96±0.9 | 56.99±2.77 | 88.76±3.84 | 52.08±5.7 | 39.72±1.79 | 63.95±3.03 |
| Furfuryl alcohol | 21526.47±1149.81 | 12766.11±609.44 | 4323.87±69.17 | 18626.49±674.77 | 8055.23±291.13 | 27577.95±2899.67 |
| Furfural^a^ | 412.53±27.67 | 302.39±24.81 | 408.79±19.31 | 394.95±6.12 | 341.29±26.28 | 427.63±26.94 |
| 5-Methylfurfural | 1473.72±116.94 | 1095±8.06 | 1414.52±133.07 | 855.26±85.22 | 881.52±31.1 | 897.92±73.43 |
| Furfuryl ethyl ether | 5716.09±182.86 | 6742.33±62.34 | 3110.53±352.36 | 8240.48±51.5 | 4538.3±52.72 | 7808.86±152 |
| 2-Acetylfuran | 1343.08±139.61 | 1630.25±89.14 | 1859.4±221.71 | 1149.54±70.98 | 1299.52±103.96 | 798.45±91.14 |
| HDMF | 156.63±4.19 | 135.32±3.72 | 107.49±2.83 | 74.04±0.2 | 63.96±0.79 | 68.08±2.62 |
| HEMF | 4.94±0.67 | 13.73±0.89 | 31.44±16.87 | 0±0 | 0±0 | 26.15±0.67 |
| Sotolon | 36.07±1.31 | 38.39±0.98 | 120.62±7.45 | 40.88±1.26 | 31.94±0.22 | 30.06±1.43 |
| Pyrazine | 132.44±0.75 | 233.98±4.65 | 173.52±4.87 | 135.75±1.91 | 121.09±6.09 | 103.99±3.81 |
| 2-Methylpyrazine | 822.13±26.92 | 1269.03±20.55 | 1035.87±10.21 | 905.52±40.49 | 722.12±5.45 | 786.86±6.47 |
| 2,3-Dimethylpyrazine | 220.85±16.15 | 279.75±8.72 | 326.91±2.43 | 337.17±9.24 | 206±4.05 | 204.49±3.68 |
| 2,5-Dimethylpyrazine | 3011.08±120.75 | 5393.51±399.58 | 3680.22±183.63 | 3989.62±125.6 | 2962.97±73.94 | 2902.12±83.44 |
| 2,6-Dimethylpyrazine | 622.15±5.14 | 838.29±17.35 | 522.73±16.22 | 638.64±5.93 | 523.86±12.28 | 761.93±25.23 |
| 2-Ethylpyrazine | 202.51±10.54 | 325.52±7.84 | 325.1±8.19 | 263.19±4.86 | 193.09±13.14 | 223.27±11.43 |
| 2-Ethyl-3-methylpyrazine | 87.07±1.92 | 118.7±10.68 | 89.35±2.68 | 100.87±2.97 | 54.28±0.95 | 84.87±3.89 |
| 2-Ethyl-6-methylpyrazine | 2555.7±67.83 | 4149.13±253.14 | 5269.45±147.25 | 3378.05±166.11 | 2893.38±158.99 | 2700.01±102.15 |
| 2,3,5-Trimethylpyrazine | 3458.01±149.16 | 4139.86±245.79 | 4871.66±13.74 | 5351.36±166.16 | 3478.86±98.01 | 3622.84±175.65 |
| 2-Ethyl-3,5-dimethylpyrazine | 2261.16±157.55 | 3353.14±397.36 | 6677.2±274.86 | 3363.54±103.41 | 2738.2±75.44 | 1956.95±137.26 |
| 2-Ethyl-3,6-dimethylpyrazine | 360.86±13.79 | 305.6±4.6 | 169.71±5.09 | 314.15±8.71 | 171.8±1.97 | 253.06±1.95 |
| 2,3,5,6-Tetramethylpyrazine | 5452.34±188.73 | 5983.32±82.28 | 8117.5±382.29 | 13206.4±708.96 | 5467.7±148.32 | 4458.6±309.34 |
| 2,3-Diethylpyrazine | 875.28±34.86 | 80.14±4.67 | 68.41±4.2 | 49.6±3.91 | 8±0.69 | 46.86±3.62 |
| 2,3-Diethyl-5-methylpyrazine | 3.41±0.79 | 355.99±1.51 | 331.98±5.72 | 146.05±4.92 | 74.19±4.23 | 101.26±1.7 |
| 2-Isobutyl-3-methylpyrazine | 463.68±15.75 | 216.86±13.05 | 238.56±7.06 | 218.93±6.33 | 140.01±2.05 | 155.23±8.82 |
| (Trimethylpyrazin-2-yl)methanol | 0±0 | 22.57±0.18 | 32.06±0.44 | 24.58±0.78 | 16.8±0.54 | 14.1±0.33 |
| Dimethyl trisulfide | 190.31±11.31 | 318±3.43 | 267.4±2.75 | 362.45±28.79 | 145.73±10.42 | 180.18±13.88 |
| Phenol | 836.52±10.84 | 773.27±18.91 | 506.33±26.38 | 630.26±41.15 | 538.07±23.88 | 432.04±28.06 |
| p-Cresol | 322.2±16.39 | 801.49±11.46 | 1585.2±82.32 | 501.24±32.03 | 831.16±31.64 | 392.7±34.88 |
| Guaiacol | 1198.87±32.51 | 1035.49±2.74 | 349.72±42.72 | 526.75±1.34 | 452.44±20.8 | 525.43±4.25 |
| 4-Methylguaiacol | 32.4±2.91 | 6.75±0.07 | 24.48±1.53 | 21.24±0.51 | 28.33±3.14 | 30.91±3.78 |
| 4-Ethylguaiacol | 144.3±12.59 | 138.74±3.07 | 364.16±32.69 | 373.12±6.6 | 203.37±4.78 | 133.94±13.76 |
| 4-Vinylguaiacol | 0.83±0.01 | 0.99±0.04 | 1.32±0 | 2.25±0.09 | 6.13±0.24 | 1.56±0.04 |
| gama-Nonalactone | 234.98±5.9 | 183.42±0.87 | 158.15±4.56 | 157.61±8.9 | 139.03±7.64 | 159.24±14.05 |
| gama-Dodecalactone | 340.14±12.7 | 298±11.23 | 410.01±10.64 | 356.92±18.84 | 390.11±8.91 | 337.47±9.59 |
| Phytone | 59.36±0.96 | 92.18±5.29 | 143.43±3.96 | 88.39±8.53 | 65.41±1.3 | 87.76±3.27 |
| beta-Damascone | 6.53±0.06 | 4.35±0.15 | 5.64±0.06 | 8.53±0.75 | 1.73±0.1 | 7.09±0.63 |
| Geranylacetone | 19.38±0.26 | 29.91±1.4 | 23.01±0.78 | 32±1.23 | 10.44±0.59 | 18.96±2.11 |

Data represent mean ± SD (n = 3).

^a^The unit of compound is mg/L.

Table S7 Differential compounds screened from both sides of the Chishui River by orthogonal partial least squares-discriminant analysis (OPLS-DA)

| No. | Name | VIP value | Error |
| --- | --- | --- | --- |
| 1 | 1,1-Diethoxyethane | 7.19541 | 0.577035 |
| 2 | Ethyl formate | 3.73261 | 0.470984 |
| 3 | Ethyl hexanoate | 2.82363 | 0.595863 |
| 4 | 2-Butanol | 2.70531 | 0.629621 |
| 5 | 2-Methylpropanol | 2.37179 | 0.37023 |
| 6 | 3-Methylbutyl hexanoate | 2.34804 | 0.6935 |
| 7 | 3-Hydroxyl-2-butanone | 2.33469 | 0.784279 |
| 8 | Hexanoic acid | 2.30954 | 0.491657 |
| 9 | 2-Methylbutanol | 2.13813 | 0.396604 |
| 10 | 1-Hexanol | 1.69322 | 0.309547 |
| 11 | Ethyl butanoate | 1.672 | 0.354068 |
| 12 | (R,R)-2,3-Butanediol | 1.61959 | 0.299486 |
| 13 | Benzoic acid | 1.57401 | 0.192682 |
| 14 | Ethyl propanoate | 1.55269 | 0.383603 |
| 15 | 2-Methylpropanoic acid | 1.34895 | 0.231854 |
| 16 | Pentanoic acid | 1.27783 | 0.122758 |
| 17 | Propyl acetate | 1.26893 | 0.214155 |
| 18 | Benzeneacetaldehyde | 1.26096 | 0.337325 |
| 19 | 2,3,5,6-Tetramethylpyrazine | 1.24706 | 0.433972 |
| 20 | Propionic acid | 1.23825 | 0.618555 |
| 21 | 2-Pentanone | 1.22695 | 0.202334 |
| 22 | Diethoxymethane | 1.21408 | 0.224298 |
| 23 | Furfuryl alcohol | 1.1355 | 0.186767 |
| 24 | Ethyl 3-methylbutanoate | 1.1062 | 0.186728 |
| 25 | Ethyl linoleate | 1.05248 | 0.105071 |
| 26 | 3-Methylbutanoic acid | 1.02747 | 0.251805 |

Table S8 Differential compounds screened from four towns of the Chishui River by OPLS-DA

| No. | Name | VIP value | Error |
| --- | --- | --- | --- |
| 1 | 1,1-Diethoxyethane | 5.26443 | 0.340093 |
| 2 | Ethyl formate | 2.77439 | 0.25689 |
| 3 | Ethyl butanoate | 2.67937 | 0.358906 |
| 4 | 2-Methylpropanol | 2.67367 | 0.165121 |
| 5 | 3-Methylbutanal | 2.44816 | 0.484964 |
| 6 | 3-Hydroxyl-2-butanone | 2.30409 | 0.401781 |
| 7 | Ethyl propanoate | 2.23036 | 0.421829 |
| 8 | 3-Methylbutyl hexanoate | 2.17215 | 0.15711 |
| 9 | 1,1-Diethoxy-3-methylbutane | 2.15704 | 0.422663 |
| 10 | 2-Butanol | 2.14405 | 0.272348 |
| 11 | Ethyl hexanoate | 2.07268 | 0.360556 |
| 12 | 2-Methylpropanoic acid | 1.99079 | 0.282253 |
| 13 | Propionic acid | 1.90378 | 0.416603 |
| 14 | Pentanoic acid | 1.88797 | 0.190218 |
| 15 | Hexanoic acid | 1.73796 | 0.270653 |
| 16 | 2-Methylbutanol | 1.73175 | 0.149417 |
| 17 | 2-Phenethyl hexanoate | 1.63249 | 0.23871 |
| 18 | (R,R)-2,3-Butanediol | 1.55121 | 0.341634 |
| 19 | Benzeneacetaldehyde | 1.53728 | 0.440478 |
| 20 | Ethyl hexadecanoate | 1.4429 | 0.240136 |
| 21 | 2,3,5,6-Tetramethylpyrazine | 1.41694 | 0.276958 |
| 22 | Benzoic acid | 1.37598 | 0.212873 |
| 23 | Acetone | 1.35653 | 0.214074 |
| 24 | Furfuryl alcohol | 1.26191 | 0.503648 |
| 25 | 1-Hexanol | 1.2398 | 0.222837 |
| 26 | 1,2-Propanediol | 1.2352 | 0.235677 |
| 27 | Ethyl 2-hydroxy-hexanoate | 1.21986 | 0.518964 |
| 28 | Palmitic acid | 1.21595 | 0.138921 |
| 29 | 2-Pentanone | 1.21552 | 0.110311 |
| 30 | 2-Methylbutanal | 1.14901 | 0.366568 |
| 31 | Ethyl benzoate | 1.1454 | 0.240115 |
| 32 | Furfuryl ethyl ether | 1.08607 | 0.3564 |
| 33 | Propyl acetate | 1.07991 | 0.274411 |
| 34 | Ethyl linoleate | 1.05995 | 0.228556 |
| 35 | Butanoic acid | 1.04593 | 0.0594501 |
| 36 | Diethoxymethane | 1.0209 | 0.203318 |
| 37 | 3-Methylbutanoic acid | 1.01615 | 0.112145 |
| 38 | Ethyl pentanoate | 1.00928 | 0.404533 |
| 39 | Hexyl hexanoate | 1.00853 | 0.419323 |

Table S9 The Spearman correlation coefficients between aroma compounds and sensory attributes

| Sensory attributes | Aroma compounds | Correlation coefficient | *P* value | Relation |
| --- | --- | --- | --- | --- |
| Acidic | 1-Pentanol | -0.539450766 | 2.05391E-10 | negtive |
| Acidic | 1-Hexanol | -0.567754745 | 1.35864E-11 | negtive |
| Florar | Meso-2,3-Butanediol | -0.595168934 | 7.56521E-13 | negtive |
| Grain | 2-Methylpropanoic acid | -0.592272961 | 1.03975E-12 | negtive |
| Grain | 3-Methylbutanoic acid | -0.531489795 | 4.2168E-10 | negtive |
| Acidic | 3-Methylbutanoic acid | -0.597060687 | 6.13557E-13 | negtive |
| Qu | Hexanoic acid | -0.505072248 | 4.0325E-09 | negtive |
| Acidic | Hexanoic acid | -0.607868268 | 1.80535E-13 | negtive |
| Acidic | Octanoic acid | -0.548887537 | 8.54172E-11 | negtive |
| Qu | Acetaldehyde | -0.547835 | 9.43264E-11 | negtive |
| Acidic | Acetaldehyde | -0.581322861 | 3.36452E-12 | negtive |
| Qu | 1,1-Diethoxyethane | -0.504700519 | 4.15704E-09 | negtive |
| Acidic | 1,1-Diethoxyethane | -0.561457249 | 2.54216E-11 | negtive |
| Florar | 4-Undecanone | -0.53130306 | 4.28759E-10 | negtive |
| Florar | Furfuryl alcohol | -0.535712417 | 2.88598E-10 | negtive |
| Florar | Furfuryl ethyl ether | -0.648887177 | 1.10583E-15 | negtive |
| Acidic | Pyrazine | -0.547170992 | 1.00401E-10 | negtive |
| Florar | 2,3,5,6-Tetramethylpyrazine | -0.54086676 | 1.80367E-10 | negtive |
| Grain | p-Cresol | -0.593670216 | 8.9221E-13 | negtive |
| Acidic | p-Cresol | -0.567991075 | 1.32673E-11 | negtive |
| Florar | Geranylacetone | -0.628620293 | 1.50646E-14 | negtive |
| Qu | Ethyl formate | -0.509786608 | 2.73318E-09 | negtive |
| Acidic | Ethyl formate | -0.505196834 | 3.99157E-09 | negtive |
| Grain | Ethyl butanoate | -0.581800496 | 3.19943E-12 | negtive |
| Grain | Ethyl pentanoate | -0.506161009 | 3.68803E-09 | negtive |
| Qu | Ethyl hexanoate | -0.504040439 | 4.38734E-09 | negtive |
| Acidic | Ethyl hexanoate | -0.605689167 | 2.319E-13 | negtive |
| Acidic | Hexyl hexanoate | -0.510590716 | 2.55624E-09 | negtive |
| Qu | Ethyl 2-furancarboxylate | -0.51749912 | 1.42804E-09 | negtive |
| Acidic | Ethyl 2-furancarboxylate | -0.619841157 | 4.40545E-14 | negtive |
| Roasted | Ethyl phenylacetate | 0.508043836 | 3.15797E-09 | positive |
| Grain | Ethyl phenylacetate | 0.591821112 | 1.09233E-12 | positive |
| Grain | 1-Propanol | 0.527780236 | 5.8587E-10 | positive |
| Florar | 1-Pentanol | 0.551655593 | 6.56918E-11 | positive |
| Florar | 1-Hexanol | 0.519049263 | 1.2509E-09 | positive |
| Sauce | 3-Octanol | 0.512499397 | 2.17923E-09 | positive |
| Sauce | 2-Undecanol | 0.569441933 | 1.14611E-11 | positive |
| Qu | 2-Undecanol | 0.539207603 | 2.10012E-10 | positive |
| Grain | 2-Undecanol | 0.505912568 | 3.76405E-09 | positive |
| Acidic | 2-Undecanol | 0.526849189 | 6.35882E-10 | positive |
| Acidic | Meso-2,3-Butanediol | 0.516481586 | 1.55719E-09 | positive |
| Grain | 2-Phenethyl alcohol | 0.529267861 | 5.1373E-10 | positive |
| Acidic | 2-Phenethyl alcohol | 0.546964666 | 1.02365E-10 | positive |
| Sauce | Butanoic acid | 0.559506241 | 3.07855E-11 | positive |
| Qu | Butanoic acid | 0.542181836 | 1.59779E-10 | positive |
| Grain | Butanoic acid | 0.529185362 | 5.17496E-10 | positive |
| Acidic | Butanoic acid | 0.524350892 | 7.91237E-10 | positive |
| Grain | Pentanoic acid | 0.526408225 | 6.6098E-10 | positive |
| Qu | Oleic acid | 0.604039061 | 2.79959E-13 | positive |
| Florar | Acetaldehyde | 0.664896639 | 1.21693E-16 | positive |
| Grain | 2-Methylpropanal | 0.541224475 | 1.74527E-10 | positive |
| Grain | 2-Methylbutanal | 0.558624129 | 3.35553E-11 | positive |
| Roasted | 3-Methylbutanal | 0.546031344 | 1.11718E-10 | positive |
| Sauce | Benzaldehyde | 0.533158426 | 3.63224E-10 | positive |
| Roasted | Benzaldehyde | 0.627582541 | 1.71322E-14 | positive |
| Florar | Diethoxymethane | 0.62895008 | 1.44598E-14 | positive |
| Florar | 1,1-Diethoxyethane | 0.65406262 | 5.49745E-16 | positive |
| Roasted | 1,1-Diethoxy-3-methylbutane | 0.554838582 | 4.84268E-11 | positive |
| Grain | Furfuryl alcohol | 0.644052353 | 2.09892E-15 | positive |
| Grain | Furfuryl ethyl ether | 0.643936656 | 2.13106E-15 | positive |
| Acidic | Furfuryl ethyl ether | 0.538536725 | 2.23288E-10 | positive |
| Florar | Sotolon | 0.521364208 | 1.02515E-09 | positive |
| Florar | Pyrazine | 0.568883042 | 1.21268E-11 | positive |
| Sauce | 2,3,5-Trimethylpyrazine | 0.592661395 | 9.96519E-13 | positive |
| Qu | 2,3,5-Trimethylpyrazine | 0.50345679 | 4.60115E-09 | positive |
| Roasted | 2,3,5-Trimethylpyrazine | 0.603384352 | 3.0159E-13 | positive |
| Sauce | 2,3,5,6-Tetramethylpyrazine | 0.621786898 | 3.48307E-14 | positive |
| Qu | 2,3,5,6-Tetramethylpyrazine | 0.611042855 | 1.24925E-13 | positive |
| Roasted | 2,3,5,6-Tetramethylpyrazine | 0.673838777 | 3.34106E-17 | positive |
| Acidic | 2,3,5,6-Tetramethylpyrazine | 0.559048769 | 3.21932E-11 | positive |
| Sauce | (Trimethylpyrazin-2-yl)methanol | 0.540557168 | 1.85574E-10 | positive |
| Roasted | (Trimethylpyrazin-2-yl)methanol | 0.5793532 | 4.13672E-12 | positive |
| Roasted | Dimethyl trisulfide | 0.610382267 | 1.34919E-13 | positive |
| Florar | p-Cresol | 0.598686428 | 5.11922E-13 | positive |
| Florar | 4-Methylguaiacol | 0.505321671 | 3.95096E-09 | positive |
| Acidic | gama-Nonalactone | 0.575213038 | 6.35813E-12 | positive |
| Florar | gama-Nonalactone | 0.540449666 | 1.87415E-10 | positive |
| Qu | beta-Damascone | 0.618807961 | 4.98749E-14 | positive |
| Florar | beta-Damascone | 0.505466898 | 3.90421E-09 | positive |
| Qu | Geranylacetone | 0.557355111 | 3.7966E-11 | positive |
| Roasted | Geranylacetone | 0.51043391 | 2.58986E-09 | positive |
| Acidic | Geranylacetone | 0.589150907 | 1.45979E-12 | positive |
| Florar | Ethyl formate | 0.617087229 | 6.12627E-14 | positive |
| Florar | 2-Phenylethyl acetate | 0.50243555 | 4.99954E-09 | positive |
| Grain | Propyl acetate | 0.533584755 | 3.4959E-10 | positive |
| Florar | Ethyl 2-methylbutanoate | 0.532198374 | 3.95827E-10 | positive |
| Florar | Ethyl 3-methylbutanoate | 0.534422656 | 3.24218E-10 | positive |
| Qu | Ethyl 4-methylpentanoate | 0.524058229 | 8.11665E-10 | positive |
| Roasted | Ethyl tridecanoate | 0.64144531 | 2.9513E-15 | positive |
| Qu | Ethyl linoleate | 0.555898396 | 4.37202E-11 | positive |
| Florar | Ethyl 2-furancarboxylate | 0.644987347 | 1.85593E-15 | positive |
| Acidic | Ethyl 2-hydroxy-4-methylpentanoate | 0.529813372 | 4.89485E-10 | positive |
| Grain | Ethyl 2-hydroxy-hexanoate | 0.501875398 | 5.23192E-09 | positive |

Table S10 Four reconstruction experimental schemes for verifying key aroma compounds in SABs (μg/L)

| NO. | Name | Rec A | | Rec B | | Rec C | | Rec D | |
| --- | --- | --- | --- | --- | --- | --- | --- | --- | --- |
|  |  | MT-1 | EL-1 | MT-1 | EL-1 | MT-1 | EL-1 | MT-1 | EL-1 |
| 1 | Ethyl acetate^a^ | 2889.84 | 2352.52 | 2889.84 | 2352.52 | 2889.84 | 2352.52 | 2889.84 | 2352.52 |
| 2 | Propyl acetate | 7904.52 | 5646.32 | 7904.52 | 5646.32 | 7904.52 | 5646.32 | 7904.52 | 5646.32 |
| 3 | 3-Methylbutyl acetate | 3137.79 | 6901.76 | 3137.79 | 6901.76 | 3137.79 | 6901.76 | 3137.79 | 6901.76 |
| 4 | Ethyl propanoate | 30741.94 | 58782.56 | 30741.94 | 58782.56 | 30741.94 | 58782.56 | 30741.94 | 58782.56 |
| 5 | Ethyl 2-methylpropanoate | 16957.6 | 15013.93 | 16957.6 | 15013.93 | 16957.6 | 15013.93 | 16957.6 | 15013.93 |
| 6 | Ethyl butanoate | 39891.75 | 48385.34 | 39891.75 | 48385.34 | 39891.75 | 48385.34 | 39891.75 | 48385.34 |
| 7 | 3-Methylbutyl butanoate | 219.32 | 461.65 | 219.32 | 461.65 | 219.32 | 461.65 | 219.32 | 461.65 |
| 8 | Ethyl 2-methylbutanoate | 3323.91 | 7470.19 | 3323.91 | 7470.19 | 3323.91 | 7470.19 | 3323.91 | 7470.19 |
| 9 | Ethyl 3-methylbutanoate | 11607.69 | 21439.67 | 11607.69 | 21439.67 | 11607.69 | 21439.67 | 11607.69 | 21439.67 |
| 10 | Ethyl pentanoate | 3586.68 | 12623.64 | 3586.68 | 12623.64 | 3586.68 | 12623.64 | 3586.68 | 12623.64 |
| 11 | Ethyl hexanoate | 31313.06 | 92079.05 | 31313.06 | 92079.05 | 31313.06 | 92079.05 | 31313.06 | 92079.05 |
| 12 | 3-Methylbutyl hexanoate | 5354.7 | 714.34 | 5354.7 | 714.34 | 5354.7 | 714.34 | 5354.7 | 714.34 |
| 13 | 2-Phenethyl hexanoate | 2421.97 | 5163.11 | 2421.97 | 5163.11 | 2421.97 | 5163.11 | 2421.97 | 5163.11 |
| 14 | Ethyl octanoate | 1416.45 | 1615.05 | 1416.45 | 1615.05 | 1416.45 | 1615.05 | 1416.45 | 1615.05 |
| 15 | Ethyl decanoate | 163.76 | 1618.94 | 163.76 | 1618.94 | 163.76 | 1618.94 | 163.76 | 1618.94 |
| 16 | Ethyl tetradecanoate | 1227.71 | 1045.81 | 1227.71 | 1045.81 | 1227.71 | 1045.81 | 1227.71 | 1045.81 |
| 17 | Ethyl hexadecanoate | 32435.16 | 37663.87 | 32435.16 | 37663.87 | 32435.16 | 37663.87 | 32435.16 | 37663.87 |
| 18 | Ethyl linoleate | 17759.19 | 11946.17 | 17759.19 | 11946.17 | 17759.19 | 11946.17 | 17759.19 | 11946.17 |
| 19 | Ethyl lactate^a^ | 2115.68 | 2223.81 | 2115.68 | 2223.81 | 2115.68 | 2223.81 | 2115.68 | 2223.81 |
| 20 | Ethyl 2-hydroxy-4-methylpentanoate | 13871.55 | 11598.72 | 13871.55 | 11598.72 | 13871.55 | 11598.72 | 13871.55 | 11598.72 |
| 21 | Ethyl benzoate | 14177.15 | 14341.18 | 14177.15 | 14341.18 | 14177.15 | 14341.18 | 14177.15 | 14341.18 |
| 22 | Ethyl phenylacetate | 4467.4 | 1839.22 | 4467.4 | 1839.22 | 4467.4 | 1839.22 | 4467.4 | 1839.22 |
| 23 | 1-Propanol^a^ | 1391.37 | 1614.64 | 1391.37 | 1614.64 | 1391.37 | 1614.64 | 1391.37 | 1614.64 |
| 24 | 1-Butanol^a^ | 111.81 | 76.71 | 111.81 | 76.71 | 111.81 | 76.71 | 111.81 | 76.71 |
| 25 | 2-Methylpropanol^a^ | 161.46 | 218.82 | 161.46 | 218.82 | 161.46 | 218.82 | 161.46 | 218.82 |
| 26 | 1-Pentanol | 9996.91 | 13631.85 | 9996.91 | 13631.85 | 9996.91 | 13631.85 | 9996.91 | 13631.85 |
| 27 | 3-Methylbutanol^a^ | 257.11 | 467.96 | 257.11 | 467.96 | 257.11 | 467.96 | 257.11 | 467.96 |
| 28 | 1-Hexanol | 5179.4 | 27479.92 | 5179.4 | 27479.92 | 5179.4 | 27479.92 | 5179.4 | 27479.92 |
| 29 | 2-Heptanol | 3628.27 | 3467.84 | 3628.27 | 3467.84 | 3628.27 | 3467.84 | 3628.27 | 3467.84 |
| 30 | 1-Octen-3-ol | 284.66 | 183.75 | 284.66 | 183.75 | 284.66 | 183.75 | 284.66 | 183.75 |
| 31 | Acetic acid^a^ | 1834.53 | 1604.65 | 1834.53 | 1604.65 | 1834.53 | 1604.65 | 1834.53 | 1604.65 |
| 32 | Propionic acid | 49703.31 | 68720.65 | 49703.31 | 68720.65 | 49703.31 | 68720.65 | 49703.31 | 68720.65 |
| 33 | Butanoic acid | 29172.29 | 26657.51 | 29172.29 | 26657.51 | 29172.29 | 26657.51 | 29172.29 | 26657.51 |
| 34 | 2-Methylpropanoic acid | 34341.25 | 45329.12 | 34341.25 | 45329.12 | 34341.25 | 45329.12 | 34341.25 | 45329.12 |
| 35 | Pentanoic acid | 92068.57 | 90569.49 | 92068.57 | 90569.49 | 92068.57 | 90569.49 | 92068.57 | 90569.49 |
| 36 | 3-Methylbutanoic acid | 18165.32 | 25303.39 | 18165.32 | 25303.39 | 18165.32 | 25303.39 | 18165.32 | 25303.39 |
| 37 | Hexanoic acid | 16899.92 | 57737.93 | 16899.92 | 57737.93 | 16899.92 | 57737.93 | 16899.92 | 57737.93 |
| 38 | Octanoic acid | 19321.18 | 22392.77 | 19321.18 | 22392.77 | 19321.18 | 22392.77 | 19321.18 | 22392.77 |
| 39 | Benzoic acid | 33999.97 | 48578.47 | 33999.97 | 48578.47 | 33999.97 | 48578.47 | 33999.97 | 48578.47 |
| 40 | Acetaldehyde^a^ | 305.81 | 810.89 | 305.81 | 810.89 | 305.81 | 810.89 | 305.81 | 810.89 |
| 41 | 2-Methylpropanal | 5700.92 | 4656.92 | 5700.92 | 4656.92 | 5700.92 | 4656.92 | 5700.92 | 4656.92 |
| 42 | 2-Methylbutanal | 7077.55 | 2469.16 | 7077.55 | 2469.16 | 7077.55 | 2469.16 | 7077.55 | 2469.16 |
| 43 | 3-Methylbutanal | 37991.04 | 23605.13 | 37991.04 | 23605.13 | 37991.04 | 23605.13 | 37991.04 | 23605.13 |
| 44 | Nonanal | 587.59 | 26.24 | 587.59 | 26.24 | 587.59 | 26.24 | 587.59 | 26.24 |
| 45 | Benzeneacetaldehyde | 15033.22 | 15862.2 | 15033.22 | 15862.2 | 15033.22 | 15862.2 | 15033.22 | 15862.2 |
| 46 | 1,1-Diethoxyethane | 236321.6 | 590570.84 | 236321.6 | 590570.84 | 236321.6 | 590570.84 | 236321.6 | 590570.84 |
| 47 | 1,1-Diethoxy-3-methylbutane | 25306.3 | 14819.51 | 25306.3 | 14819.51 | 25306.3 | 14819.51 | 25306.3 | 14819.51 |
| 48 | Acetone | 25527.03 | 21449.85 | 25527.03 | 21449.85 | 25527.03 | 21449.85 | 25527.03 | 21449.85 |
| 49 | 2-Pentanone | 6168.75 | 24116.8 | 6168.75 | 24116.8 | 6168.75 | 24116.8 | 6168.75 | 24116.8 |
| 50 | 3-Hydroxyl-2-butanone | 30122.82 | 986.15 | 30122.82 | 986.15 | 30122.82 | 986.15 | 30122.82 | 986.15 |
| 51 | Furfuryl alcohol | 25727.16 | 4128.44 | 25727.16 | 4128.44 | 25727.16 | 4128.44 | 25727.16 | 4128.44 |
| 52 | Furfural^a^ | 227.94 | 193.54 | 227.94 | 193.54 | 227.94 | 193.54 | 227.94 | 193.54 |
| 53 | Furfuryl ethyl ether | 11896.15 | 3528.47 | 11896.15 | 3528.47 | 11896.15 | 3528.47 | 11896.15 | 3528.47 |
| 54 | HDMF | 33 | 88.23 | 33 | 88.23 | 33 | 88.23 | 33 | 88.23 |
| 55 | Sotolon | 27.94 | 79.36 | 27.94 | 79.36 | 27.94 | 79.36 | 27.94 | 79.36 |
| 56 | 2-Ethyl-6-methylpyrazine | 1802.58 | 2827.28 | 1802.58 | 2827.28 | 1802.58 | 2827.28 | 1802.58 | 2827.28 |
| 57 | 2,3,5-Trimethylpyrazine | 5140.43 | 1696.06 | 5140.43 | 1696.06 | 5140.43 | 1696.06 | 5140.43 | 1696.06 |
| 58 | 2-Ethyl-3,5-dimethylpyrazine | 2411.04 | 1814.24 | 2411.04 | 1814.24 | 2411.04 | 1814.24 | 2411.04 | 1814.24 |
| 59 | 2-Ethyl-3,6-dimethylpyrazine | 271.32 | 263.04 | 271.32 | 263.04 | 271.32 | 263.04 | 271.32 | 263.04 |
| 60 | 2,3-Diethyl-5-methylpyrazine | 116.98 | 185.96 | 116.98 | 185.96 | 116.98 | 185.96 | 116.98 | 185.96 |
| 61 | 2-Isobutyl-3-methylpyrazine | 156.89 | 127.19 | 156.89 | 127.19 | 156.89 | 127.19 | 156.89 | 127.19 |
| 62 | Dimethyl trisulfide | 230.78 | 27.69 | 230.78 | 27.69 | 230.78 | 27.69 | 230.78 | 27.69 |
| 63 | p-Cresol | 682.99 | 811.56 | 682.99 | 811.56 | 682.99 | 811.56 | 682.99 | 811.56 |
| 64 | Guaiacol | 112.6 | 787.44 | 112.6 | 787.44 | 112.6 | 787.44 | 112.6 | 787.44 |
| 65 | 4-Methylguaiacol | 681.43 | 17.71 | 681.43 | 17.71 | 681.43 | 17.71 | 681.43 | 17.71 |
| 66 | 4-Ethylguaiacol | 10.11 | 216.69 | 10.11 | 216.69 | 10.11 | 216.69 | 10.11 | 216.69 |
| 67 | 4-Vinylguaiacol | 280.53 | 8.39 | 280.53 | 8.39 | 280.53 | 8.39 | 280.53 | 8.39 |
| 68 | gama-Nonalactone | 417.62 | 84.77 | 417.62 | 84.77 | 417.62 | 84.77 | 417.62 | 84.77 |
| 69 | gama-Dodecalactone | 511.74 | 373.38 | 511.74 | 373.38 | 511.74 | 373.38 | 511.74 | 373.38 |
| 70 | beta-Damascone | 18.5 | 1 | 18.5 | 1 | 18.5 | 1 | 18.5 | 1 |
| 71 | 2-Phenylethyl acetate |  |  | 35.61 | 20.62 | 35.61 | 20.62 | 35.61 | 20.62 |
| 72 | Ethyl 4-methylpentanoate |  |  | 228.87 | 191.21 | 228.87 | 191.21 | 228.87 | 191.21 |
| 73 | Ethyl tridecanoate |  |  | 56.37 | 32.31 | 56.37 | 32.31 | 56.37 | 32.31 |
| 74 | Ethyl 2-furancarboxylate |  |  | 126.66 | 1465.69 | 126.66 | 1465.69 | 126.66 | 1465.69 |
| 75 | 3-Octanol |  |  | 155.52 | 63.67 | 155.52 | 63.67 | 155.52 | 63.67 |
| 76 | 2-Undecanol |  |  | 79.65 | 41.64 | 79.65 | 41.64 | 79.65 | 41.64 |
| 77 | Meso-2,3-Butanediol |  |  | 13947.65 | 7667.8 | 13947.65 | 7667.8 | 13947.65 | 7667.8 |
| 78 | 2-Phenethyl alcohol |  |  | 10353.76 | 8754.36 | 10353.76 | 8754.36 | 10353.76 | 8754.36 |
| 79 | Oleic acid |  |  | 8613 | 5264 | 8613 | 5264 | 8613 | 5264 |
| 80 | 4-Undecanone |  |  | 218.51 | 159.98 | 218.51 | 159.98 | 218.51 | 159.98 |
| 81 | Pyrazine |  |  | 62.6 | 120.42 | 62.6 | 120.42 | 62.6 | 120.42 |
| 82 | (Trimethylpyrazin-2-yl)methanol |  |  | 51.04 | 0 | 51.04 | 0 | 51.04 | 0 |
| 83 | Ethyl formate |  |  |  |  | 37918.15 | 129888.21 | 37918.15 | 129888.21 |
| 84 | Ethyl 2-hydroxy-hexanoate |  |  |  |  | 38400.79 | 15703.27 | 38400.79 | 15703.27 |
| 85 | 2-Methylbutanol |  |  |  |  | 46786.28 | 80285.57 | 46786.28 | 80285.57 |
| 86 | 1,2-Propanediol |  |  |  |  | 30247.73 | 31829.64 | 30247.73 | 31829.64 |
| 87 | (R,R)-2,3-Butanediol |  |  |  |  | 50154.28 | 44306.07 | 50154.28 | 44306.07 |
| 88 | Palmitic acid |  |  |  |  | 10775 | 16296.5 | 10775 | 16296.5 |
| 89 | 2,3,5,6-Tetramethylpyrazine |  |  |  |  | 14170.94 | 1964.25 | 14170.94 | 1964.25 |
| 90 | L-Lactic acid^a^ |  |  |  |  |  |  | 1736.32 | 1731.61 |
| 91 | Citric acid^a^ |  |  |  |  |  |  | 29.76 | 10.31 |
| 92 | Linoleic acid |  |  |  |  |  |  | 3335 | 4381.5 |

^a^The unit of compound is mg/L.
